# Massively parallel interrogation of human functional variants modulating cancer immunosurveillance

**DOI:** 10.1038/s41392-025-02171-5

**Published:** 2025-03-19

**Authors:** Ying Liu, Yongshuo Liu, Xuran Niu, Ang Chen, Yizhou Li, Ying Yu, Binrui Mo, Zhiheng Liu, Tao Xu, Jie Cheng, Zeguang Wu, Wensheng Wei

**Affiliations:** 1 Biomedical Pioneering Innovation Center, Beijing Advanced Innovation Center for Genomics, Peking-Tsinghua Center for Life Sciences, Peking University Genome Editing Research Center, State Key Laboratory of Gene Function and Modulation Research, School of Life Sciences, Peking University, Beijing, China; 2 Changping Laboratory, Beijing, China; 3 Department of Clinical Laboratory, Shandong Cancer Hospital and Institute, Shandong First Medical University and Shandong Academy of Medical Sciences, Jinan, Shandong China; 4 Academy for Advanced Interdisciplinary Studies, Peking University, Beijing, China; 5 Department of pathology, School of Basic Medicine, Tongji Medical College and State Key Laboratory for Diagnosis and Treatment of Severe Zoonotic Infectious Diseases, Huazhong University of Science and Technology, Wuhan, Hubei China

**Keywords:** Functional genomics, Functional genomics, Cancer genomics, Tumour immunology, Tumour biomarkers

## Abstract

Anti-PD-1/PD-L1 immune checkpoint blockade (ICB) therapy has revolutionized clinical cancer treatment, while abnormal PD-L1 or HLA-I expression in patients can significantly impact the therapeutic efficacy. Somatic mutations in cancer cells that modulate these critical regulators are closely associated with tumor progression and ICB response. However, a systematic interpretation of cancer immune-related mutations is still lacking. Here, we harnessed the ABEmax system to establish a large-scale sgRNA library encompassing approximately 820,000 sgRNAs that target all feasible serine/threonine/tyrosine residues across the human genome, which systematically unveiled thousands of novel mutations that decrease or augment PD-L1 or HLA-I expression. Beyond residues associated with phosphorylation events, our screens also identified functional mutations that affect mRNA or protein stability, DNA binding capacity, protein-protein interactions, and enzymatic catalytic activity, leading to either gene inactivation or activation. Notably, we uncovered certain mutations that concurrently modulate PD-L1 and HLA-I expression, represented by the clinically relevant mutation SETD2_Y1666. We demonstrated that this mutation induces consistent phenotypic effects across multiple cancer cell lines and enhances the efficacy of immunotherapy in different tumor models. Our findings provide an unprecedented resource of functional residues that regulate cancer immunosurveillance, offering valuable guidance for clinical diagnosis, ICB therapy, and the development of innovative drugs for cancer treatment.

## Introduction

In the course of cancer development and progression, tumors adopt a variety of strategies to evade immune surveillance and suppress antitumor immune responses. These mechanisms include the activation of inhibitory immune checkpoints, dysfunction of antigen processing and presentation (APP), and the editing of immunogenic neoantigens, which also present challenges to effective cancer treatment.^1,2^ Recent advances in cancer immunotherapies, particularly ICB therapies, have achieved remarkable efficacy in clinical trials across various malignancies, which have revolutionized cancer treatment paradigms. Among the immune checkpoints, the PD-1/PD-L1 pathway has stood out as an appealing target due to its considerable therapeutic potential and relatively low immune-related toxicity. Several PD-1/PD-L1 blockade antibodies have been approved for the treatment of various cancers, including melanoma, non-small cell lung cancer, and renal cell carcinoma, demonstrating encouraging clinical outcomes.^3,4^ Despite these successes, therapeutic responses remain limited to a small subset of patients, and the underlying mechanisms driving this variation are not yet fully elucidated.^1,4,5^ A series of tumor-intrinsic factors have been identified as critical determinants of the immunotherapy outcomes, especially in relation to the PD-L1 signaling pathway, major histocompatibility complex class I (MHC-I)-mediated APP, and interferon-γ (IFNγ) signaling in the tumor microenvironment (TME). These regulatory pathways directly influence antitumor activity and affects the efficacy of PD-1/PD-L1 blockade therapy by modulating the ability of immune cells to recognize and destroy tumor cells.^6^ Nonetheless, there is still a pressing need for a more comprehensive understanding of the regulatory factors that govern both the response and resistance against ICB therapy.

Genetic screening has proven to be an effective tool in the identification of potential targets and regulators in cancer immunology. In recent years, the application of CRISPR-based functional genomics screens has significantly advanced our understanding of the molecular regulators of PD-L1 and MHC-I (HLA-I for human) in cancer cells, providing key insights into the molecular landscape of tumor immune surveillance.^7–11^ However, these traditional CRISPR/Cas9 screens are limited in their resolution and provide information primarily at the gene level, which can be insufficient for understanding the functional impacts of specific mutations or genetic variations. Somatic mutations in cancer cells can profoundly impact critical pathways related to immune regulation and have been shown to be closely associated with clinical responses to ICB treatment.^12–14^ Notably, according to the International Cancer Genome Consortium (ICGC) database, single-nucleotide variants (SNVs) are predominant and account for over 90% among varied types of somatic mutations. Despite their widespread occurrence, the functional relevance of these SNVs remains largely unexplored. With the development of base editing technologies, high-throughput functional screens based on base editing have revolutionized the canonical screening strategy, enabling the assessment of variant functions at the level of individual bases or amino acids.^15,16^ A recent study used base editing screens to map mutations of key mediators of IFNγ pathway, providing an initial resource for understanding IFNγ signaling in cancer immune surveillance.^17^ Nevertheless, a vast number of mutations with unknown functional significance still require systematic investigation to decode their roles in immunoregulation and the response to ICB therapy.

In clinical settings involving anti-PD-1/PD-L1 antibody treatments, the expression levels of PD-L1 or HLA-I on the surface of cancer cells in patients have been shown to have predictive value for the effectiveness of ICB therapy.^6,18–21^ Ongoing research has demonstrated that post-translational modifications (PTMs) play pivotal roles in controlling PD-L1 expression and antigen presentation, through various mechanisms, such as regulating the protein stability, translocation, and protein-protein interactions.^20,22^ Among the hundreds of types of PTMs, phosphorylation is the most common and extensively studied, primarily occurring on serine (S), threonine (T), and tyrosine (Y) residues in eukaryotes.^23^ Protein phosphorylation can broadly impact immune-related oncogenic or inflammatory signaling pathways, such as JAK/STAT, RAS, MAPK, and NF-*κ*B pathways, thus affecting the anti-tumor immune response.^22^ However, despite the potential significance of phosphorylation sites, only a limited number of these sites have been thoroughly characterized in the context of cancer immunosurveillance.

In this study, we aimed to systematically identify critical sites involved in cancer immunosurveillance and the response to ICB therapy, focusing on potential phosphorylation sites on S/T/Y residues. Using an ABEmax-based sgRNA library coupled with the iBAR strategy,^24^ we targeted all S/T/Y codons across the entire human genome. Through multiple high-throughput variant screens for regulators modulating PD-L1 and HLA-I expression, we identified thousands of novel residues within known regulatory genes as well as previously uncharacterized genes, shedding light on their roles in the individual regulation and co-regulation for PD-L1 and HLA-I expression. We then assessed the regulatory mechanisms of one representative site, SETD2_Y1666, a clinically relevant mutation that modulates the expression of both PD-L1 and HLA-I. Additionally, we validated its effects across various cell lines and evaluated its potential to enhance the response to ICB therapy in different tumor models. Our study provides an unprecedented resource of functional residues for understanding the cancer immune response. Furthermore, the findings offer valuable insights for clinical diagnosis and the optimization of ICB treatment.

## Results

### Genome-wide mapping of critical S/T/Y residues modulating PD-L1 expressions by ABE-based screening

The interaction between PD-L1 on tumor cells and PD-1 on T cells impedes activation, proliferation, and effector functions of antigen-specific CD8 + T cells, thus promoting cancer immune evasion.^6^ To systematically explore the functional residues modulating PD-L1, the core factor involved in immunotherapy, we leveraged ABEmax to generate site-directed mutagenesis for achieving large-scale screens. Our recent work has established an ABE-based sgRNA library targeting all feasible protein-coding regions containing S/T/Y residues within the editing window leading to missense mutations. This library encompasses a staggering 818,619 sgRNAs, which collectively target 277,051 S, 165,599 T, and 141,687 Y residues.^25^ The de novo synthesized S/T/Y library consists of two sub-libraries–one targeting the sense strand (465,554 sgRNAs) and the other one targeting the antisense strand (354,595 sgRNAs). Both sub-libraries were supplemented with the same negative controls targeting the *AAVS1* locus. To better handling such an extensive library effectively, the sgRNA library was constructed with three internal barcodes (iBARs) (hereinafter referred to as sgRNA^iBAR^ library), as previously described.^24^ This system ensures a high-quality screening even at a high multiplicity of infection (MOI) while significantly reducing the number of cells required for the screening process.

**Fig. 1:**
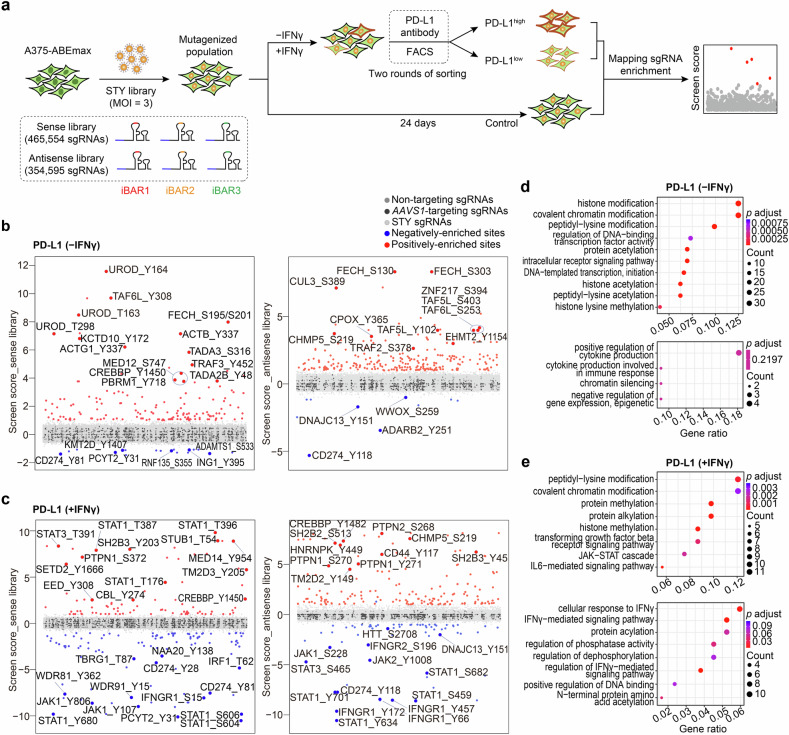
ABE-based screens identify functional S/T/Y residues modulating PD-L1 expression at the genome-wide level. **a** Schematic overview of the ABE screens for identifying S/T/Y residues that regulate PD-L1 expression with and without IFNγ stimulation in A375 cells. **b**, **c** Significant S/T/Y residues enriched from the sense library (sense lib, left) and antisense library (antisense lib, right) that upregulate or downregulate PD-L1 expression in the absence of IFNγ (**b**) and upon IFNγ treatment (**c**). Positively or negatively enriched sites were selected based on a screen score > 1 or < −1. **d**, **e** Gene ontology (GO**)** enrichment analysis of related genes haboring identified mutations that lead to PD-L1 upregulation (upper panels) and downregulation (lower panels) in the absence of IFNγ (d) and upon IFNγ treatment (**e**)

PD-L1 expression can be driven by tumor-intrinsic mechanisms or induced by inflammatory cytokines, such as IFNγ, which is secreted by immune cells within the TME.^3^ To probe functional residues affecting cell surface PD-L1 expression in both constitutive and induced contexts, we performed screens using the S/T/Y sgRNA^iBAR^ library in a human melanoma cell line, A375, which was engineered to stably express ABEmax. This cell line exhibits low level of endogenous PD-L1 but shows substantial upregulation of PD-L1 upon exposure to IFNγ (Supplementary Fig. 1a). The two S/T/Y sub-libraries were separately transduced into A375-ABEmax cells at an MOI of 3. Subsequently, following ten days of sgRNA transduction, the library cells were subjected to both IFNγ-stimulated and non-stimulated conditions. Through two rounds of fluorescence-activated cell sorting (FACS) enrichment, we collected cell populations with either lower or higher level of surface PD-L1 expression in each condition (Fig. 1a; Supplementary Fig. 1e–h). We also maintained a control group of library cells without FACS selection throughout the positive screening process (Supplementary Fig. 1b–d). The library cells from the control group and FACS-selected experimental groups were subjected to next-generation sequencing (NGS), and the NGS data was subsequently analyzed using the MAGeCK-iBAR algorithm.^24^ This analysis involved evaluating the change in sgRNA abundance and calculating the *P* value for each sgRNA, considering the significance and consistency of three iBARs per sgRNA in each screen. The screen score was then generated as −log_10_ of the *P* value after Benjamini-Hochberg (BH) adjustment (Fig. 1a).

We selected sgRNAs with a screen score >1 for further investigations. In each screen, numerous novel sites were identified in both the high and low directions of regulating PD-L1 expression (Fig. 1b, c; Supplementary Tables 1–4). To obtain a holistic understanding of the functional residues identified, we initially performed a gene ortholog (GO) analysis for all the related genes enriched in the screens, focusing on biological process. In the PD-L1 screen without IFNγ stimulation, the dominate terms in the PD-L1^high^ group were associated with histone modification, covalent chromatin modification, and the regulation of DNA-binding transcription factor activity. In contrast, the representative terms in PD-L1^low^ group included positive regulation of cytokine production and chromatin silencing (Fig. 1d). In the PD-L1 screen with IFNγ exposure, the enriched terms were significantly correlated with interferon stimulation, encompassing processes such as the JAK-STAT cascade, transforming growth factor signaling pathway, cellular response to IFNγ, and the regulation of phosphatase activity. Moreover, some terms overlapped between the IFNγ-treated and IFNγ-absent conditions, particularly in PD-L1^high^ group, where terms such as peptidyl-lysine modification and covalent chromatin modification indicated the presence of conserved factors involved in tumor-intrinsic PD-L1 regulation, regardless of IFNγ treatment (Fig. 1e).

### Massively parallel validation of regulatory variants affecting PD-L1 in A375 cells

**Fig. 2:**
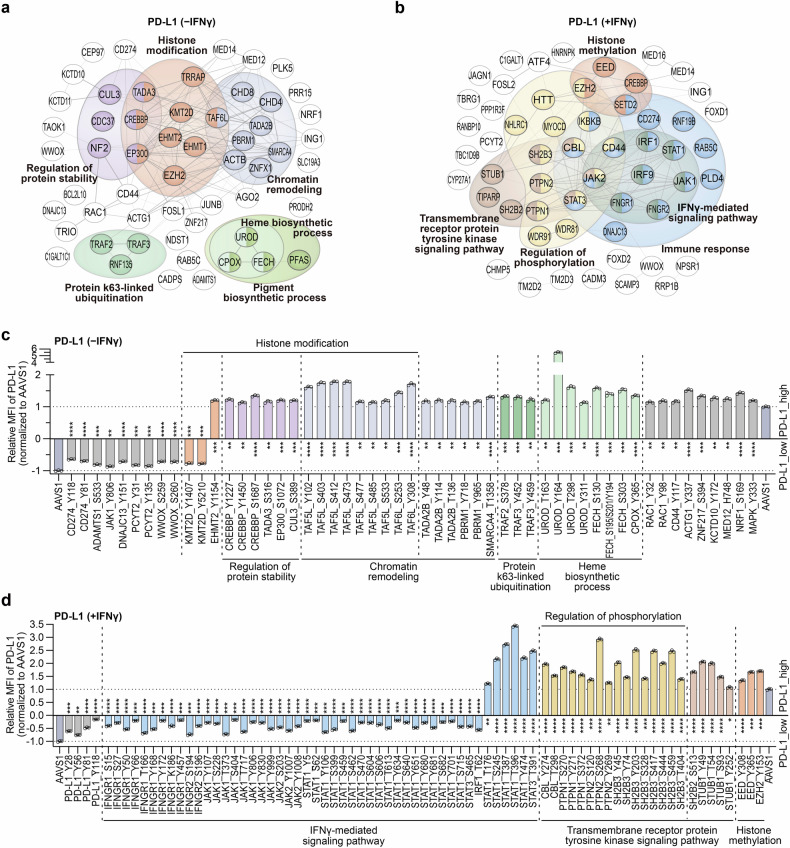
Validation of regulatory residues of PD-L1 enriched in various pathways. **a**, **b** STRING analysis of related genes harboring top-ranked mutations identified in the PD-L1 screens in the absence of IFNγ (**a**) and upon IFNγ treatment (**b**). **c**, **d** Individual validations of negative and postive regulators of cell surface PD-L1 in A375 cells in the absence of IFNγ (**c**) and upon IFNγ treatment (**d**) by flow cytometry analysis. Cell surface PD-L1 expression was analyzed following incubation without or with 100 ng/mL IFNγ for 48 h. The relative median fluorescence intensity (MFI) of surface PD-L1 for each mutant represents the ratio normalized to the MFI of *AAVS1*-targeting control cells. Data are presented as the mean ± SD (n = 3). *P* values were calculated using two-tailed Student’s *t* test, *P < 0.05, ** *P* < 0.01, ***P < 0.001, ****P < 0.0001; n.s., not significant

For a deeper insight into the top-ranked hits from the screens, we integrated representative genes from both high and low directions in each screen. Subsequently, we built protein-protein interaction (PPI) networks using STRING followed by GO analyses. In IFNγ-absent PD-L1 screen, the network prominently showcased multiple genes enriched in processes related to histone modification, regulation of protein stability, chromatin remodeling, and heme biosynthesis process (Fig. 2a). In the IFNγ-treated group, a large portion of genes were enriched in terms such as IFNγ-mediated signaling pathway, regulation of phosphorylation, and immune response (Fig. 2b).

To verify the regulatory roles of the identified variants, we selected candidate sites involved in different pathways and individually transduced each targeting sgRNA into A375-ABEmax cells via lentiviral infection. Subsequently, we conducted flow cytometry analysis to assess surface PD-L1 levels without or with IFNγ stimulation. Compared with the negative control sgRNA targeting the *AAVS1* locus, most of the sites showed significant regulation of PD-L1 expression.

In the absence of IFNγ, a standout performer was the UROD_Y164 site, alongside other confirmed residues within the UROD protein, including T163, T298 and Y311 (Fig. 2c). UROD is involved in the heme synthesis pathway, whose disruption has been recognized to lead to an increase in PD-L1 expression.^8^ Besides UROD, we also successfully verified the functionality of several mutations in FECH and CPOX, the other two core factors participating in heme synthesis but with no reported roles in regulating PD-L1 expression. Additionally, a series of novel sites enriched on genes associated with chromatin remolding, especially TAF5L and TAF6L, the integral components of the PCAF histone acetylase complex, were prominently ranked in the validation process (Fig. 2c). Further analysis indicated that most of these variants showing a noteworthy phenotype influenced the expression of the target genes, ultimately resulting in an upregulation of overall and surface PD-L1 levels (Supplementary Fig. 2a). In the PD-L1^low^ group, due to its low baseline PD-L1 expression, a relatively smaller number of sites were identified and subjected to validation. Notably, PD-L1_Y118 and Y81 displayed the most significant impact, with Y118 being a previously recognized phosphorylation site. We also verified their association with PD-L1 expression for several additional sites, which are linked to genes known to be involved in immune response or ICB, such as WWOX_S259 and KMT2D_Y1407^26,27^ (Fig. 2c).

Regarding the IFNγ-stimulated condition, several mutants reducing PD-L1 expression in IFNγ-absent condition were also validated under IFNγ treatment, including CD274_Y118, which exerted the strongest effect on downregulating surface PD-L1, consistent with the screening results (Fig. 2d). Meanwhile, with IFNγ stimulation, more sites were identified and verified within these functional genes, such as *WWOX* and *PCYT2* (Supplementary Fig. 2b). A systematic analysis revealed that numerous mutations reduced the protein levels of their respective coding genes, as observed in the PD-L1^high^ group with *STUB1*, and in the PD-L1^low^ group with *WWOX*, *TBRG1*, and *IKBKB* (Supplementary Fig. 2c, d). Moreover, there were variants that did not significantly affect their protein expression, including HNRNPK_Y449, EED_Y308, and EED_Y365, suggesting that they may induce PD-L1 expression through other mechanisms (Supplementary Fig. 2c). Remarkably, a substantial number of sites were enriched on genes linked to the IFNγ-mediated signaling pathway and regulation of phosphorylation (Fig. 2d; Supplementary Fig. 2b), we thus delved into investigating the regulatory mechanisms of these candidate sites.

### Systematic combing of functional residues within the IFNγ signaling pathway

We observed plenty of novel sites emerged on well-established genes linked to the IFNγ-mediated signaling pathway, including IFNγ receptors, Janus kinases, among others. Most of these mutations negatively regulated PD-L1 expression by affecting their respective coding genes, such as *IFNGR1*, *IFNGR2*, *JAK1*, *JAK2*, and *IRF1*. All investigated mutations on IFNGR1 and IFNGR2 were found to simultaneously decrease the total and membrane PD-L1 protein levels (Fig. 2d; Supplementary Fig. 3a). Notably, IFNGR1_Y457, a known phosphorylation site responsible for mediating the interaction between IFNGR1 and STAT1 proteins,^28^ was found to significantly downregulate PD-L1 expression. This suggests that the IFNGR1_Y457H mutation might affect its binding with STAT1, blocking the transmission of IFNγ signals and resulting in a substantial reduction in PD-L1 expression. Similarly, all tested mutations in the downstream non-receptor tyrosine kinases JAK1 and JAK2 led to a reduction in both the total and membrane protein levels of PD-L1 (Supplementary Fig. 3b, c). Among these, two mutations on JAK2 consistently reduced both the mRNA and protein levels of JAK2, while most verified sites on JAK1 did not affect its own expression at both the mRNA and protein levels (Supplementary Fig. 3c, d). In addition, multiple sites on JAK1 and JAK2 were closely associated with phosphorylation, as exemplified by four known phosphorylation sites and two predicted phosphorylation sites on JAK1, and two conserved phosphorylation sites, Y1007 and Y1008, on JAK2, which are critical for JAK2 function^29^ (Supplementary Fig. 3b).

**Fig. 3:**
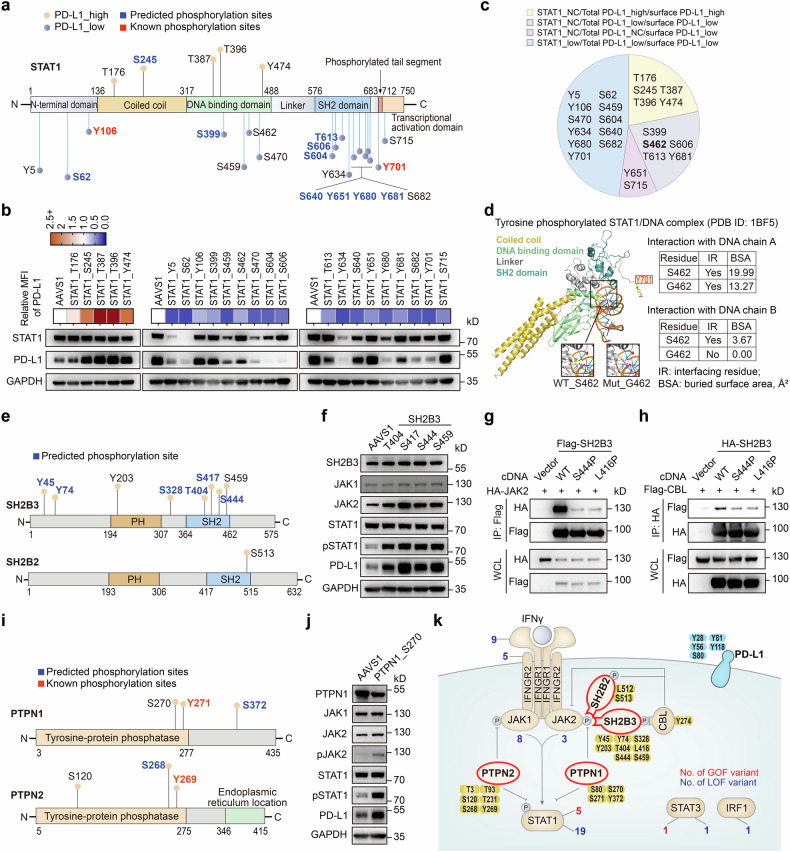
Novel residues on canonical and non-canonical regulatory proteins involved in IFNγ signal transduction affect PD-L1 expression. **a** Distribution of identified S/T/Y residues on STAT1 protein. **b** Protein expression levels of STAT1 and PD-L1 in the indicated A375 mutant cells treated with IFNγ. The upper heatmap shows the relative surface PD-L1 level of A375 cells with each mutation, based on the results of flow cytometric analysis from Fig. 2d. The lower IB analysis shows the total protein level of STAT1 and PD-L1 for each corresponding mutant. **c** Pie chart of STAT1 residues that are classfied based on their differential regulation of STAT1, total PD-L1, and surface PD-L1 expression. **d** Schematic of the molecular structure and intramolecular interactions around the STAT1_S462 residue within tyrosine phosphorylated STAT1 and DNA complex (PDB: 1BF5). The WT S462 or the mutated G462 residue is labeled in yellow (left). The table shows the interaction between STAT1_S462/G462 and DNA chain A/B, indicated by the parameters of interfacing residue (IR) and buried surface area (BSA) (right). **e** Distribution of identified S/T/Y residues on SH2B2 and SH2B3 proteins. **f** IB analysis of typical JAK/STAT signaling components, SH2B3, and PD-L1 in A375 cells infected with sgRNAs targeting *AAVS1* and each mutation, respectively. **g** IB analysis of anti-Flag immunoprecipitates (IPs) and whole-cell lysates (WCLs) of HEK293T cells co-transfected with the indicated plasmids expressing HA-tagged JAK2 and Flag-tagged SH2B3 WT or variants. **h** IB analysis of anti-HA IPs and WCLs of HEK293T cells co-transfected with the indicated plasmids expressing Flag-tagged CBL and HA-tagged SH2B3 WT or variants. **i** Distribution of identified S/T/Y residues on PTPN1 and PTPN2 proteins. **j** IB analysis of typical JAK/STAT signaling components, PTPN1, and PD-L1 in A375 cells infected with sgRNAs targeting *AAVS1* and PTPN1_S270, respectively. **k** Schematic diagram of SH2-B family proteins and PTP family proteins regulating the IFNγ-induced JAK/STAT signaling pathway. The S/T/Y residues identified in the screens are labeled on the corresponding proteins. For (**a**, **e**, and **i**), the regulatory residues are marked in two directions on the protein structure: mutations above the line indicate negative regulators and mutations below the line indicate positive regulators. The relative length of each vertical line reflects the regulatory effect of the indicated residue, based on the results of flow cytometric analysis from Fig. 2d. All cell samples were treated with 100 ng/mL IFNγ for 48 h

Intriguingly, some genes related to the IFNγ signaling pathway contained residues with both negative and positive regulatory roles in PD-L1 expression. Notably, *STAT1* and *STAT3* were identified in this context (Fig. 2d), which could not be detected in canonical screens at the gene level. STAT1, an important transcription factor connecting cytokine receptors with downstream target genes, is involved in the signaling of many cytokines, including IFNγ. In our screening, numerous functional S/T/Y sites were identified on the STAT1 protein, distributed across its four domains as well as the coiled-coil region (Fig. 3a). Among them, five mutations were confirmed to upregulate PD-L1 expression, with two in the coiled-coil region and three in the DNA binding domain. The majority of mutations appeared to inhibit PD-L1 expression and were dispersed across functional regions, including the N-terminal domain, DNA-binding domain, SH2 domain, phosphorylated tail segment, and the transcriptional activation domain. One of the well-known sites was STAT1_Y701, located in the phosphorylated tail segment, where phosphorylation is required for the dimerization and nucleation of STAT1.^30^ Besides Y701, we also identified another confirmed phosphorylation site, STAT1_Y106, and 9 predicted phosphorylation sites that resulted in decreased PD-L1 expression following mutation, among which 7 sites were in the SH2 domain, indicating a close relationship between the SH2 domain and phosphorylation-mediated signaling transmission.

We further performed immunoblot (IB) verification for all the selected sites within STAT1. Five PD-L1^high^ variants consistently upregulated PD-L1 expression in both total and membrane protein levels, while leaving the STAT1 protein level unchanged (Fig. 3b). We hypothesized that these variants represent gain-of-function (GOF) mutations that promote the shuttle of STAT1 into the nucleus, facilitating its binding to DNA. Conversely, the majority of PD-L1^low^ mutations, distributed across various domains of STAT1, had an inhibitory effect on STAT1 expression, resulting in a reduction in both total and membrane protein levels of PD-L1. Interestingly, nearly half of these variants had no discernible impact on STAT1 expression. Some of them only affected the membrane PD-L1 levels, leaving the total PD-L1 level unchanged. This category includes mutations such as Y651 and S715. Others induced PD-L1 reduction in both total and membrane protein levels (Fig. 3c). One notable example in this category is STAT1_S462G, a variant located in the DNA binding domain of STAT1, which was speculated to destroy the interaction between STAT1 and DNA. To verify this conjecture, we investigated the interaction between STAT1 and DNA before and after S462 mutation using the PDBePISA website (https://www.ebi.ac.uk/pdbe/pisa/). The analysis revealed that STAT1_S462 has interface contact with both strands of DNA, indicating that S462 is located at the interaction interface. However, S462G mutation completely abolished the ability of STAT1 to interact with one strand of DNA and decreased the contact area with the other DNA strand (Fig. 3d). The analysis indicated that S462G mutation is likely to reduce the binding capacity of STAT1 to DNA, weakening its transcriptional effect and ultimately affecting the expression level of PD-L1.

### Critical S/T/Y residues within adaptor proteins and tyrosine phosphatases participate in the regulation of PD-L1 expression

In addition to the proteins directly involved in the IFNγ signaling pathway mentioned above, a series of mutations were associated with the regulation of phosphorylation. Among them, multiple residues on two types of proteins, which belong to the Src homology 2-B (SH2-B) protein family and the protein tyrosine phosphatase (PTP) protein family, were significantly enriched in the PD-L1^high^ screen. It’s worth noting that, for most of these proteins, their relevance in PD-L1 regulation especially at the residue level has not been intensively investigated in previous studies.

The SH2-B family, comprising SH2B1, SH2B2 (APS), and SH2B3 (Lnk),^31^ is a conserved family of adaptor proteins with similar structural characteristics. They possess a Pleckstrin homology domain (PH) that recognizes phosphatidylinositol lipids, enabling protein transfer to the cell membrane, as well as an SH2 domain that recognizes phosphorylated tyrosine residues (Fig. 3e). In human cells, SH2-B proteins recognize and bind to phosphorylated Y813 of JAK2 via their SH2 domains,^32,33^ and an active region at the C-terminal of these proteins gets phosphorylated, interacting with the tyrosine kinase binding (TKB) domain of the intracellular E3 ubiquitin ligase CBL (c-cbl). This interaction recruits CBL to the vicinity of JAK2, leading to the degradation of JAK2 through ubiquitination modification, thereby negatively regulating the IFNγ signaling pathway.^34^

We found that the residues with the most significant effects were clustered in the SH2 domain of these proteins, one for SH2B2 and four for SH2B3 (Fig. 3e). For SH2B3, all four mutations did not alter the protein level of SH2B3 but significantly increased the total abundance of PD-L1 protein. These mutations were found to activate the IFNγ-induced JAK-STAT signaling pathway, with JAK2 showing increased abundance and pSTAT1 levels significantly elevated (Fig. 3f). The overall pattern of SH2B2_S513 closely resembled that of SH2B3 (Supplementary Fig. 3e), suggesting that both proteins influence JAK-STAT signaling by regulating the protein abundance of JAK2. Additionally, CBL_Y274 was identified and verified in the study, which located within the TKB domain and closely related to the recognition of SH2-B family.^34^ Its regulation on downstream JAK-STAT signaling was consistent with that of SH2B2 and SH2B3 (Supplementary Fig. 3f), further highlighting the critical role of the “JAK2-adaptor-CBL” loop in regulating IFNγ-mediated JAK/STAT signaling pathway and PD-L1 expression.

To further understand the regulatory mechanisms of these mutations, we focused on the SH2 domain and selected representative sites, namely SH2B3_S417, S444, and SH2B2_S513, for further investigation. Genomic sequencing indicated that targeting SH2B3_S417 generated L416P mutation, SH2B3_S444 generated the expected S444P mutation, and SH2B2_S513 targeting generated S513P and the bystander mutation L512P (Supplementary Material). Consequently, we overexpressed both the wild-type (WT) cDNAs and all the corresponding mutant sequences of these two genes to perform co-immunoprecipitation (Co-IP) experiments with JAK2 and CBL, respectively. Both the L416P and S444P mutations in SH2B3 simultaneously disrupted the interaction between SH2B3 and JAK2, as well as CBL, with a particularly notable impact on the interaction with JAK2 (Fig. 3g, h). This severe destruction in the interactions with both JAK2 and CBL resulted in a weakened ubiquitination degradation of JAK2, leading to JAK2 upregulation and enhanced IFNγ signal, ultimately promoting PD-L1 expression. Intriguingly, distinct from the residues in SH2B3, neither the SH2B2_L512P nor the L513P single mutant, as well as the L512P/S513P double mutant, affected the interaction between SH2B2 and JAK2. However, these mutations significantly reduced the interaction between SH2B2 and CBL (Supplementary Fig. 3g, h). We speculated that SH2B3_L416 and S444 are located close to the interaction center where the SH2 domain binds to JAK2_Y813,^34^ while not for SH2B2_S512 or S513, thereby leading to a clear disruption in the interaction between SH2B3 and JAK2 after mutation. The analysis above suggests that mutations, especially those occurring within the SH2 domain of these two adaptor proteins, can dramatically affect the IFNγ signaling pathway, albeit through different regulatory patterns.

The screens also identified functional residues within PTPN1 and PTPN2, two members of the PTP family known to negatively regulate the cytokine signaling pathway through dephosphorylation of phosphorylated tyrosine residues on targeted proteins.^35,36^ Most of the identified residues were enriched within the phosphatase domain of each protein (Fig. 3i). As such, we speculated that these mutations might affect their phosphatase activity, thereby enhancing the transmission of IFNγ signal.

We noticed that PTPN1_S270/Y271 and PTPN2_S268/Y269 are homologues residues, implying that they might exert their regulatory effects through similar mechanisms. We selected PTPN1_S270 and PTPN2_S268 as representatives and confirmed that their respective targeting sgRNAs generated the expected mutations with minimal bystander editing (Supplementary Material). To verify the function of these dominant mutations, we separately overexpressed the WT cDNA and the corresponding mutants in A375 cells. Introduction of PTPN1_S270P or PTPN2_S268P variant decreased the expression of PTPN1 or PTPN2, respectively. This, in turn, resulted in an upregulation of PD-L1 in both total and membrane protein levels (Supplementary Fig. 3i). To comprehensively investigate the regulation of these two endogenous mutations in A375 cells, we focused on examining typical proteins involved in IFNγ signaling pathway. Both mutations increased the protein levels of JAK2, subsequently leading to a significant upregulation in pSTAT1 levels without changing the overall abundance of STAT1 protein (Fig. 3j; Supplementary Fig. 3j). These results suggest that PTPN1_S270P and PTPN2_S268P activate the IFNγ pathway by reducing the abundance of each respective protein, ultimately resulting in an increased pSTAT1 level and, consequently, an upregulation of PD-L1 expression. Similarly, we found that multiple mutations identified in PTPN1 and PTPN2 also led to a reduction in their own protein levels and an increase in the total and membrane PD-L1 abundance (Supplementary Fig. 3k; Fig. 2d). For these loss-of-function (LOF) mutations, their subsequent effects were consistent with the outcomes of knocking out PTPN1 or PTPN2 using the CRISPR/Cas9 system (Supplementary Fig. 3l).

We summarized the critical S/T/Y residues within the SH2-B and PTP family proteins to illustrate their regulatory effects on the canonical IFNγ pathway (Fig. 3k). Additionally, we evaluated the impact of representative residues involved in the IFNγ pathway on surface PD-L1 expression in additional human cancer cell lines, including the melanoma cell line A875, the sarcoma cell line HT1080, and the breast cancer cell line MCF-7, all of which were engineered to stably express ABEmax protein. In A875 cells, all selected variants showed the same phenotypes as those observed in A375 cells, suggesting the broad relevance of these functional sites across melanoma cell lines. In HT1080 and MCF-7 cells, most variants on STAT1, SH2B3, SH2B2 and PTPN2 were confirmed as functional, with the exception of SH2B3 and SH2B2 variants, which showed little or no effects in MCF-7 cells (Supplementary Fig. 4). These findings suggest that most variants retain functionality across different tumor backgrounds.

Based on the screening and validation results, in combination with prior related studies, we have created a gene network diagram outlining PD-L1 modulation at the single amino acid level (Supplementary Fig. 5). The rich information of functional residues contributes to a better understanding of the roles played by these corresponding proteins and provides initial insights for refining the PD-L1 regulatory network from a single residue perspective.

### Genome-wide mapping of critical residues modulating HLA-I expression using S/T/Y library

Tumor cells can employ various strategies to evade immune surveillance. In addition to increase the expression of immune checkpoint ligands, defects in MHC-I-mediated antigen processing and presentation (APP) can directly hinder the tumor recognition of CD8 + T cells and restrain their activation and proliferation.^37^ Genetic mutations in essential genes involved in MHC-I APP have been implicated in tumor progression and the development of resistance to ICB therapy.^13,38^ Therefore, beyond interpreting the regulation of PD-L1 pathway, it is also crucial to understand the regulatory mechanisms of MHC-I in tumor cells.

**Fig. 4:**
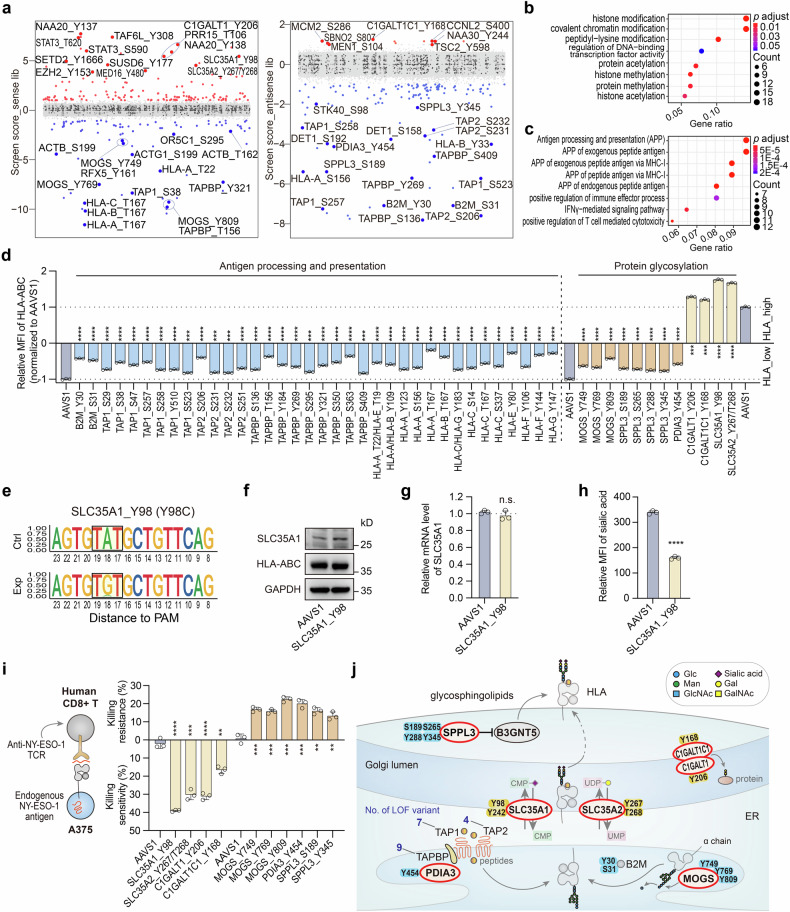
ABE-based screens identify functional S/T/Y residues modulating HLA-I expression at the genome-wide level. **a** Significant S/T/Y residues enriched from the sense library (left) and antisense library (right) that upregulate or downregulate HLA-I expression in A375 cells without IFNγ stimulation. The screening procedure is the same as that in Fig. 1a. Positively or negatively enriched sites were selected based on a screen score > 1 or < −1. **b**, **c** GO enrichment analysis of related genes haboring identified mutations that lead to HLA-I upregulation (**b**) and downregulation (**c**) in the absence of IFNγ. **d** Individual validations of representative sites related to APP and protein glycosylation in A375 cells in the absence of IFNγ by flow cytometry analysis. The method to generate relative MFI of HLA-ABC are the same as those shown in Fig. 2c, d. **e** Editing outcomes of sgRNA targeting SLC35A1_Y98 by NGS analysis. **f** Protein expression levels of SLC35A1 and HLA-ABC in A375 cells infected with sgRNAs targeting *AAVS1* and SLC35A1_Y98, respectively. **g** Relative mRNA expression levels of *SLC35A1* in A375 cells infected with respective sgRNAs targeting *AAVS1* and SLC35A1_Y98, respectively. The mRNA level of each sample was quantified by real-time qPCR and normalized to *GAPDH*. The indicated relative mRNA level of each sample was normalized to that of *AAVS1*-targeting control cells. **h** Relative MFI of surface sialic acid in A375 cells infected with sgRNAs targeting *AAVS1* and SLC35A1_Y98 by flow cytometry analysis. Data were normalized to that of the isotype. **i** Killing resistance and sensitivity of A375 cells infected with sgRNAs targeting residues on glycosylation-related genes to expanded anti-NY-ESO-1 CD8 + T cells. **j**, Schematic diagram of the HLA-I regulatory network focused on identified residues on representative APP and glycosylation-related genes. For (**d**, **g**, **h**, and **i**), data are presented as the mean ± SD (n = 3). *P* values were calculated using two-tailed Student’s *t* test, **P < 0.01, ***P < 0.001, ****P < 0.0001; n.s., not significant

We thus performed an additional S/T/Y library screen to investigate the functional residues that modulate HLA-I expression in A375 cells. Using the pan-human HLA-I-specific antibody W6/32 for protein staining, we observed a relatively high level of surface HLA-I expression in A375 cells without IFNγ stimulation, which enables to identify both positive and negative regulators of HLA-I expression in this context (Supplementary Fig. 6a). Consequently, we conducted the library screen for HLA-I regulators in A375-ABEmax cells in the absence of IFNγ. Through the same procedure of FACS enrichment and data analysis as described for the PD-L1 screen (Fig. 1a; Supplementary Fig. 6b, c), we identified numerous sites within genes related to APP that were prominent in HLA^low^ cells (Fig. 4a). These regulators included multiple allelic variants of HLA, the TAP binding protein TAPBP (tapasin), antigen transporters TAP1 and TAP2, and the component of MHC-I complex, B2M. In the HLA^high^ group, we also observed novel sites enriched on several negative regulators of HLA-I, including SUSD6_Y177 and WWP2_Y704, whose coding genes were recently reported to form an HLA-I inhibitory axis (SUSD6/TMEM127/WWP2) for cancer immune evasion.^39^

Upon integrating all the relevant genes identified through the screens, a GO analysis of biological process revealed that several terms similar to those from the PD-L1 screens were among the top-ranked in the HLA^high^ group. These terms included processes related to histone modification, covalent chromatin modification, and peptidyl-lysine modification, highlighting the general regulatory influence of genes on both PD-L1 and HLA-I (Fig. 4b). In contrast, in the HLA^low^ group, multiple terms related to antigen processing and presentation were highly enriched (Fig. 4c). STRING PPI network analysis of top-ranked regulators further revealed genes involved in antigen processing and presentation, immune response, and cellular protein modification process (Supplementary Fig. 6d). Of note, we identified several nodes connecting multiple networks, such as HLA-A, B2M, indicating their central roles in regulating the expression of each respective protein.

### Interpretation of novel residues regulating antigen recognition and presentation

Compared with the candidate sites identified in the PD-L1 screens, the HLA-I screens revealed a multitude of unique variants with unknown functions that were enriched in both high and low directions, not limited to sites within APP-related genes. To further assess their impact on surface HLA-I expression, we conducted a large-scale verification of candidate sites enriched on various regulatory pathways (Fig. 4d; Supplementary Fig. 7a).

In the HLA-I screens, a dominant category of functional sites enriched in APP-related genes. Nearly all relevant residues were subjected to individual validation, all of which were verified to significantly reduce surface HLA-I expression upon targeted mutation (Fig. 4d). For the gene *B2M*, two noteworthy sites, Y30 and S31 located in its Ig-like C1-type domain (Supplementary Fig. 7b), showed significant phenotypic effects. Targeting each of these two sites led to a double mutation, Y30H and S31P, which led to reduced levels of both membrane and total HLA, while B2M expression remained unaltered (Supplementary Fig. 7c, d). As for the *TAP1* and *TAP2* genes, multiple hits within TAP1 were mainly localized in its N-terminal domain and ABC transmembrane type-1 domain, and functional residues of TAP2 converged on its ABC transmembrane type-1 domain (Supplementary Fig. 7e). Unlike the mutants of B2M, several top-ranked mutations of TAP1 not only disrupted its own expression but also further reduced HLA expression at both total and membrane protein levels (Supplementary Fig. 7f). As for *TAPBP* (Supplementary Fig. 6e), we investigated eight mutants with significant phenotype of reduced surface HLA levels, among which five mutations slightly reduced the overall HLA expression and three had no significant effect on total HLA levels (Supplementary Fig. 7g).

In particular, we identified a significant number of mutations in glycosylation-related genes that were enriched in both HLA^high^ and HLA^low^ groups. Among these, targeting SLC35A1_Y98 with ABEmax led to a dramatic increase in surface HLA expression (Fig. 4d), which was further verified to result in the generation of the Y98C mutation (Fig. 4e). SLC35A1 is a membrane-bound transporter located in the Golgi apparatus responsible for transferring CMP-sialic acid from the cytosol into the Golgi apparatus. This process facilitates the sialylation of proteins by various sialyltransferases. Importantly, the Y98C mutation did not interfere with the expression of SLC35A1 at both the protein and mRNA levels (Fig. 4f, g). The residue Y98 is involved in the binding of CMP and CMP-sialic acid and is essential for optimal transport competence, as confirmed by previous in vitro studies.^40^ We thus examined the sialic acid level on the cell membrane and found that SLC35A1_Y98C mutation significantly impaired the transport of sialic acid (Fig. 4h). To further explain the relevance between SLC35A1 mutation and HLA abundance, we assessed the total HLA level in SLC35A1_Y98 mutant cells. Intriguingly, we found that the mutation increased the median fluorescence intensity (MFI) of surface HLA without changing overall HLA expression (Fig. 4d, e). Prior study has reported that sialic acid residues on glycosphingolipid (GSL), synthesized by B3GNT5, is involved in shielding critical epitopes of HLA-I molecules on the cell surface, thus diminishing their interactions with several immune cell receptors and decreasing CD8 + T cell responses.^41^ We thus infer that the change in sialic acid modification generated by SLC35A1_Y98 mutation may affect the accessibility of epitope recognition of HLA-I specific antibodies.

Similarly, several sites were identified in additional glycosylation-related genes. These genes included negative regulators, such as another nucleotide sugar transporter called SLC35A2 responsible for transporting UDP-galactose, as well as the glycosyltransferase C1GALT1 and its chaperone C1GALT1C1. On the positive regulatory side, there were genes like *SPPL3*, which negatively regulates B3GNT5 expression, thereby controlling GSL synthesis.^41^ Additionally, MOGS (α-glucosidase I), which encodes the first enzyme responsible for trimming N-glycans in the endoplasmic reticulum (ER),^42^ and PDIA3 (ERp57), which is involved in the general glycoprotein folding process within the ER and is required for optimal tapasin activity,^43^ also had identified sites. While most of the residues identified in these glycosylation-related genes exhibited no effect on the overall HLA-I expression, they significantly influenced surface HLA-I levels (Supplementary Fig. 7h). Therefore, it is likely that these residues can affect the glycosylation of various regulators or perturb the structural stability of glycoproteins involved in APP process.

To evaluate the role of these residues in immunosurveillance, we investigated the susceptibility of representative mutants, particularly those related to glycosylation, to CD8 + T cell-mediated cytotoxicity. We generated each mutation in A375-ABEmax cells, which endogenously express HLA-A2 and NY-ESO-1 antigen. Human CD8 + T cells transduced with an HLA-A2-restricted T cell receptor (TCR) specific for the NY-ESO-1 antigen^44^ were co-cultured with the mutant cells. We found that the HLA^high^ variants were more sensitive to T cell-driven killing, with SLC35A1_Y98 being an example. In contrast, the HLA^low^ mutations conferred significant resistance to T cell killing, thus subverting T-cell-mediated immunosurveillance (Fig. 4i; Supplementary Fig. 7i). These novel residues have been summarized for their functional roles in different glycosylation processes (Fig. 4j), which can alter the glycosylation of related regulatory genes or impact the quality control machinery of glycoprotein involved in the assembly of HLA-I molecules, consequently affecting the recognition of tumor cells by T cells.

### Integrated analysis for potential co-regulators of surface PD-L1 and HLA-I

**Fig. 5:**
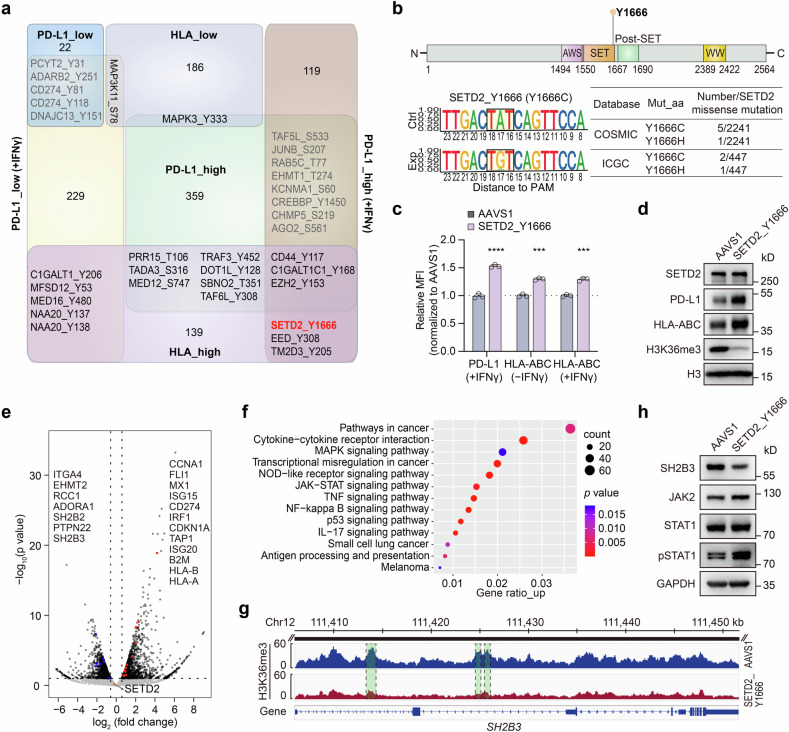
Interpretation of functional residues that co-regulate surface PD-L1 and HLA-I. **a** Comparison of S/T/Y residues identified in PD-L1 screens and HLA-I screens using venn diagram. **b** General information of SETD2_Y1666. The upper structure schematic indicates the location of Y1666 residue on SETD2 protein. The lower figures (left) indicate the editing outcomes of sgRNA targeting SETD2_Y1666 by NGS analysis. The lower table (right) indicates the information of clinical relevance of SETD2_Y1666. **c** Relative MFI of surface PD-L1 and HLA-I in A375 cells infected with sgRNAs targeting *AAVS1* and SETD2_Y1666 with different IFNγ treatment. The method to generate relative MFI of PD-L1 or HLA-I and the statistics are the same as those shown in Fig. 2c, d. **d** Protein expression levels of SETD2, PD-L1, HLA-ABC and H3K36me3 in A375 cells infected with sgRNAs targeting *AAVS1* and SETD2_Y1666, respectively. **e** Volcano plots showing the DEGs between SETD2_Y1666-targeted A375 mutant cells and *AAVS1*-targeted A375 control cells. The represented genes are listed. **f** Representative KEGG pathway analysis of upregulated DEGs in SETD2_Y1666-targeted A375 mutant cells compared with the *AAVS1*-targeted control. The DEGs were selected using the threshold of FC > 1.5 and *P* value < 0.1 according to the RNA-seq data. **g** ChIP-seq tracks for H3K36me3 at *SH2B3* gene locus between SETD2_Y1666-targeted A375 mutant cells and *AAVS1*-targeted A375 control cells. **h** IB analysis of SH2B3 and typical JAK/STAT signaling components in A375 cells infected with sgRNAs targeting *AAVS1* and SETD2_Y1666, respectively

The above analyses drew a comprehensive map of regulators for surface PD-L1 and HLA-I at the residue level. However, in the in vivo tumor microenvironment, various factors collectively influence the fate of tumor cells. To gain a deep understanding of the co-regulators of PD-L1 and HLA-I, two of the principal factors for immunotherapy, we conducted a comparison of candidates from the HLA-I and PD-L1 screens in the presence and absence of IFNγ stimulation (Fig. 5a). This analysis identified five mutants that upregulated HLA-I expression while downregulating PD-L1 expression in the presence of IFNγ, including Y137 and Y138 of the N-terminal acetyltransferase NAA20 (Supplementary Fig. 2b; Supplementary Fig. 7a). These mutants were validated to significantly sensitize A375 cells to CD8 + T cell-mediated cytotoxicity, suggesting their potential roles as positive regulators of antitumor immunity (Supplementary Fig. 7i). Conversely, one mutant, MAPK3_Y333, was found to downregulate HLA-I expression and upregulate PD-L1 expression and render A375 cells to be more resistant to T cell killing (Fig. 2c; Fig. 4d; Supplementary Fig. 7i), indicating its potential role in promoting tumor evasion. Additionally, we identified 13 mutants that concurrently upregulated HLA-I and PD-L1 expressions, including six hits that increased PD-L1 levels upon IFNγ treatment, such as EZH2_Y153, EED_Y308, and SETD2_Y1666, all of which are involved in epigenetic modulations.

To explore the regulatory mechanisms of these novel co-regulators, we focused on the functional investigation of the category with the largest number of mutants, which increased the expression level of both PD-L1 and HLA-I. Among them, SETD2_Y1666, as well as the corresponding coding gene, stood out as a novel regulator, whose relevance with PD-L1 or HLA-I has not yet been reported. SETD2 is the primary histone methyltransferase responsible for catalyzing H3K36me3, representing a marker of transcriptional activation. SETD2 is associated with diverse biological functions, such as maintenance of genomic stability,^45^ antiviral immune response,^46^ and restriction of tumor metastasis.^47^ SETD2 mutations are prevalent in various human tumors and are reported to be associated with tumor progression, including glioma, clear cell renal cell carcinoma, leukemia, and prostate cancer.^48–51^ We then investigated the impact of SETD2 gene knockout and observed that its disruption significantly upregulates both the cell surface and total PD-L1 and HLA-I expression levels with IFNγ treatment (Supplementary Fig. 8a, b).

For the SETD2_Y1666 mutation, Y1666 is in the SET domain of SETD2, which is the catalytic domain mediating H3K36me3-specific methyltransferase activity.^52^ SETD2_Y1666 targeted by ABEmax could generate the Y1666C mutation, a reported mutation from both COSMIC (Catalogue of Somatic Mutations in Cancer) and ICGC database (Fig. 5b). We found that Y1666C didn’t change the expression of SETD2 at both the mRNA and protein levels, but it significantly increased the total and membrane protein levels of PD-L1 and HLA-I upon IFNγ exposure (Fig. 5c, d). Meanwhile, the expression level of H3K36me3 was markedly decreased, suggesting that the Y1666C mutation disrupted the catalytic activity of SETD2 without affecting its own protein expression (Fig. 5d).

To elucidate the mechanisms of PD-L1 and HLA-I regulation by SETD2_Y1666, we performed RNA-seq and H3K27me3 ChIP-seq analysis for SETD2_Y1666C mutant cells and control cells with IFNγ stimulation, gaining insight into the potential targets of SETD2. We analyzed the differential expressed genes (DEGs) from the RNA-seq data and identified numerous representative upregulated DEGs in the mutant cells, as exemplified by *CD274*, *IRF1*, *TAP1*, *B2M*, *HLA-A*, *HLA-B* and *HLA-C*, all of which are directly associated with PD-L1 and HLA-I expressions (Fig. 5e). By analyzing the enriched KEGG pathways of upregulated genes, we found dominant terms, including cytokine-cytokine receptor interactions, transcriptional misregulation in cancer, NF-κB signaling pathway, JAK-STAT signaling pathway, and antigen processing and presentation (Fig. 5f). We further referred to the ChIP-seq data to search for the methylated targets of SETD2 and found genes with a significant reduction in H3K36me3 signal, such as *RCC1* (Supplementary Fig. 8c), which was reported to enhance PD-L1 expression and improve the ICB sensitivity after gene knockdown.^53^ *RCC1* was also downregulated in the RNA-seq analysis (Fig. 5e), indicating that SETD2_Y1666 mutation could decrease the H3K36me3 modification of *RCC1*, thus upregulating PD-L1 expression. Interestingly, multiple gene body regions of *SH2B3* exhibited a remarkable lower H3K36me3 signal (Fig. 5g), and SH2B3 appeared to be downregulated upon SETD2_Y1666 mutation (Fig. 5e). Considering the negative regulation of SH2B3 on the JAK-STAT signaling pathway (Fig. 3f, g) and the detected enrichment of JAK-STAT signaling in SETD2_Y1666C mutant cells (Fig. 5f), we further investigated the effects on this pathway when Y1666 was mutated. We found that SETD2_Y1666C conferred a significant reduction in SH2B3 expression, along with higher expression of JAK2 and pSTAT1 (Fig. 5h; Supplementary Fig. 8d), which correlated with the effects of SH2B3 mutants. We also detected upregulation of IFNγ responsive genes such as *IRF1*, some interferon stimulated genes including *ISG15*, *ISG20*, and *MX1* (Fig. 5e, Supplementary Fig. 8d), which are associated with the upregulation of PD-L1 and HLA-I.^54,55^ The above analysis revealed that the Y1666 mutation in the SET domain of SETD2 could boost JAK-STAT signaling pathway, thus increasing PD-L1 expression and antigen processing and presentation.

### Functional clinical mutations promote cancer immunotherapy in different tumor models

**Fig. 6:**
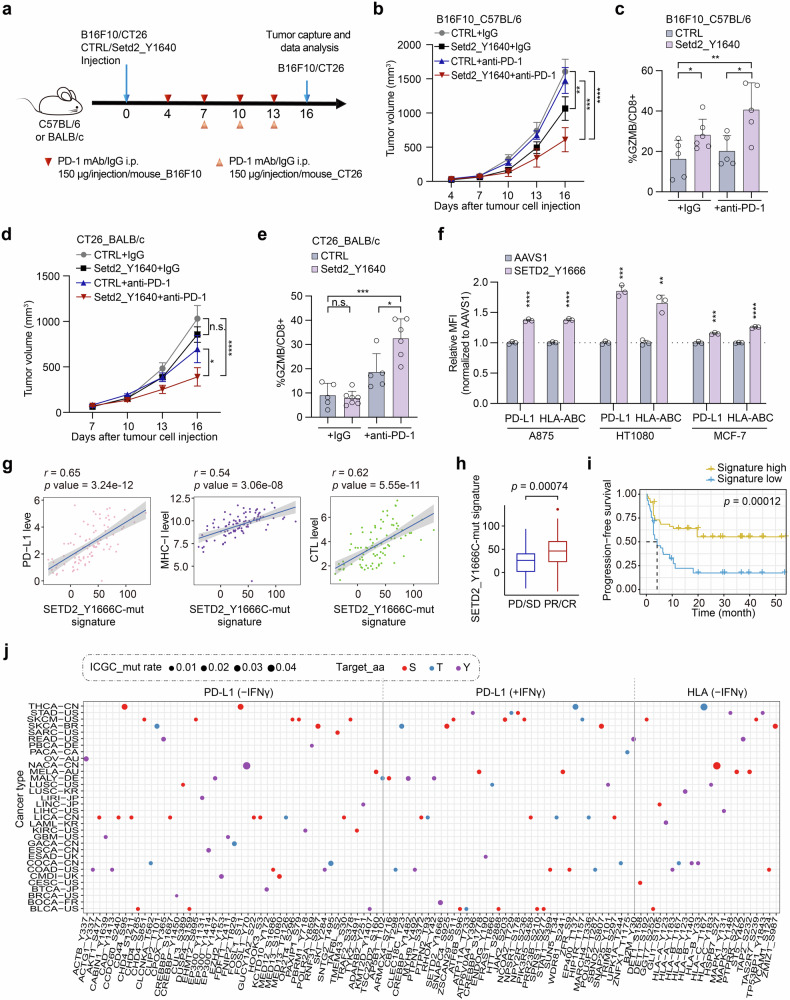
Clinically relevant mutation SETD2_Y1666/Setd2_Y1640 contributes to an improved response to ICB therapy in different tumor models. **a** A schematic view of implanting B16F10 mutant cells and control cells into C57BL/6 mice and the following treatment of PD-1 mAb or IgG isotype control (IgG2a). **b** Longitudinal tumor size of the indicated B16F10 tumors in C57BL/6 mice treated by control IgG or ICB. **c** Quantification of GzmB represented as percentages on CD8+ TILs in B16F10 tumors harvested from C57BL/6 mice after the indicated treatments. **d** Longitudinal tumor size of the indicated CT26 tumors in BALB/c mice treated by control IgG or ICB. **e** Quantification of GzmB represented as percentages on CD8+ TILs in CT26 tumors harvested from BALB/c mice after the indicated treatments. **f** Assessment of the impact of representative regulators on cell surface PD-L1 or HLA-I expression in A875, HT1080 and MCF-7 cells by flow cytometry analysis. Cell surface PD-L1 was analyzed following incubation with 100 ng/mL IFNγ for 48 h. Correlation between the SETD2_Y1666-mutation signature and PD-L1 expression, MHC-I expression, intratumoral CTL infiltration (**g**), ICB response (**h**), and overall survival and progression-free survival (**i**) in patients treated with anti-PD-1 in the Gide et al. study^56^ in melanoma. *r*: Pearson correlation coefficient, PD: progressive disease, SD: stable disease, PR: partial response; CR: complete response. *P* value was respectively calculated by two-tailed Student’s *t* test (**g**) and log-rank test (**h**). **j** Schematic of representative residues identified in PD-L1 and HLA-I screens with clinical relevance according to ICGC database. X axis indicates functional residues regulating PD-L1 or HLA-I from the ABE screens. Y axis indicates different cancer types defined in ICGC database. The dot size represents the detected missense mutation rate of each indicated residue. For (**b**, **d**), data are presented as the mean ± S.E.M. (n = 5–6 mice/group) for each group at each time point. *P* values were calculated using Two-way ANOVA with Benjamini-Hochberg adjustment for multiple testing, *P < 0.05, **P < 0.01, ****P < 0.0001; n.s., not significant. For (**c**, **e**), data are presented as the mean ± SD (n = 5–6 mice/group) for each group at each time point. *P* values were calculated using two-tailed Student’s *t* test, *P < 0.05, **P < 0.01, ****P < 0.0001; n.s., not significant

Considering the regulatory impact of these clinically relevant mutations on both PD-L1 and HLA-I, we intended to dissect their potential effects on tumor progenesis and response to ICB treatment in vivo. Therefore, we generated the homogenous Setd2_Y1640 mutation in the B16F10 mouse melanoma cell line using the ABEmax system, corresponding to the human SETD2_Y1666C mutation. The sgRNA targeting Setd2_Y1640 were infected into B16F10-ABEmax cell line, which resulted in a similar editing pattern and consistent phenotype as observed in A375 cells (Supplementary Fig. 9a, b). Subsequently, we separately injected the Setd2_Y1640-targeted B16F10 cells into the immune-competent C57BL/6 mice, as well as negative control samples infected with an sgRNA targeting the safe-harbor locus, to establish B16F10 melanoma tumors (Fig. 6a). We observed a reduction in tumor growth in Setd2_Y1640-targeted mice, and the combination of anti-PD-1 treatment further inhibited tumor progression (Fig. 6b). Meanwhile, we observed a consistent tumor growth pattern between the mutant group and the control in the immune-deficient BALB/C nude mice (Supplementary Fig. 9c), indicating that the mutation contributes to tumor suppression only through reshaping the immune microenvironment.

To further investigate the impact of Setd2_Y1640 mutation and its combination with ICB treatment on TME, we analyzed infiltrated immune cells in B16F10 tumor-bearing C57BL/6 mice. In Setd2_Y1640 mutant group, we observed increased expression of the T cell activation marker Granzyme B (GzmB) in infiltrated CD8 + T cells compared to the control, and the combination of ICB treatment further strikingly elevated the ratio (Fig. 6c). These results indicated that the mutation might reshape the TME through the activation of representative signaling pathways, including NF-*κ*B and JAK-STAT, which in turn upregulated PD-L1 and HLA-I expression. This enhances the cytotoxicity of tumor infiltrating CD8 + T cells and improves the efficacy of anti-PD-1/PD-L1 blockade therapy in vivo.

We also validated the Setd2_Y1640 mutation in an additional colorectal cancer mouse model, the CT26 tumor-bearing model. CT26 mutant cells and control cells were respectively implanted into immunocompetent BALB/c mice, followed by anti-PD-1 or IgG treatment at the specified time point. Consistent with results from the B16F10 tumor model, the combination of anti-PD-1 treatment significantly inhibited tumor progression in the Setd2_Y1640 mutant group. Additionally, elevated GzmB expression levels were also observed in infiltrated CD8 + T cells in the ICB combination group compared to the control (Fig. 6d, e). Furthermore, we investigated the effects of the SETD2_Y1666 mutation in other human cell lines and found that it exhibited consistent phenotypes in A875, HT1080 and MCF-7 cell lines, further demonstrating its broad applicability across different tumor backgrounds (Fig. 6f).

After confirming the effects on immune response in the mouse model, we next attempted to analyze the correlation between genetic mutation-derived functional deficiency and the response to immunotherapy in published ICB treatment cohorts. We first derived the gene expression signature of the SETD2_Y1666C mutation based on its RNA-seq results, as described in a previous study.^11^ Referring to 91 RNA-seq samples from 54 patients in a melanoma cohort treated with anti-PD-1,^56^ we confirmed that the SETD2_Y1666C-mutation signature was positively correlated with tumor PD-L1, MHC-I, and cytotoxic T-cell infiltration (Fig. 6g). Further analysis revealed that patients responding to ICB therapy (partial response and complete response: PR/CR) exhibited higher SETD2_Y1666C-mutation signature compared with non-response groups (progressive disease and stable disease: PD/SD) (Fig. 6h), and the mutation signature also showed a positive correlation with progression-free survival (Fig. 6i). Interestingly, recent studies also found that patients with different cancer types that harboring SETD2 deleterious mutations showed improved response to ICB therapy.^57,58^ Collectively, these findings firstly demonstrated the mechanisms of SETD2_Y1666C mutation in modulating immune surveillance and further supported the notion that the mutation is relevant to a better response to ICB treatment in clinical trials. Due to the high mutation rate of SETD2 in various cancer types, SETD2 may serve as a biomarker for ICB treatment, and a large population of patients may benefit from immunotherapy.

In addition to the clinical mutations SETD2_Y1666C described above, we sought to investigate the clinical relevance of all selected mutants identified in the screens. Referring to different sequencing data from cancer patients, including ICGC and COSMIC, we found 168 sites with detected mutations across 35 tumor types in ICGC (Fig. 6j; Supplementary Fig. 10a), and more than 300 sites recorded in COSMIC (Supplementary Fig. 10b–d). Overall, nearly 40% (416/1083) of the identified residues from the three screens were clinically observed in these databases, providing a rich resource of potential pathogenic mutations, especially those linked to cancer. Furthermore, this information offers guidance on the efficacy of ICB for patients harboring these mutations.

## Discussion

In this report, we conducted multiple large-scale sgRNA library screens using the ABE system to identify functional genetic variants that modulate the expression of two crucial determinants in cancer immune response: PD-L1 and HLA-I. These factors play a pivotal role in the effectiveness of ICB therapy, particularly in the context of PD-1/PD-L1 blockade. We employed a specialized library targeting 584,377 sites across the genome, encompassing all designable residues of serine, threonine, and tyrosine. Through this approach, we successfully identified over 1,000 novel sites associated with the upregulation or downregulation of PD-L1 or HLA-I expression, using stringent criteria. These identified residues are enriched in several critical immune-related pathways, such as chromatin remodeling, histone modification, JAK/STAT signaling, and antigen processing and presentation. We also confirmed that representative residues exhibit functional roles across multiple cancer cell lines. This comprehensive mapping provides valuable insights into the regulation of cancer immune responses at both the amino acid and base levels for the first time.

To systematically identify critical sites involved in immune response regulation, we initiated our investigation by focusing on one of the key PTMs, phosphorylation. Phosphorylation is known to play a crucial role in signaling transduction and the regulation of gene expression. Our screens identified numerous residues on well-known genes as well as novel genes associated with IFNγ-induced JAK/STAT signaling, including IFNγ receptors, JAK kinases, STATs, and proteins from SH2-B family and PTP family. Among these sites, there was a significant enrichment of well-known phosphorylation sites, including STAT1_Y106 and Y701, JAK1_Y806 and Y830, and JAK2_Y1007 and Y1008. Additionally, we uncovered several predicted phosphorylation sites, exemplified by multiple sequential sites that were concentrated within the SH2 domain of STAT1 and SH2B3. Mutations at these phosphorylation sites have the potential to deactivate the target genes and result in the disruption of phosphorylation events, ultimately leading to the downregulation of PD-L1 or HLA-I.

Importantly, our screens were not limited to investigating phosphorylation sites. Amino acid substitutions can lead to either decreased, increased, or unchanged protein levels, resulting in gene inactivation or augmentation. Thus, different from canonical CRISPR/Cas9 screens, which primarily focus on gene-level dysfunction, base editing-based screens allow for both LOF and GOF perturbations in a single screen. For instance, we found that for the positive regulators of JAK/STAT signaling, such as STAT1 and STAT3, our screens identified mutations that either downregulated or upregulated gene expressions. We found that the majority mutations downregulate gene expressions, which may affect mRNA or protein stability, including known phosphorylation sites such as STAT1_Y106 and Y701. Additionally, a certain number of mutations do not alter the expression levels of the targeted genes but impact their functional properties. For example, STAT1_S462 affects DNA binding capacity, mutations in SH2-B adaptor proteins and CBL disrupt protein-protein interactions, and SETD2_Y1666 impairs enzymatic catalytic activity. These functional sites unveiled novel and comprehensive mechanisms of cancer immune response regulation, which cannot be fully explored through gene-level screens alone.

While previous studies have investigated the regulation of PD-L1 or HLA-I through separate CRISPR screens, the coordinated regulation of both PD-L1 and HLA-I has not been systematically explored, especially at the residue level. The influence of co-regulators on the immune response is complex and relatively unpredictable, prompting us to investigate their roles in immunosurveillance and their impact on anti-PD-1 ICB therapy. In this report, based on functional residue screens for PD-L1 and HLA-I regulation, we identified numerous sites that specifically modulate each factor. Furthermore, we highlighted novel residues that simultaneously modulate PD-L1 and HLA-I expression and delved into their in vivo functions. Among them, we focused on the SETD2_Y1666 mutation, which upregulated both PD-L1 and HLA-I. Notably, the role of its corresponding gene in PD-L1 or HLA-I regulation has not been previously reported. We discovered that this mutation significantly impaired the catalytic activity of the enzyme, without affecting its protein expression levels. Intriguingly, it promoted immune responses and enhanced the efficacy of anti-PD-1 immunotherapy in two different mouse models. For SETD2_Y1666, the upregulation of HLA-I-dependent antigen presentation appeared to counterbalance the adverse effect of PD-L1-mediated immune evasion, reshaping the tumor immune microenvironment to favor anti-PD-1/PD-L1 immunotherapy. SETD2-dependent PD-L1 induction could further enhance the effectiveness of anti-PD-1 blockade. The interplay between HLA-I and PD-L1 expression levels will likely determine the overall therapeutic efficacy of PD-1 inhibitors. In support of our view, previous studies also reported that deficiencies in negative regulators of PD-L1, such as ADORA1,^59^ UROD,^8^ and USP8 (a negative regulator of both PD-L1 and HLA-I),^60^ can enhance the therapeutic effects of anti-PD-1/PD-L1 immunotherapy in vivo. Future research using humanized mouse model will further elucidate the functionality of these variants.

Consistence with a previous base editing screen for IFNγ signaling regulators,^17^ many of the functional mutations identified in our screens have clinical precedence, as supported by data from the ICGC and COSMIC databases. This suggests the prevalence of cancer immunoediting and highlights the clinical significance of these mutations. For both clinically characterized and uncharacterized mutations, our multidimensional screens unveiled their potential impacts on cancer development and progression. Additionally, our dataset provides clinically relevant biomarkers for predicting immune response and resistance to ICB treatment, while also suggesting novel strategies for combinational immunotherapy. Moreover, multiple CRISPR/Cas9 screens have identified a series of PD-L1 or MHC-I regulators that can serve as druggable targets, such as CMTM6,^61,62^ EZH2,^9,10^ TRAF3,^11^ highlighting the importance of combination therapy with ICB. The base-level screens presented in this study not only revealed the importance of single residues but also identified several novel genes, including *FECH*, *TAF5L*, *TAF6L*, *CHMP5*, *NAA20*, and *SETD2*, further enriching the resource of potential therapeutic targets for combination ICB therapy. Importantly, the base-level information provides mechanistic insights that can guide the development of novel drugs.

To sum up, our study provides a comprehensive resource of functional residues involved in the regulation of PD-L1 and HLA-I, shedding light on the understanding of human immune responses at the base level. This initial step in mapping the regulatory residues involved in immunosurveillance can be further complemented by investigating other PTMs, such as ubiquitylation, and by employing other gene editing tools, including prime editors^63–65^ or PAMless Cas9-based base editors,^66^ to further expand the coverage of amino acids.

## Materials and methods

### Mice

Female C57BL/6 mice, BALB/c mice, and BALB/c nude mice (6 to 8 weeks old) were purchased from Beijing Vital River Laboratory Animal Technology Co., Ltd. All mice were bred and kept under specific pathogen-free (SPF) conditions in the Laboratory Animal Center of Peking University. The animal experiments were approved by Peking University Laboratory Animal Center (Beijing) and conducted in accordance with the National Institute of Health Guide for Care and Use of Laboratory Animals.

### Cell lines

The HEK293T cell line was obtained from EdiGene Inc., and the A375, B16F10, HT1080, and MCF-7 cell lines were purchased from ATCC. The CT26 cell line was from Dr. Zhihua Liu’s lab (Chinese Academy of Medical Sciences and Peking Union Medical College) and the A875 cell line was from Dr. Yang Wang’s lab (Peking University First Hospital). The A375-Cas9, A375-ABEmax, A875-ABEmax, HT1080-ABEmax, MCF-7-ABEmax, B16F10-ABEmax, and CT26-ABEmax cell lines were generated in this study. HEK293T, A375, A875, HT1080, MCF-7 and B16F10 cells were cultured in Dulbecco’s modified Eagle’s medium (DMEM, Gibco, #C11965500BT) containing 10% fetal bovine serum (FBS, Biological Industries, #04-010-1A) and 1% penicillin/streptomycin (P/S). CT26 cells were cultured in RPMI1640 medium (Gibco, #C11875500BT) supplemented with 10% FBS and 1% P/S. All cells were cultured with 5% CO_2_ at 37 °C and were routinely checked to confirm the absence of mycoplasma contamination using Mycoplasma Detection Kit (InvivoGen, #rep-mys-50).

### Primary human T cells

Peripheral blood mononuclear cells (PBMCs) were obtained from healthy donors, all of whom provided informed consent. The collection of PBMCs was conducted in accordance with a protocol approved by the Ethics Committee of Shenzhen Hospital of University of Chinese Academy of Sciences (ID: LL-KT-2022055). Primary human T cells expressing the anti-NY-ESO-1 TCR were generated by retroviral transduction according to previous studies described,^10,67^ and were frozen in the cryopreservation medium (Stemcell Technologies, #100-1061). Once thawed, T cells were maintained in T cell expansion medium (Stemcell Technologies, #10981) supplemented with 10% FBS, 1% penicillin/streptomycin, and 50 ng/mL IL-2 (Stemcell Technologies, #78036.3). T cells were activated and expanded using human CD3/CD28 T cell activator (Stemcell Technologies, #10971) for 3 days, and then subjected to subsequent experiments.

### Plasmids

pLenti-ABEmax-P2A-EGFP expression plasmid was constructed by cloning ABEmax_P2A_EGFP sequence from pCMV_ABEmax_P2A_GFP (Addgene, #112101) into the lentiviral vector. All sgRNAs used for validation (Supplementary Table 7) were cloned into the pLenti-sgRNA(lib)-puro vector (Addgene, #119976) through Golden Gate assembly. Protein-coding sequences for cDNA over-expression or co-immunoprecipitation were cloned into pLenti_CMV_cDNA_Flag_SV40_mCherry vector or pLenti_CMV_cDNA_HA_SV40_EGFP vector by PCR and Gibson assembly (NEB, #E2611L). All plasmids were verified by Sanger sequencing.

### ABE screens for functional S/T/Y residues in A375 cells

The A375-ABEmax cells were seeded in 15 cm dishes 24 h before lentivirus infection, then were respectively transduced with each of the S/T/Y lentiviral libraries (sense library and antisense library) at an MOI of 3 with a high coverage for each sgRNA (about 1,500-fold, about 500-fold for each iBAR). Forty-eight hours post transduction, the library cells were cultured with 1 μg/mL puromycin (Solaribio, #P8230) for two days. After puromycin selection, the time point was denoted as Day 0 of the screening, and the library cells with at least 1,500-fold coverage for sgRNAs were maintained and passaged every 3 days. At Day 10 (IFNγ-absent screens) or Day 13 (IFNγ-treated screens), PD-L1^high/low^ and HLA^high/low^ cells were respectively subjected to the first round of FACS enrichment by BD FACS Aria III or MoFlo Astrios EQ (Beckman). For the PD-L1 screens, cells were pre-treated with or without 100 ng/mL IFNγ (Sino Biological, #GMP-11725-HNAS) for 48 h and stained with APC anti-human CD274 antibody (BioLegend, #329708) before FACS. For the HLA screens, cells were stained with APC anti-human HLA-A,B,C antibody (BioLegend, #311410) before FACS. At least three times of the library cells were subjected to immunofluorescence staining, and 1 μL antibody per million cells in 100 μL staining buffer (BioLegend, #420201) were used in the staining according to the standard protocol. In each group, the highest and lowest 10% of cells were collected based on APC fluorescence. One week after the first-round sorting, the cells were stained with the same antibodies and were further subjected to FACS enrichment. In the second-round sorting, APC-positive or APC-negative library cells were collected for each group through comparing with A375 cells infected with *AAVS1*-targeted control sgRNA (Supplementary Fig. 1e–h). At Day 24, the library cells without FACS were havested as the reference group and the FACS-enriched cells from the second-round sorting of each group were havested as the experimental groups.

### Genomic DNA isolation and amplicon sequencing of the S/T/Y library

Genomic DNA was extracted from reference cells and experimental cells using the DNeasy Blood & Tissue Kit (Qiagen, #69506). For each group, all extracted genomes were used as the PCR templates and the sgRNA-coding sequences with iBAR were amplified using KAPA HiFi HotStart ReadyMix PCR kit (Roche, #KK2631). The DNA amplification was performed under the following condition: 30 s at 95 °C for initial denaturation; 26 cycles consisting of 10 s at 95 °C for denaturation, 30 s at 60 °C for annealing, and 15 s at 72 °C for extension; and 15 s at 72 °C for final extension. The PCR products of each group were pooled and purified using DNA Clean & Concentrator-25 kit (Zymo Research, #D4034), followed by next-generation sequencing (NGS) analysis on Illumina HiSeq X TEN platform.

### Computational analysis of screens

To analyze the NGS data of the screens, we used MAGeCK-iBAR algorithm^24^ to evaluate the change of sgRNA abundance between the reference group and each experimental group. We used default parameters of MAGeCK-iBAR to calculate the *P* value (lo_value in the output) for each sgRNA considering both the significance and consistency of three iBARs. The final screen score was defined as −log_10_ of *P* value after Benjamini-Hochberg (BH) adjustment and sgRNAs with a screen score of more than 1 were selected as the negatively or positively enriched candidates for follow-up studies.

### Validation of candidate sites identified in the screens

A375-Cas9, A375-ABEmax, A875-ABEmax, HT1080-ABEmax, MCF-7-ABEmax, B16F10-ABEmax, or CT26-ABEmax cells were transduced with lentivirus of each sgRNA targeting candidate site or *AAVS1* at an MOI of <1, and the time point of lentivirus infection was denoted as T0. Forty-eight hours post transduction (T2), cells were treated with 1 μg/mL puromycin for two days and the resistant cells were passaged for two generations. For the IFNγ-absent condition, sgRNA-infected cells were collected at T9. For the IFNγ-treated condition, sgRNA-infected cells were seeded at T8, treated with 100 ng/mL IFNγ (for A375, A875, HT1080, and MCF-7) or 10 ng/mL IFNγ (for B16F10) at T9 for 48 h, and finally collected at T11. For both conditions, sgRNA-infected cells cultured in 6-well plates were washed by DPBS (Gibco, #C14190500BT), followed by detachment using accutase (BioLegend, #423201). One million cells were collected and resuspended in 100 μL staining buffer with 1 μL anti-human CD274 antibody, anti-human HLA-A,B,C antibody or anti-mouse H2-K^b^ antibody following the standard protocol. Flow cytometry analysis was performed with the BD LSRFortessa SORP (BD Biosciences). Changes in PD-L1 or MHC-I surface expression were calculated as the changes in raw median fluorescence intensity (MFI). The relative MFI of all samples was normalized to the isotype control or further normalized to the *AAVS1*-targeted control cells. Antibodies used in the validation include anti-human CD274 antibody (APC, clone 29E.2A3, BioLegend, #329708), anti-human HLA-A,B,C antibody (APC, clone W6/32, BioLegend, #311410), anti-mouse H2-K^b^ antibody (APC, clone AF6-88.5, BioLegend, #116518), Mouse IgG2b, *κ* Isotype Ctrl (APC, clone MPC-11, BioLegend, #982108), Mouse IgG2a, *κ* Isotype Ctrl (APC, clone MOPC-173.

### Detection of base editing outcomes by NGS

A375-ABEmax or B16F10-ABEmax cells were transduced with lentivirus of each sgRNA targeting candidate site at an MOI of >1 and were further treated with puromycin as described above. Seven days post transduction, sgRNA-infected cells were collected and subjected to genome DNA isolation. For the mutant cells and WT cells, about 200-bp genomic sequences surrounding each sgRNA-targeted site were amplified using specific primers by PrimeSTAR® GXL Premix (TAKARA, #R051A), followed by NGS analysis on Illumina HiSeq X TEN platform. The paired-end NGS data was first assembled by PANDAseq software. The sequence of sgRNA-targeted regions was extracted from the assembled fasta files by their flanking sequence, which was 10 bp upstream and 10 bp downstream of the sgRNA-targeted regions. The percentage of A/T/C/G in each position was further calculated, including the targeted site and sgRNA editing window, to assess on-target editing efficiency as well as bystander editing for each candidate sgRNA.

### Real-time qPCR analysis

For the IFNγ-absent or IFNγ-treated condition, A375-ABEmax cells infected with each indicated sgRNA were respectively collected at T9 or T11 as described above. RNA of the sgRNA-infected cells was extracted using RNAprep pure Cell/Bacteria Kit (TIANGEN, #DP430), and the cDNA was synthesized using HifairII 1st Strand cDNA Synthesis SuperMix (YEASEN, #11120ES60). Real-time qPCR was performed using TB Green Premix Ex Taq II (TaKaRa, #RR820A) on Roche LightCycler480 Real-Time PCR System. All cDNA samples were assayed in triplicate and the relative RNA expression level of each sample was normalized by *GAPDH*. All the primers used for real-time qPCR are listed in Supplementary Table 8.

### Immunoblotting

A375-ABEmax or A375-Cas9 cells infected with each indicated sgRNA were inoculated in 6-well plates and were respectively collected at T9 or T11 for different IFNγ treatments as described above. Cells were washed twice with PBS and were lysed using pre-cooled RIPA lysis buffer supplemented with protease and phosphatase inhibitor (Thermo Fisher Scientific, #78441) on ice for 30 min. After quantifying the protein concentration by the BCA method (Thermo Fisher Scientific, #23225), the lysates were electrophoretically separated by 12% SDS-PAGE gel and transferred to a PVDF membrane (Bio-Rad, #10026934). The proteins were blocked with 5% skim milk (Thermo Fisher Scientific, #232100) in PBST or TBST at room temperature for 1 h and were further incubated with the primary antibody at 4 °C overnight. The PVDF membranes were washed with PBST or TBST three times and then incubated with HRP secondary antibodies (1:10000) at room temperature for 1 h. The secondary antibodies include: goat anti-rabbit IgG-HRP (Jackson Immunoresearch, #111035003) or goat anti-mouse IgG-HRP secondary antibody (Jackson Immunoresearch, #115035003). After being washed with TBST three times, the protein bands were detected by using Clarity^TM^ Western ECL Substrate Kit (Bio-Rad, #1705060) on the Chemidoc^TM^ system (Bio-Rad, #1708370).

### Immunoprecipitation

8 × 10^5^ HEK293T cells were seeded in 6-well plates for each sample. The cells were transfected with indicated plasmids on the second day, followed by stimulation with 100 ng/mL IFNγ on the third day for 48 h. Cells were washed in PBS, lysed in RIPA lysis buffer with protease and phosphatase inhibitor on ice for 30 min, and further pelleted by centrifugation at 12,000 × *g* for at 4 °C for 10 min. The supernatant was collected with 30 µL of the cell lysates as the input, and the rest was treated with Anti-Flag M2 Affinity Gel (Sigma-Aldrich, #A2220) or HA beads (Sigma-Aldrich, #E6779) at 4 °C overnight. After washing the lysates four times with RIPA buffer, 5× loading buffer was added to the sample, followed by boiling at 100 °C for 10 min. Then the immunoblotting analysis was carried out as described above. Antibodies used for immunoblotting include: rabbit polyclonal anti-HA (Sigma-Aldrich, #H6908/SAB4300603, 1:10000), rabbit polyclonal anti-FLAG (Sigma-Aldrich, #F7425, 1:10,000) and mouse monoclonal anti-FLAG (Sigma-Aldrich, #F1804, 1:10,000).

### Detection of sialic acid by flow cytometry

A375-ABEmax cells infected with sgRNA targeting SLC35A1_Y98 and *AAVS1* were collected at the 9th day post lentivirus infection. After DPBS washing and accutase detachment, one million cells were washed by DPBS twice, resuspended in 1 mL PBS supplemented with 0.5% BSA. The Maackia Amurensis Lectin II (MAL-II)-biotin (Vector Laboratories, #B-1265) was added to the suspension at a final concentration of 5 µg/mL, followed by incubation at room temperature for 30 min. Next, the cells were washed with DPBS three times and stained with 1 µg/mL Streptavidin-Alexa Fluor 647 (AF647) (BioLegend, #405237) for another 30 min. After washing with DPBS three times, flow cytometry analysis was performed to detect the AF647 (APC) signal with the BD LSRFortessa SORP (BD Biosciences). Changes in sialic acid surface expression were calculated as the changes in raw MFI, and the relative MFI was generated by normalization to the fluresence of unstained cells.

### Competitive T cell killing assay

A375 cells, which endogenously express NY-ESO-1 antigen, were further engineered to stably overexpress ABEmax with an EGFP marker in this study. In the co-culture experiment, A375-ABEmax cells infected with each indicated sgRNA were first mixed with A375 WT cells in a 1:1 ratio, then were seeded in 48-well plates and allowed to attach for 12 h before adding the anti-NY-ESO-1 TCR-transduced primary human T cells at an appropriate effector to target cell (E:T) ratio. Meanwhile, paired controls without adding T cells were included for each condition. After co-culturing the targeted A375 cells and T cells in RPMI 1640 medium (Gibco, #11875093) for 6 h, the cells were washed twice by DPBS to remove most of the surface T cells. Then the A375 cells along with some adherent T cells were detached with accutase, followed by staining with anti-human CD3 (BV650, clone UCHT1, BioLegend, #300467) and DAPI (BioLegend, #422801) to further exclude T cells and dead cells. Flow cytometry analysis was performed with the BD LSRFortessa SORP (BD Biosciences), and the percentage of EGFP+ cells was measured after gating out T cells and dead cells. The extent of the killing sensitivity was defined as: 100 × (1-(A1/100-A1)/(B1/100-B1)), A1: Percentage of A375-ABEmax cells (represented as EGFP+ cells) that were incubated with T cells, B1: Percentage of A375-ABEmax cells that were not incubated with T cells. The extent of the killing resistance was defined as: 100 × (1-(A2/100-A2)/(B2/100-B2)), A2: Percentage of A375 WT cells that were incubated with T cells, B2: Percentage of A375 WT cells that were not incubated with T cells.^68^ For each sample, both of the co-culture assay and the paired control were performed in triplicate.

### RNA-seq and data analysis

The sgRNA targeting SETD2_Y1666 or *AAVS1* was individually transduced into A375-ABEmax cells at an MOI of <1 in duplicate or triplicate. At T11 as described above, 2 × 10^6^ cells were collected after IFNγ treatment for two days. The total RNA of each sample was extracted using the RNeasy Mini Kit (QIAGEN, #79254), and the RNA-seq libraries were prepared as previously described.^69^ All samples were subjected to NGS analysis using the Illumina HiSeq X TEN platform. The RNA sequencing data was first processed by FASTP software to cut adapters and filter low quality sequences. Then HISAT2 was used to map the reads to human reference genome hg38 under default parameters. The raw counts of mapped reads for each gene were calculated using featurecounts software. The annotation file for this step was from GENCODE v38 gtf file and the reads in exon level (-t parameter) were counted. The differential gene expression analysis was performed by DESeq2 package (V1.40.2) and the downstream GO enrichment was performed by clusterProfiler package (V3.10.1).

### Chromatin immunoprecipitation with sequencing (ChIP-seq) and data analysis

The ChIP assays were performed using Hyperactive Universal CUT&Tag Assay Kit for Illumina (Vazyme, #TD903). The procedure was according to manufacturer’s instructions. Briefly, sgRNA targeting SETD2_Y1666 or *AAVS1* was individually transduced into A375-ABEmax cells at an MOI of <1 in triplicate, and 50,000 cells were harvested at T11 after IFNγ treatment for two days. Cells were fixed on cleaned NovoNGS CoA beads, followed by incubation with primary anti-H3K36me3 antibody (Abcam, #ab9050) at 4 °C overnight. On the next day, Immunoprecipitates was incubated with Goat anti-Rabbit IgG antibody (1:100) at room temperature for 30 min, and further incubated with protein A/G-Tn5 transposase and ChiTag buffer for 1 h. Next, the samples were subjected to DNA fragmentation by adding tagmentation buffer with incubation at 37 °C for 1 h, followed by DNA extraction through incubation with tagment DNA extract beads, thus obtaining fragmented DNA. Then the ChIP samples were prepared for NGS analysis using VAHTS Universal DNA Library Prep Kit for Illumina v.3 (Vazyme, #ND607) and deep-sequenced on the Illumina HiSeq X TEN platform. The cleaned fastq files was first mapped to human reference genome hg38 using BOWTIE2 under default parameters. Then we used MASC2 to call peaks and chose broad peak pattern considering features of H3K36me3. Different peak analysis was performed by DiffBind package (V2.10.0) in R. Integrative Genomics Viewer (IGV) was used to visualize peaks in the interested regions and the results from three replicates were merged in IGV.

### Mouse experiments

For the immune-competent mouse model, sgRNA targeting Setd2_Y1640 and the negative control sgRNA was individually transduced into B16F10-ABEmax cells or CT26-ABEmax cells, then 2 × 10^5^ sgRNA-infected cells were subcutaneously inoculated into the right flank of 6–8 week-old female C57BL/6 mice or BALB/c mice, which were further divided into control or experimental groups randomly. From Day 7 post transplantation when the tumor volume reached about 100 mm^3^, the control and experimental groups were treated with control IgG (Selleck, #A2123, 150 μg per mouse) or anti-PD-1 (Selleck, #A2122, 150 μg per mouse) by intraperitoneal injection every three days at each indicated time point. For the immunodeficient mouse model, 2 × 10^5^ B16F10 cells infected with each indicated sgRNA were subcutaneously inoculated into the right flank of 6-week-old female BALB/c nude mice. Tumor growth was measured using digital calipers, and tumor sizes were recorded every three days until the sizes reached 2000 mm^3^.

### Isolation of the tumor infiltrated immune cells and flow cytometry analysis

The mouse tumor samples separated from the mice were washed with PBS, then were minced into small pieces and further digested by the RPMI 1640 medium supplemented with 1 mg/mL collagenase D (OKA, #D10032) at 37 °C for 30 min. After terminating the digestion by adding RPMI 1640 medium supplemented with 10% FBS, the solutions were filtered through a 200-mesh cell sieve and centrifuged at 260 × *g* for 4 min. Then the cell pellets were washed by PBS and centrifuged at 260 × *g* for 4 min, thus obtaining single-cell suspensions. Cells were stimulated with anti-CD3/CD28 (3.5 µg/mL anti-CD3 mAb, BioLegend, #100339; 1 µg/mL anti-CD28 mAb, BioLegend, #102115) in the presence of 5 μg/mL Brefeldin A (BFA, Thermo Fisher Scientific, #00-4506-51) and 5 μg/mL monensin (Thermo Fisher Scientific, #00-4505-51), and cultured in a humidified incubator with 95% air/5% CO_2_ at 37 °C incubation for 4 h. Cells were collected by centrifugation at 260 × *g* for 4 min, then washed with 1 mL PBS. After centrifugation at 260 *g* for 4 min to remove the supernatant, the cells were first stained with anti-NK1.1 mAb (PE/Cyanine7, clone PK136, BioLegend, #108713), anti-CD8a mAb (PE, clone 53-6.7, BioLegend, #100708), and Zombie Yellow^TM^ (BioLegend, #423103), then fixed with 2× IC fixation buffer (Thermo Fisher Scientific, #00-8222-49) at room temperature for 15 min in the dark, and treated with 1× permeabilization buffer (Thermo Fisher Scientific, #00-8333-56). After centrifugation at 5,000 *g* for 2 min, the cell pellets were stained with anti-GzmB antibody (FITC, clone QA16A02, BioLegend, #372206), followed by flow cytometry analysis.

### Generation of the SETD2_Y1666-mutation signature

The SETD2-Y1666 mutation signature was defined by extracting top 250 upregulated and top 250 downregulated genes and using the normalized DESeq2 wald statistics as weights, which were calculated on the basis of the equation *k*_*i*_ = *w*_*i*_/*max*(*w*). The *k*_*i*_ stands for the weight of the *i th* gene and *w*_*i*_ indicated the wald statistics of the *i th* gene. Each input expression profiles then could be assessed by computing a SETD2-Y1666 mutation signature score by calculating the sum expression level of the signature genes following the equation *S* = ∑*n*_*i*_ = (*k*_*i*_ * *X*_*i*_), where *S* denotes the signature score and *X*_*i*_ denotes the expression level of the *i* th gene.

### Immunotherapy trials used for correlation analysis

We collected 91 RNA-seq expression profiles from 54 melanoma patients who were treated with anti-PD-1 therapy from published study.^56^ For each RNA-seq sample, the gene expression profile was analyzed following standard pipeline as described above.

### Correlation analysis between the SETD2_Y1666-mutation signature and representative markers

Referred to the melanoma patients’ cohort that we used,^56^ the MHC-I expression levels were calculated as the average log_2_TPM of *HLA-A*, *HLA-B*, *HLA-C*, and *B2M*, the PD-L1 expression levels were calculated as the average log_2_TPM of *CD274*, and the CTL (cytotoxic T lymphocyte) expression levels were calculated as the average log_2_TPM of *CD8A*, *CD8B*, *GzmA*, *GzmB*, and *PRF1*. The Pearson correlations were computed between the SETD2_Y1666-mutation signature and the expression levels of MHC-I, PD-L1, and CTL.

### Survival analysis

The clinical relevance of SETD2_Y1666 in regulating ICB response was confirmed by testing the association between SETD2_Y1666-mutation signature and progressive survival of patients in immunotherapy trials with Cox regression.

### Statistical analyses

Statistical tests, exact value and description of n were presented as described in the figure legends. Unless otherwise noted, n represents biological replicates of the samples (e.g., independent cell cultures, individual tumors, etc.). The statistical significance was evaluated using Student’s *t* test or two-way ANOVA (with BH adjustment for multiple testing), and determined as *P* < 0.05, labeled as *P < 0.05, **P < 0.01, ***P < 0.001, ****P < 0.0001.

## Supplementary information

Supplementary Material

uncropped blots

Table S1

Table S2

Table S3

Table S4

Table S5

Table S6

Table S7

Table S8

## Data Availability

The sequence data have been deposited in the Genome Sequence Archive^70^ in National Genomics Data Center,^71^ China National Center for Bioinformation/Beijing Institute of Genomics, Chinese Academy of Sciences (GSA-Human: HRA005746) that are publicly accessible at https://ngdc.cncb.ac.cn/gsa-human. All data supporting the findings and all materials generated in this manuscript are available upon reasonable request.

## References

[1] Sharma, P., Hu-Lieskovan, S., Wargo, J. A. & Ribas, A. Primary, adaptive, and acquired resistance to cancer immunotherapy. *Cell* **168**, 707–723 (2017). 28187290 10.1016/j.cell.2017.01.017 PMC5391692

[2] Spranger, S. & Gajewski, T. F. Impact of oncogenic pathways on evasion of antitumour immune responses. *Nat. Rev. Cancer* **18**, 139–147 (2018). 29326431 10.1038/nrc.2017.117 PMC6685071

[3] Morad, G., Helmink, B. A., Sharma, P. & Wargo, J. A. Hallmarks of response, resistance, and toxicity to immune checkpoint blockade. *Cell* **184**, 5309–5337 (2021). 34624224 10.1016/j.cell.2021.09.020 PMC8767569

[4] Ribas, A. & Wolchok, J. D. Cancer immunotherapy using checkpoint blockade. *Science* **359**, 1350–1355 (2018). 29567705 10.1126/science.aar4060 PMC7391259

[5] Kalbasi, A. & Ribas, A. Tumour-intrinsic resistance to immune checkpoint blockade. *Nat. Rev. Immunol.* **20**, 25–39 (2020). 31570880 10.1038/s41577-019-0218-4 PMC8499690

[6] Sun, C., Mezzadra, R. & Schumacher, T. N. Regulation and function of the PD-L1 checkpoint. *Immunity* **48**, 434–452 (2018). 29562194 10.1016/j.immuni.2018.03.014 PMC7116507

[7] Mezzadra, R. et al. Identification of CMTM6 and CMTM4 as PD-L1 protein regulators. *Nature* **549**, 106–110 (2017). 28813410 10.1038/nature23669 PMC6333292

[8] Suresh, S. et al. eIF5B drives integrated stress response-dependent translation of PD-L1 in lung cancer. *Nat. Cancer* **1**, 533–545 (2020). 32984844 10.1038/s43018-020-0056-0 PMC7511089

[9] Burr, M. L. et al. An evolutionarily conserved function of polycomb silences the MHC class I antigen presentation pathway and enables immune evasion in cancer. *Cancer Cell* **36**, 385–401.e388 (2019). 31564637 10.1016/j.ccell.2019.08.008 PMC6876280

[10] Dersh, D. et al. Genome-wide screens identify lineage- and tumor-specific genes modulating MHC-I- and MHC-II-restricted immunosurveillance of human lymphomas. *Immunity* **54**, 116–131.e110 (2021). 33271120 10.1016/j.immuni.2020.11.002 PMC7874576

[11] Gu, S. S. et al. Therapeutically increasing MHC-I expression potentiates immune checkpoint blockade. *Cancer Discov.* **11**, 1524–1541 (2021). 33589424 10.1158/2159-8290.CD-20-0812 PMC8543117

[12] Zaretsky, J. M. et al. Mutations associated with acquired resistance to PD-1 blockade in melanoma. *N. Engl. J. Med.* **375**, 819–829 (2016). 27433843 10.1056/NEJMoa1604958 PMC5007206

[13] Shin, D. S. et al. Primary resistance to PD-1 blockade mediated by JAK1/2 mutations. *Cancer Discov.* **7**, 188–201 (2017). 10.1158/2159-8290.CD-16-1223 PMC5296316 27903500

[14] Rooney, M. S., Shukla, S. A., Wu, C. J., Getz, G. & Hacohen, N. Molecular and genetic properties of tumors associated with local immune cytolytic activity. *Cell* **160**, 48–61 (2015). 25594174 10.1016/j.cell.2014.12.033 PMC4856474

[15] Cuella-Martin, R. et al. Functional interrogation of DNA damage response variants with base editing screens. *Cell* **184**, 1081–1097.e1019 (2021). 33606978 10.1016/j.cell.2021.01.041 PMC8018281

[16] Hanna, R. E. et al. Massively parallel assessment of human variants with base editor screens. *Cell* **184**, 1064–1080.e1020 (2021). 33606977 10.1016/j.cell.2021.01.012

[17] Coelho, M. A. et al. Base editing screens map mutations affecting interferon-gamma signaling in cancer. *Cancer Cell* **41**, 288–303.e286 (2023). 36669486 10.1016/j.ccell.2022.12.009 PMC9942875

[18] Kumagai, S. et al. The PD-1 expression balance between effector and regulatory T cells predicts the clinical efficacy of PD-1 blockade therapies. *Nat. Immunol.* **21**, 1346–1358 (2020). 32868929 10.1038/s41590-020-0769-3

[19] Havel, J. J., Chowell, D. & Chan, T. A. The evolving landscape of biomarkers for checkpoint inhibitor immunotherapy. *Nat. Rev. Cancer* **19**, 133–150 (2019). 30755690 10.1038/s41568-019-0116-x PMC6705396

[20] Anderson, P., Aptsiauri, N., Ruiz-Cabello, F. & Garrido, F. HLA class I loss in colorectal cancer: implications for immune escape and immunotherapy. *Cell Mol. Immunol.* **18**, 556–565 (2021). 33473191 10.1038/s41423-021-00634-7 PMC8027055

[21] Montesion, M. et al. Somatic HLA class I loss is a widespread mechanism of immune evasion which refines the use of tumor mutational burden as a biomarker of checkpoint inhibitor response. *Cancer Discov.* **11**, 282–292 (2021). 10.1158/2159-8290.CD-20-0672 33127846

[22] Cha, J. H., Chan, L. C., Li, C. W., Hsu, J. L. & Hung, M. C. Mechanisms controlling PD-L1 expression in cancer. *Mol. Cell* **76**, 359–370 (2019). 31668929 10.1016/j.molcel.2019.09.030 PMC6981282

[23] Humphrey, S. J., James, D. E. & Mann, M. Protein phosphorylation: a major switch mechanism for metabolic regulation. *Trends Endocrinol. Metab.* **26**, 676–687 (2015). 26498855 10.1016/j.tem.2015.09.013

[24] Zhu, S. et al. Guide RNAs with embedded barcodes boost CRISPR-pooled screens. *Genome Biol.* **20**, 20 (2019). 10.1186/s13059-019-1628-0 PMC6345036 30678704

[25] Li, Y. et al. Functional profiling of serine, threonine and tyrosine sites. *Nat. Chem. Biol.*10.1038/s41589-024-01731-0 (2024). 39313591 10.1038/s41589-024-01731-0

[26] Chang, R. et al. Loss of Wwox drives metastasis in triple-negative breast cancer by JAK2/STAT3 axis. *Nat. Commun.* **9**, 3486 (2018). 30154439 10.1038/s41467-018-05852-8 PMC6113304

[27] Wang, G. et al. CRISPR-GEMM pooled mutagenic screening identifies KMT2D as a major modulator of immune checkpoint blockade. *Cancer Discov.* **10**, 1912–1933 (2020). 32887696 10.1158/2159-8290.CD-19-1448 PMC7710536

[28] Qing, Y., Costa-Pereira, A. P., Watling, D. & Stark, G. R. Role of tyrosine 441 of interferon-gamma receptor subunit 1 in SOCS-1-mediated attenuation of STAT1 activation. *J. Biol. Chem.* **280**, 1849–1853 (2005). 15522878 10.1074/jbc.M409863200

[29] Lucet, I. S. et al. The structural basis of Janus kinase 2 inhibition by a potent and specific pan-Janus kinase inhibitor. *Blood* **107**, 176–183 (2006). 16174768 10.1182/blood-2005-06-2413

[30] Quelle, F. W. et al. Phosphorylation and activation of the DNA binding activity of purified Stat1 by the Janus protein-tyrosine kinases and the epidermal growth factor receptor. *J. Biol. Chem.* **270**, 20775–20780 (1995). 7657660 10.1074/jbc.270.35.20775

[31] Ahmed, Z. & Pillay, T. S. Functional effects of APS and SH2-B on insulin receptor signalling. *Biochem. Soc. Trans.* **29**, 529–534 (2001). 11498022 10.1042/bst0290529

[32] Kurzer, J. H., Saharinen, P., Silvennoinen, O. & Carter-Su, C. Binding of SH2-B family members within a potential negative regulatory region maintains JAK2 in an active state. *Mol. Cell Biol.* **26**, 6381–6394 (2006). 16914724 10.1128/MCB.00570-06 PMC1592834

[33] Bersenev, A., Wu, C., Balcerek, J. & Tong, W. Lnk controls mouse hematopoietic stem cell self-renewal and quiescence through direct interactions with JAK2. *J. Clin. Invest.* **118**, 2832–2844 (2008). 18618018 10.1172/JCI35808 PMC2447929

[34] Hu, J. & Hubbard, S. R. Structural characterization of a novel Cbl phosphotyrosine recognition motif in the APS family of adapter proteins. *J. Biol. Chem.* **280**, 18943–18949 (2005). 15737992 10.1074/jbc.M414157200

[35] Gu, F. et al. Protein tyrosine phosphatase 1B attenuates growth hormone-mediated JAK2-STAT signaling. *Mol. Cell Biol.* **23**, 3753–3762 (2003). 12748279 10.1128/MCB.23.11.3753-3762.2003 PMC155228

[36] Kleppe, M. et al. PTPN2 negatively regulates oncogenic JAK1 in T-cell acute lymphoblastic leukemia. *Blood* **117**, 7090–7098 (2011). 21551237 10.1182/blood-2010-10-314286

[37] Jhunjhunwala, S., Hammer, C. & Delamarre, L. Antigen presentation in cancer: insights into tumour immunogenicity and immune evasion. *Nat. Rev. Cancer* **21**, 298–312 (2021). 33750922 10.1038/s41568-021-00339-z

[38] Gettinger, S. et al. Impaired HLA class I antigen processing and presentation as a mechanism of acquired resistance to immune checkpoint inhibitors in lung cancer. *Cancer Discov*. **7**, 1420–1435 (2017). 10.1158/2159-8290.CD-17-0593 PMC5718941 29025772

[39] Chen, X. et al. A membrane-associated MHC-I inhibitory axis for cancer immune evasion. *Cell* **186**, 3903–3920.e3921 (2023). 37557169 10.1016/j.cell.2023.07.016 PMC10961051

[40] Nji, E., Gulati, A., Qureshi, A. A., Coincon, M. & Drew, D. Structural basis for the delivery of activated sialic acid into Golgi for sialyation. *Nat. Struct. Mol. Biol.* **26**, 415–423 (2019). 31133698 10.1038/s41594-019-0225-y

[41] Jongsma, M. L. M. et al. The SPPL3-defined glycosphingolipid repertoire orchestrates HLA class I-mediated immune responses. *Immunity* **54**, 132–150.e139 (2021). 33271119 10.1016/j.immuni.2020.11.003 PMC8722104

[42] Varki, A. et al. *Essentials of Glycobiology, 4th*. (Cold Spring Harbor Laboratory Press, 2022). 35536922

[43] Wearsch, P. A. & Cresswell, P. Selective loading of high-affinity peptides onto major histocompatibility complex class I molecules by the tapasin-ERp57 heterodimer. *Nat. Immunol.* **8**, 873–881 (2007). 17603487 10.1038/ni1485

[44] Robbins, P. F. et al. Single and dual amino acid substitutions in TCR CDRs can enhance antigen-specific T cell functions. *J. Immunol.* **180**, 6116–6131 (2008). 18424733 10.4049/jimmunol.180.9.6116 PMC2424230

[45] Park, I. Y. et al. Dual chromatin and cytoskeletal remodeling by SETD2. *Cell* **166**, 950–962 (2016). 27518565 10.1016/j.cell.2016.07.005 PMC5101839

[46] Chen, K. et al. Methyltransferase SETD2-mediated methylation of STAT1 is critical for interferon antiviral activity. *Cell* **170**, 492–506.e414 (2017). 28753426 10.1016/j.cell.2017.06.042

[47] Yuan, H. et al. SETD2 restricts prostate cancer metastasis by integrating EZH2 and AMPK signaling pathways. *Cancer Cell* **38**, 350–365.e357 (2020). 32619406 10.1016/j.ccell.2020.05.022

[48] Fontebasso, A. M. et al. Mutations in SETD2 and genes affecting histone H3K36 methylation target hemispheric high-grade gliomas. *Acta. Neuropathol.* **125**, 659–669 (2013). 23417712 10.1007/s00401-013-1095-8 PMC3631313

[49] Cancer Genome Atlas Research, N. Comprehensive molecular characterization of clear cell renal cell carcinoma. *Nature* **499**, 43–49 (2013). 23792563 10.1038/nature12222 PMC3771322

[50] Zhu, X. et al. Identification of functional cooperative mutations of SETD2 in human acute leukemia. *Nat. Genet.* **46**, 287–293 (2014). 24509477 10.1038/ng.2894 PMC4440318

[51] Armenia, J. et al. The long tail of oncogenic drivers in prostate cancer. *Nat. Genet.* **50**, 645–651 (2018). 29610475 10.1038/s41588-018-0078-z PMC6107367

[52] Sun, X. J. et al. Identification and characterization of a novel human histone H3 lysine 36-specific methyltransferase. *J. Biol. Chem.* **280**, 35261–35271 (2005). 16118227 10.1074/jbc.M504012200

[53] Zeng, X., Zhong, M., Yang, Y., Wang, Z. & Zhu, Y. Down-regulation of RCC1 sensitizes immunotherapy by up-regulating PD-L1 via p27(kip1) /CDK4 axis in non-small cell lung cancer. *J. Cell Mol. Med.* **25**, 4136–4147 (2021). 33630417 10.1111/jcmm.16383 PMC8051708

[54] Garcia-Diaz, A. et al. Interferon Receptor Signaling Pathways Regulating PD-L1 and PD-L2 Expression. *Cell Rep.* **19**, 1189–1201 (2017). 10.1016/j.celrep.2017.04.031 PMC6420824 28494868

[55] Burks, J., Reed, R. E. & Desai, S. D. Free ISG15 triggers an antitumor immune response against breast cancer: a new perspective. *Oncotarget* **6**, 7221–7231 (2015). 25749047 10.18632/oncotarget.3372 PMC4466680

[56] Gide, T. N. et al. Distinct immune cell populations define response to anti-PD-1 monotherapy and anti-PD-1/anti-CTLA-4 combined therapy. *Cancer Cell* **35**, 238–255.e236 (2019). 30753825 10.1016/j.ccell.2019.01.003

[57] Lu, M. et al. Pan-cancer analysis of SETD2 mutation and its association with the efficacy of immunotherapy. *NPJ Precis. Oncol.* **5**, 51 (2021). 34127768 10.1038/s41698-021-00193-0 PMC8203790

[58] He, J., Xu, T., Zhao, F., Guo, J. & Hu, Q. SETD2-H3K36ME3: an important bridge between the environment and tumors. *Front. Genet.* **14**, 1204463 (2023). 37359376 10.3389/fgene.2023.1204463 PMC10288198

[59] Liu, H. et al. ADORA1 inhibition promotes tumor immune evasion by regulating the ATF3-PD-L1 axis. *Cancer Cell* **37**, 324–339.e328 (2020). 32183950 10.1016/j.ccell.2020.02.006

[60] Xiong, W. et al. USP8 inhibition reshapes an inflamed tumor microenvironment that potentiates the immunotherapy. *Nat. Commun.* **13**, 1700 (2022). 35361799 10.1038/s41467-022-29401-6 PMC8971425

[61] Burr, M. L. et al. CMTM6 maintains the expression of PD-L1 and regulates anti-tumour immunity. *Nature* **549**, 101–105 (2017). 28813417 10.1038/nature23643 PMC5706633

[62] Tu, X. et al. PD-L1 (B7-H1) Competes with the RNA Exosome to Regulate the DNA Damage Response and Can Be Targeted to Sensitize to Radiation or Chemotherapy. *Mol. Cell* **74**, 1215–1226.e1214 (2019). 31053471 10.1016/j.molcel.2019.04.005 PMC6737939

[63] Anzalone, A. V. et al. Search-and-replace genome editing without double-strand breaks or donor DNA. *Nature* **576**, 149–157 (2019). 31634902 10.1038/s41586-019-1711-4 PMC6907074

[64] Chen, P. J. et al. Enhanced prime editing systems by manipulating cellular determinants of editing outcomes. *Cell* **184**, 5635–5652.e5629 (2021). 34653350 10.1016/j.cell.2021.09.018 PMC8584034

[65] Nelson, J. W. et al. Engineered pegRNAs improve prime editing efficiency. *Nat. Biotechnol.* **40**, 402–410 (2022). 34608327 10.1038/s41587-021-01039-7 PMC8930418

[66] Walton, R. T., Christie, K. A., Whittaker, M. N. & Kleinstiver, B. P. Unconstrained genome targeting with near-PAMless engineered CRISPR-Cas9 variants. *Science* **368**, 290–296 (2020). 32217751 10.1126/science.aba8853 PMC7297043

[67] Patel, S. J. et al. Identification of essential genes for cancer immunotherapy. *Nature* **548**, 537–542 (2017). 28783722 10.1038/nature23477 PMC5870757

[68] Joncker, N. T., Shifrin, N., Delebecque, F. & Raulet, D. H. Mature natural killer cells reset their responsiveness when exposed to an altered MHC environment. *J. Exp. Med.* **207**, 2065–2072 (2010). 20819928 10.1084/jem.20100570 PMC2947079

[69] Ding, B. et al. Noncoding loci without epigenomic signals can be essential for maintaining global chromatin organization and cell viability. *Sci. Adv.* **7**, eabi6020 (2021). 34731001 10.1126/sciadv.abi6020 PMC8565911

[70] Chen, T. et al. The Genome Sequence Archive Family: toward explosive data growth and diverse data types. *Genom. Proteom. Bioinform.* **19**, 578–583 (2021). 10.1016/j.gpb.2021.08.001 PMC9039563 34400360

[71] Members, C.-N. et al. Database resources of the National Genomics Data Center, China National Center for Bioinformation in 2022. *Nucleic Acids. Res.* **50**, D27–D38 (2022). 34718731 10.1093/nar/gkab951 PMC8728233

