# Supplementary material for: Massively parallel interrogation of human functional variants modulating cancer immunosurveillance
Source: Signal Transduct Target Ther. 2025 Mar 19;10:88. doi: 10.1038/s41392-025-02171-5 (PMC11920242; doi:10.1038/s41392-025-02171-5)
Supplement: Supplementary file 1 — Supplementary Material [file 41392_2025_2171_MOESM1_ESM.docx]

Supplementary Materials for

**Massively parallel interrogation of human functional variants**

**modulating cancer immunosurveillance**

Ying Liu, Yongshuo Liu, Xuran Niu, Ang Chen, Yizhou Li, Ying Yu, Binrui Mo, Zhiheng Liu, Tao Xu, Jie Cheng, Zeguang Wu, and Wensheng Wei

Correspondence to: wswei@pku.edu.cn (W.W.)

**This PDF file includes:**

Figures. S1 to S10

NGS analysis of editing outcomes of sgRNAs

**Other Supplementary Materials for this manuscript include the following:**

Uncropped blots [separate file]

Tables S1 to S8 [separate file]


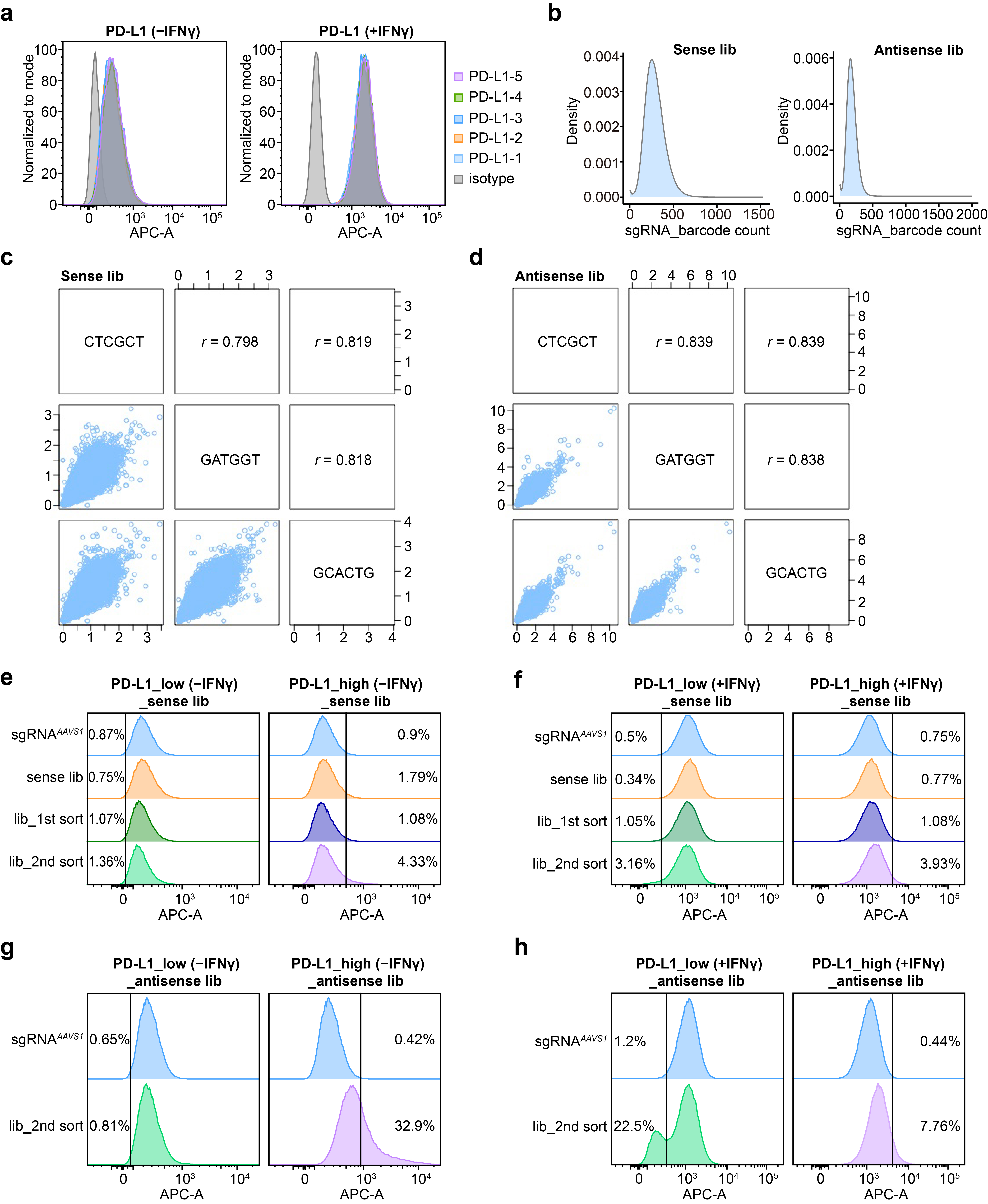


**Figure. S1. Identification of regulatory S/T/Y residues of PD-L1 in the menlonoma cell line A375 using FACS-based screens.**

**a**, Flow cytometry histograms of surface PD-L1 level using varied concentrantion of antibody for staining in the absence of IFNγ (left) and upon IFNγ treatment (right). Cell surface PD-L1 was analysed following incubation without or with 100 ng/mL IFNγ for 48 h. PD-L1-5, PD-L1-4, PD-L1-3, PD-L1-2 and PD-L1-1 respectively indicates 5 μL, 4 μL, 3 μL, 2 μL and 1 μL PD-L1 antibody per million A375-ABEmax cells used for each staining reaction. Isotype indicates 5 μL isotype antibody per million A375-ABEmax cells used for staining. **b**, sgRNA distribution of Day 24 library cells from sense S/T/Y library (sense lib) and antisense S/T/Y library (antisense lib). **c–d**, Correlation analysis between three iBARs from sense lib (c) and antisense lib (d). *r*: Pearson correlation coefficient. **e–f**, Flow cytometry histograms depicting sorted and unsorted mutagenized populations stained by PD-L1 antibody in the absence of IFNγ (e) and upon IFNγ treatment (f) for sense sense lib. **g–h**, Flow cytometry histograms depicting sorted and unsorted mutagenized populations stained by PD-L1 antibody in the absence of IFNγ (g) and upon IFNγ treatment (h) for antisense lib.


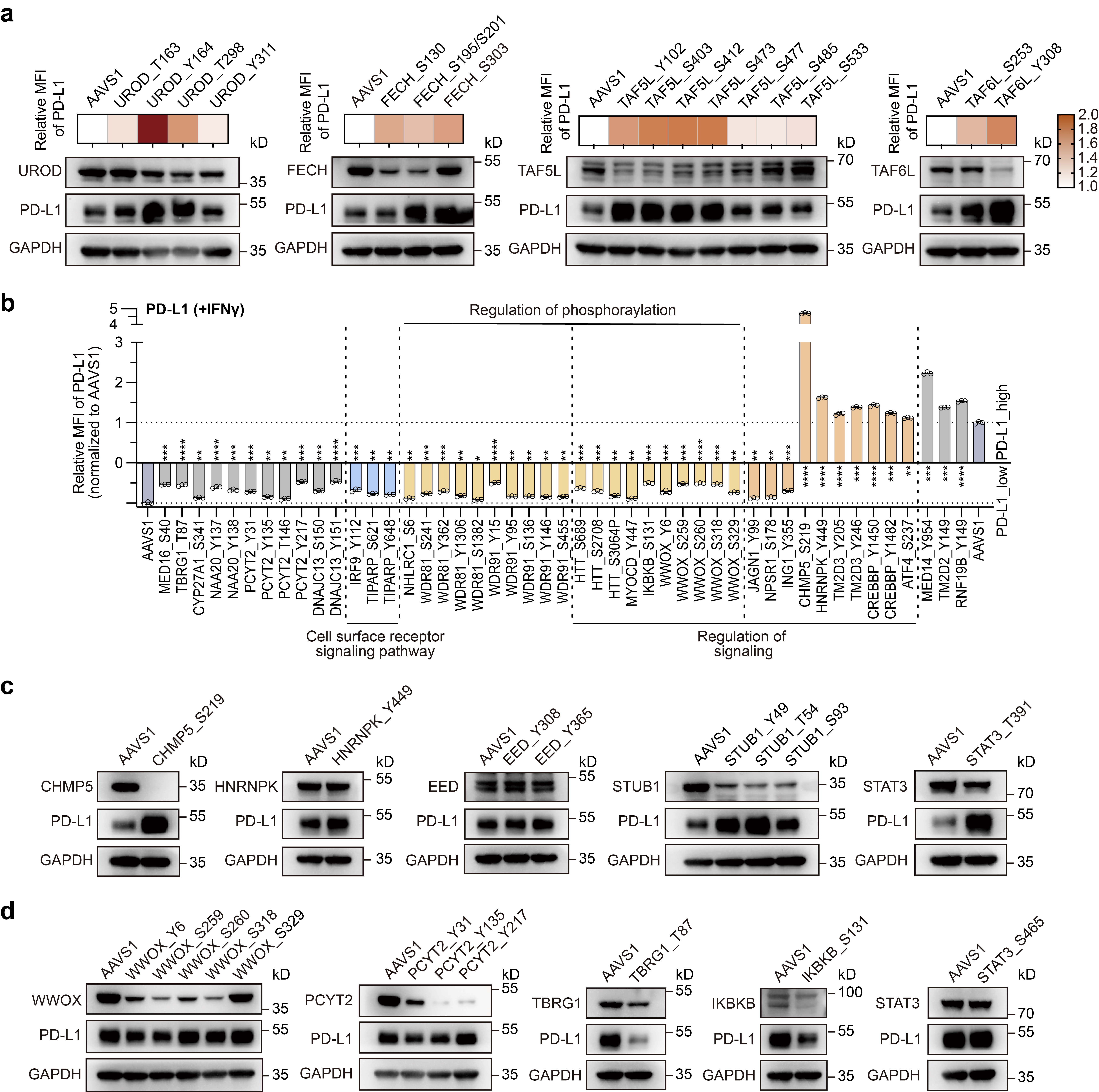


**Figure. S2. Validation of negative and positive regulatory S/T/Y residues of PD-L1 in A375 cells.** **a**, Protein expression levels of UROD, FECH, TAF5L or TAF6L, and PD-L1 in the indicated A375 mutant cells without IFNγ stimulation. The upper heatmap shows the relative surface PD-L1 level of A375 cells with each mutation according to the results of flow cytometric analysis from Fig. 2c. The lower IB analysis shows the overall expression level of each indicated protein and PD-L1 for the corresponding mutant. **b**, Individual validations of negative and postive regulators of cell surface PD-L1 in A375 cells upon IFNγ treatment by flow cytometry analysis. Cell surface PD-L1 was analysed following incubation with 100 ng/mL IFNγ for 48 h. The method to generate relative MFI of PD-L1 and the statistics are the same as those shown in Fig. 2d. **c–d**, IB analysis of the indicated proteins and PD-L1 in A375 cells infected with sgRNAs targeting identified negative regulatory S/T/Y residues (c) and positive regulatory residues (d) treated with IFNγ.


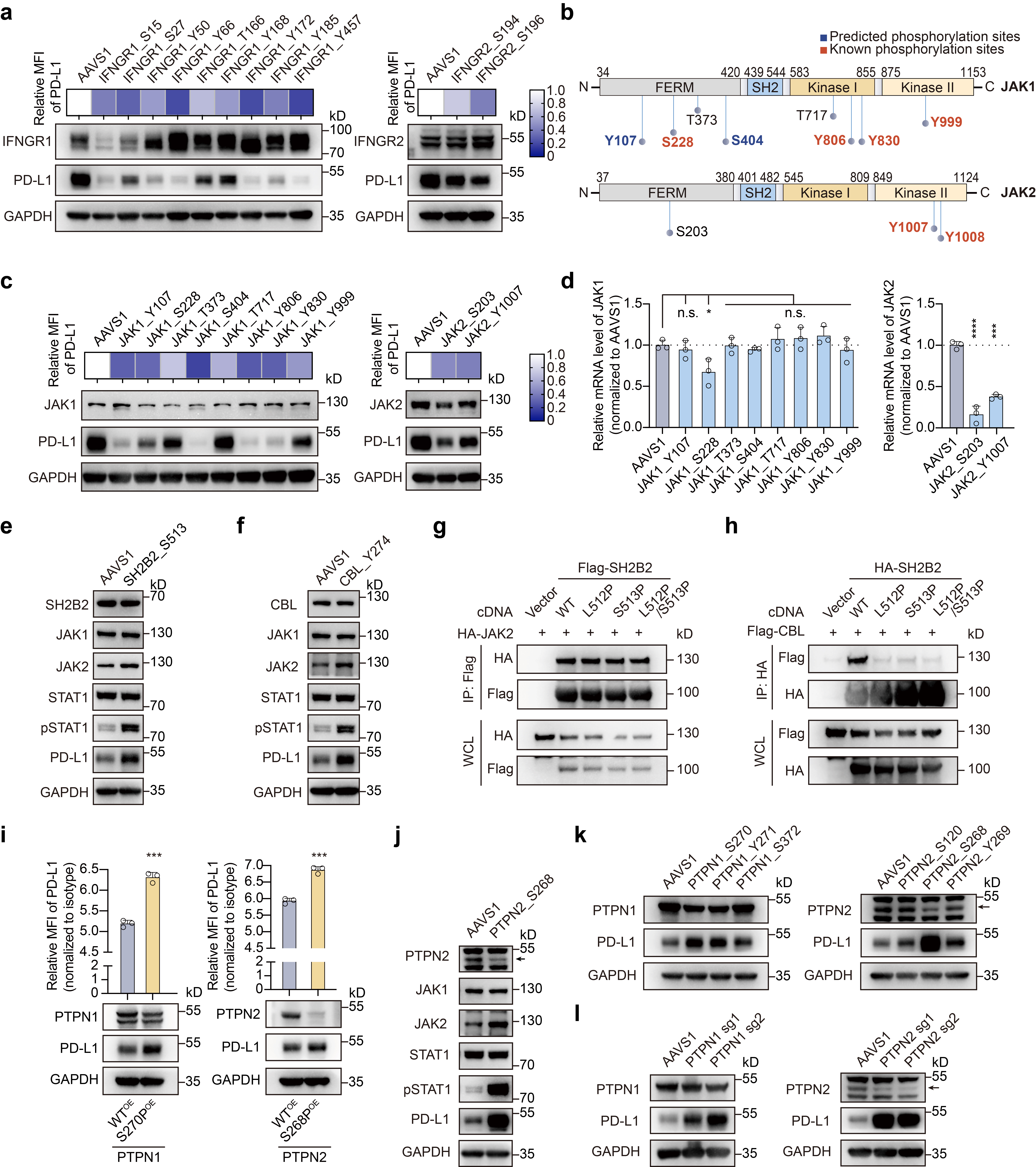


**Figure. S3. Effects of candidate S/T/Y residues involved in IFNγ signal transduction.**

**a**, Protein expression levels of IFNGR1 or IFNGR2 and PD-L1 in the indicated A375 mutant cells treated with IFNγ. The upper heatmap shows the relative surface PD-L1 level of each mutant according to the results of flow cytometric analysis from Fig. 2d. The lower IB analysis shows the total protein level of IFNGR1 or IFNGR2 and PD-L1 for each corresponding mutant. **b**, Distribution of identified S/T/Y residues on JAK1 and JAK2 protein. The regulatory residues are marked below each protein structure, which indicate positive regulators. The relative length of each vertical line reflects the regulaory effect of the indicated residue according to the results of flow cytometric analysis from Fig. 2d. **c**, Protein expression levels of JAK1 or JAK2 and PD-L1 in the indicated A375 mutant cells treated with IFNγ. The upper heatmap shows the relative surface PD-L1 level of each mutant according to the results of flow cytometric analysis from Fig. 2d. The IB analysis shows the total protein level of JAK1 or JAK2 and PD-L1 for each corresponding mutant. **d**, Relative mRNA expression levels of *JAK1* or *JAK2* in the indicated A375 mutant cells treated with IFNγ. The mRNA level of each sample was quantified by real-time qPCR and normalized by *GAPDH*. For each mutant cells, the indicated relative mRNA level was normalized to that of *AAVS1*-targeting control cells. Data are presented as the mean ± SD (n = 3). *P* values were calculated using Student’s *t* test, **P* < 0.05; ****P* < 0.001; *****P* < 0.0001; n.s., not significant. **e**, IB analysis of typical JAK/STAT signaling components, SH2B2, and PD-L1 in A375 cells infected with respective sgRNA targeting *AAVS1* and SH2B2_S513. **f**, IB analysis of typical JAK/STAT signaling components, CBL, and PD-L1 in A375 cells infected with respective sgRNA targeting *AAVS1* and CBL_Y274. **g**, IB analysis of anti-Flag IPs and WCLs of 293T cells co-transfected with the indicated plasmids expressing HA-tagged JAK2 and Flag-tagged SH2B2 WT or variants. **h**, IB analysis of anti-HA IPs and WCLs of 293T cells co-transfected with the indicated plasmids expressing Flag-tagged CBL and HA-tagged SH2B2 WT or variants. **i**, Protein expression levels of PTPN1/PTPN2 and PD-L1 in A375 cells overexpressing PTPN1/PTPN2 WT and respective mutant cDNA. The upper histogram shows the surface PD-L1 level of each indicated sample by flow cytometric analysis. The lower IB analysis shows the total protein level of PTPN1/PTPN2 and PD-L1 for each indicated sample. *P* values were calculate by Student’s *t* test, ****P* < 0.001. **j**, IB analysis of typical JAK/STAT signaling components, PTPN2, and PD-L1 in A375 cells infected with respective gRNA targeting *AAVS1* and PTPN2_S268. **k**, IB analysis of PTPN1 or PTPN2 and PD-L1 in A375-ABEmax cells infected with respective sgRNA targeting *AAVS1* and candidate residues of PTPN1 (left) or PTPN2 (right). **l**, IB analysis of PTPN1 or PTPN2 and PD-L1 in A375-Cas9 cells infected with respective sgRNA targeting *AAVS1* and *PTPN1* (left) or *PTPN2* (right) gene.


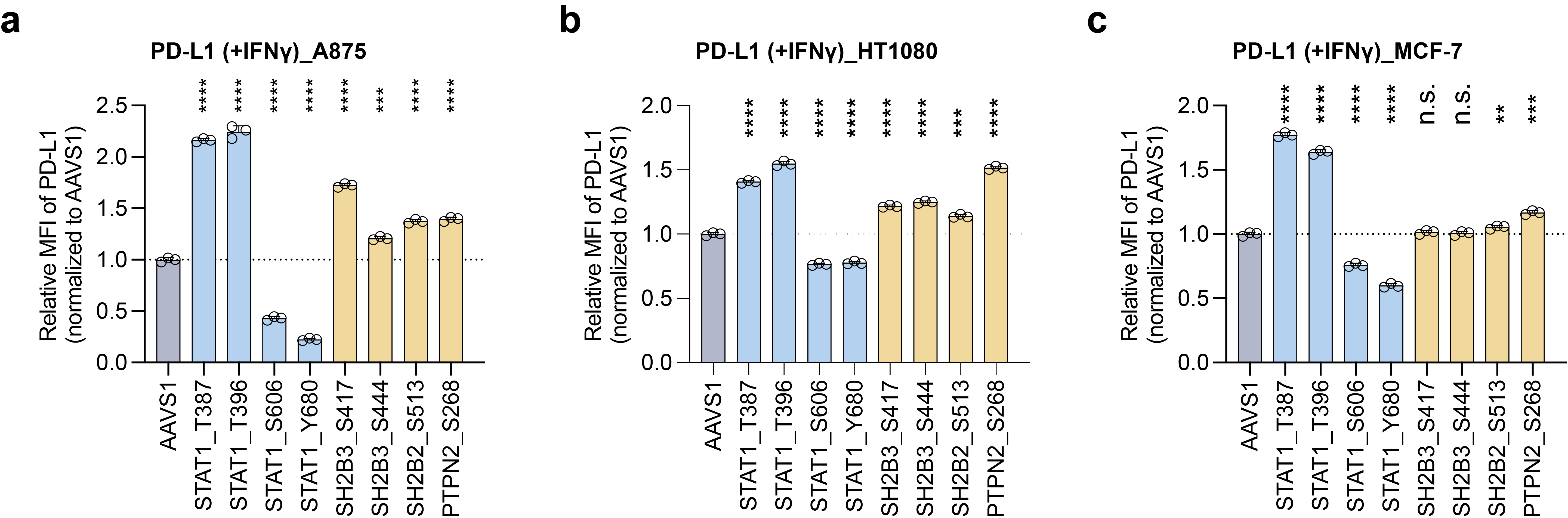


**Figure. S4. Assessment of cell surface PD-L1 expression of representative regulators related to JAK-STAT signaling pathway in A875 (a), HT1080 (b) and MCF-7 (c) cells by flow cytometry analysis.** Cell surface PD-L1 was analyzed following incubation without or with 100 ng/mL IFNγ for 48 h. The method to generate relative MFI of HLA-I and the statistics are the same as those shown in Fig. 2c–d.


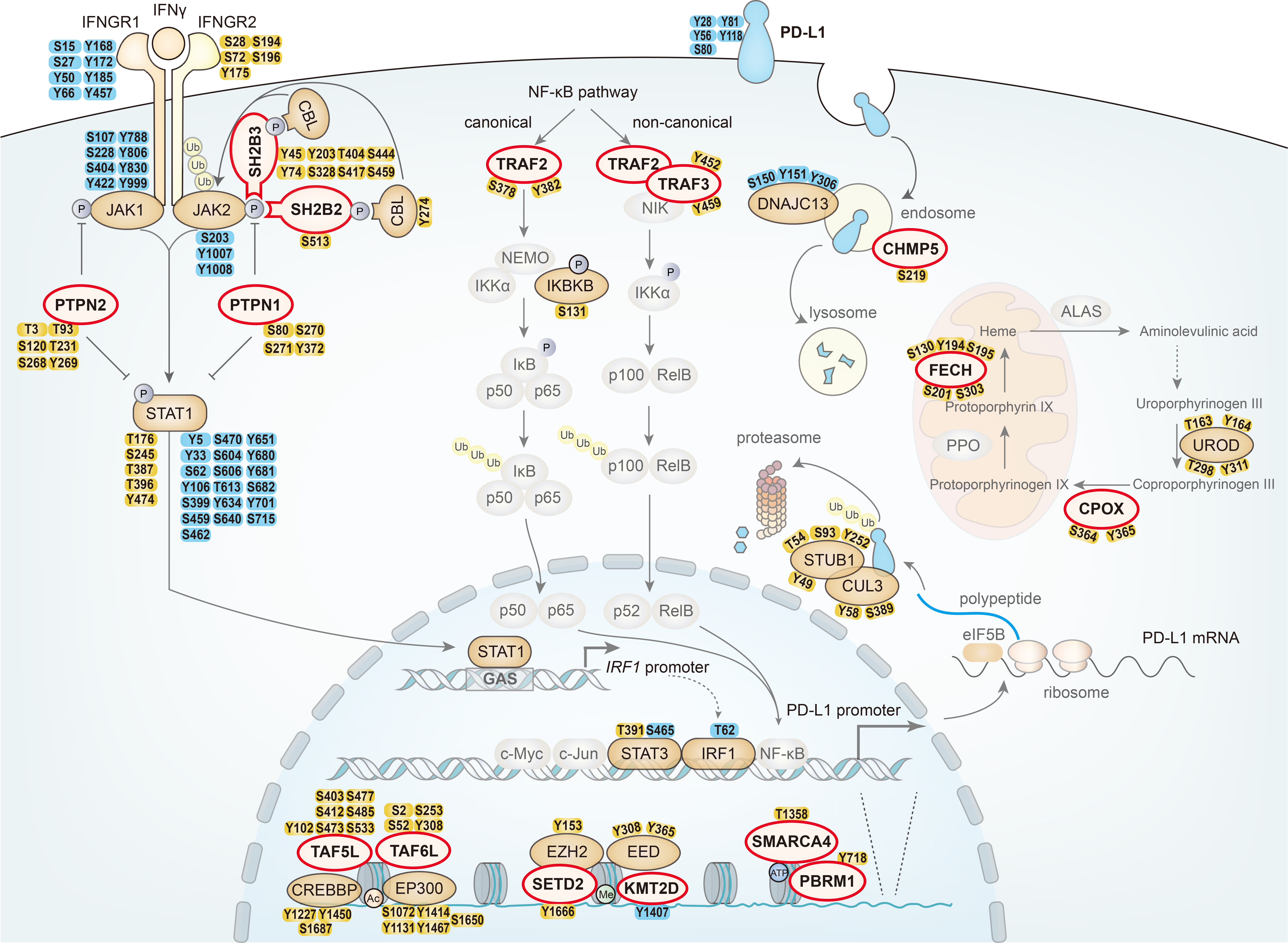


**Figure. S5. Schematic diagram of the PD-L1 regulatory network.** Based on the screening results, functional validation and reported studies of related mechanisms, we summarized typical regulatory genes of PD-L1 and labeled representative S/T/Y residues on related proteins.


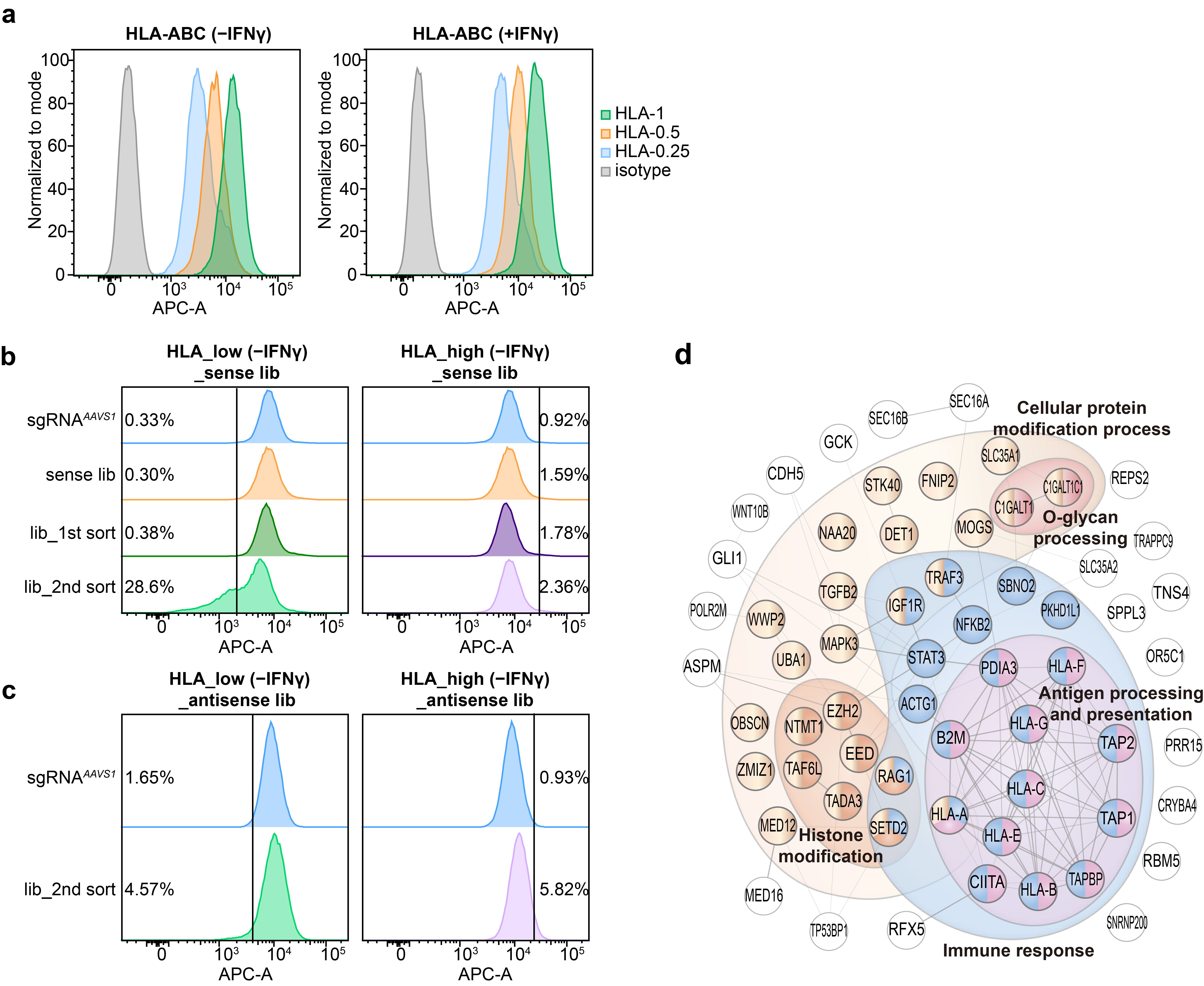


**Figure. S6. Identification of regulatory S/T/Y residues of HLA-I in A375 cells using FACS-based screens.**

**a**, Flow cytometry histograms of surface HLA-I level using varied concentrantion of antibody for staining in the absence of IFNγ (left) and upon IFNγ treatment (right). HLA-1, HLA-0.5, and HLA-0.25 respectively indicates 1 μL, 0.5 μL, and 0.25 μL PD-L1 antibody per million A375-ABEmax cells is used for each staining reaction. Isotype indicates 1 μL isotype antibody per million A375-ABEmax cells is used for staining. **b–c**, Flow cytometry histograms depicting sorted and unsorted mutagenized populations stained by HLA-ABC antibody in the absence of IFNγ for sense (b) and antisense (c) S/T/Y library. **d**, STRING analysis of related genes haboring top-ranked mutations from the HLA-I screens.


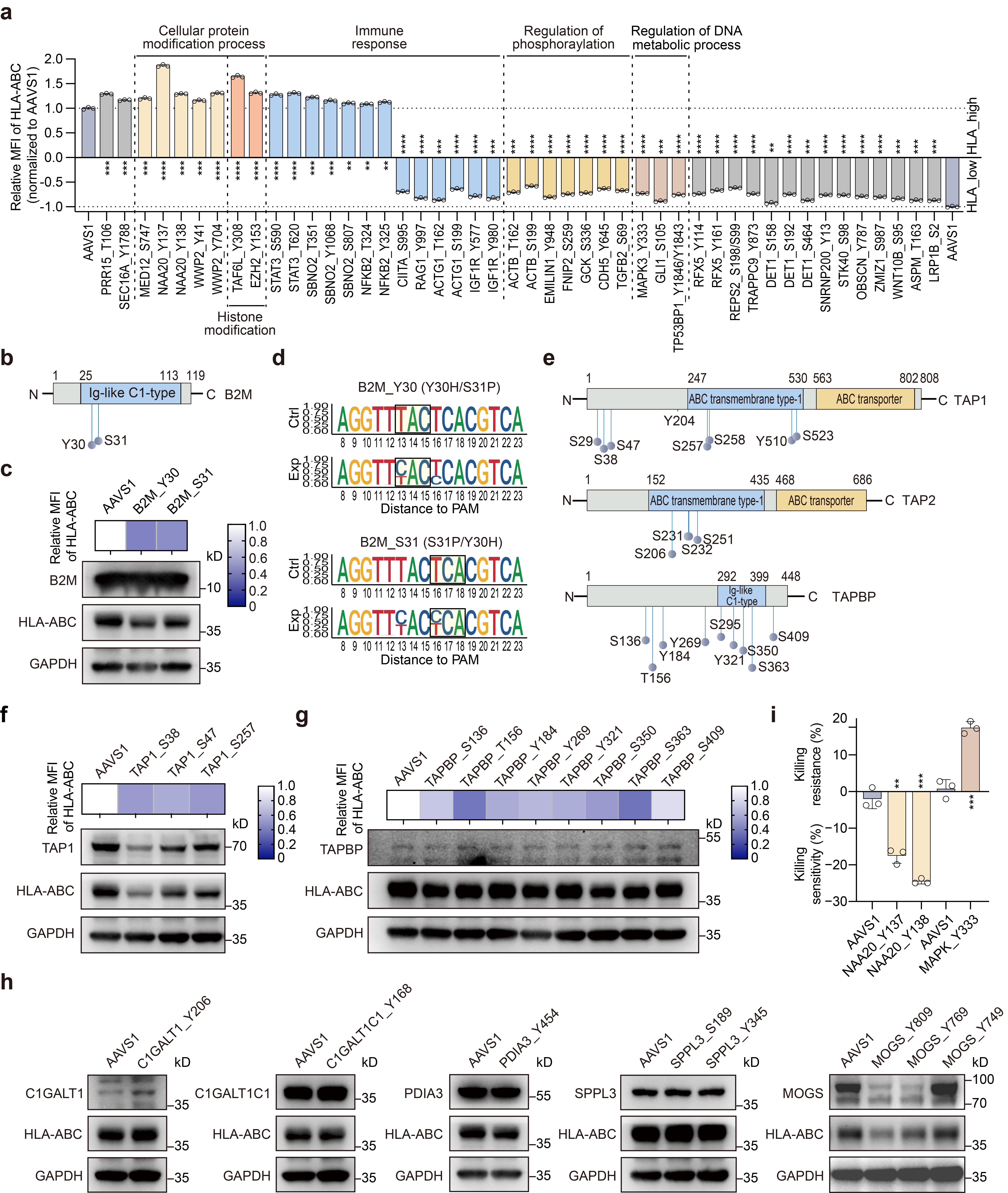


**Figure. S7. Validation of candidate regulatory S/T/Y residues of HLA-I in A375 cells.**

**a**, Assessment of cell surface HLA-I expression of negative and postive regulators in A375 cells without IFNγ treatment by flow cytometry analysis. The method used to generate the relative MFI of HLA-I and the statistical analysis are the same as those shown in Fig. 2c–d. **b**, Distribution of identified S/T/Y residues on B2M protein. The way of labeling each residue is the same as Fig. 3a. **c**, Protein expression levels of B2M and HLA-ABC in A375 cells infected with respective sgRNA targeting *AAVS1* and candidate B2M residues. The upper histogram shows the surface HLA-I level of each indicated sample by flow cytometric analysis. The lower IB analysis shows the total protein level of B2M and HLA-ABC for each indicated sample. **d**, Editing outcomes of sgRNA targeting B2M_Y30 and B2M_S31 by NGS analysis. Ctrl and Exp respectively indicates the WT and mutated sequence in A375 cells. **e**, Distribution of identified S/T/Y residues on TAP1, TAP2 and TAPBP protein. The way of labeling each residue is the same as Fig. 3a. **f**, Protein expression levels of TAP1 and HLA-ABC in A375 cells infected with sgRNA targeting *AAVS1* and candidate residues of TAP1. The upper histogram shows the surface HLA-I level of each indicated sample by flow cytometric analysis. The lower IB analysis shows the total protein level of TAP1 and HLA-ABC for each indicated sample. **g**, Protein expression levels of TAPBP and HLA-ABC in A375 cells infected with sgRNA targeting *AAVS1* and candidate residues of TAPBP. The upper histogram shows the surface HLA-I level of each indicated sample by flow cytometric analysis. The lower IB analysis shows the total protein level of TAPBP and HLA-ABC for each indicated sample. **h**, IB analysis of the indicated proteins and HLA-ABC in A375 cells infected with sgRNAs targeting identified S/T/Y residues enriched on glycosylation-related genes. i, Killing resistance and sensitivity of A375 cells infected with sgRNAs targeting candidate residues to expanded anti-NY-ESO-1 CD8+ T cells. Data are presented as the mean ± SD (n = 3). *P* values were calculated using two-tailed Student’s t test, ***P* < 0.01, ****P* < 0.001.


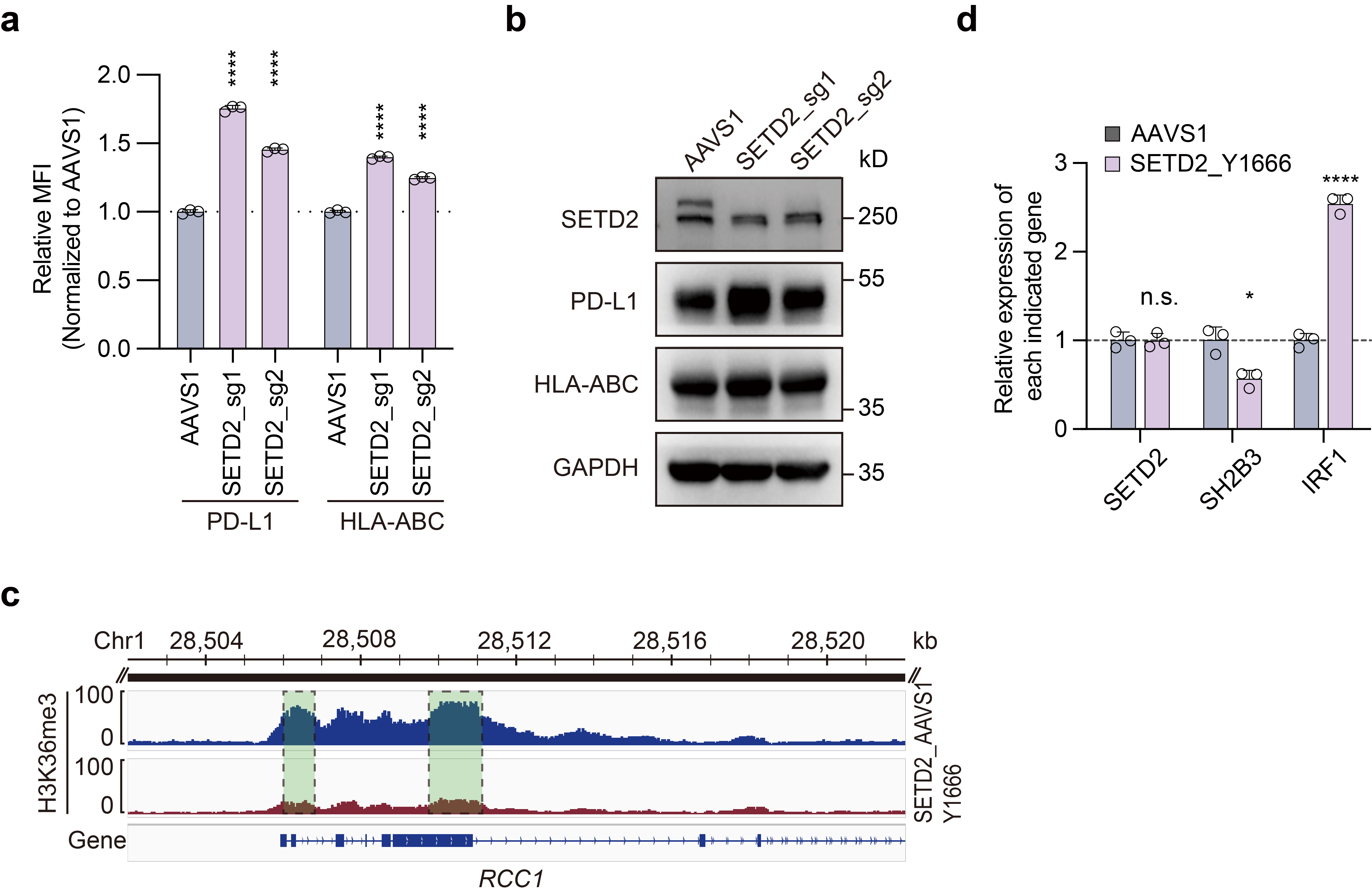


**Figure. S8. Both the SETD2 gene and the SETD2_Y1666 residue act as co-regulators of PD-L1 and HLA-I.**

**a**, Assessment of cell surface PD-L1 and HLA-I expression of SETD2-KO cells in A375 cells by flow cytometry analysis. SETD2_sg1 and SETD2_sg2 indicate different sgRNAs targeting SETD2. The relative MFI of surface PD-L1 or HLA-I for each SETD2-KO sample represents the ratio normalized to the MFI of *AAVS1*-targeting control cells. Data are presented as the mean ± SD (n = 3). *P* values were calculated using two-tailed Student’s *t* test, *****P* < 0.0001. **b**, Immunoblotting analysis of SETD2, PD-L1 and HLA-ABC in A375 cells infected with respective sgRNA targeting *AAVS1* and SETD2. **c**, ChIP-seq tracks for H3K36me3 at *RCC1* gene locus between SETD2_Y1666-targeted A375 mutant cells and *AAVS1*-targeted A375 control cells. **d**, Relative mRNA expression levels of *SETD2*, *SH2B3* and *IRF1* in A375 cells infected with respective sgRNA targeting *AAVS1* and SETD2_Y1666. The data were calculated using the same method as in Fig. 4g and are presented as the mean ± SD (n = 3). *P* values were calculated using Student’s *t* test, **P* < 0.05; *****P* < 0.0001; n.s., not significant. All cell samples were treated with 100 ng/mL IFNγ for 48 h.


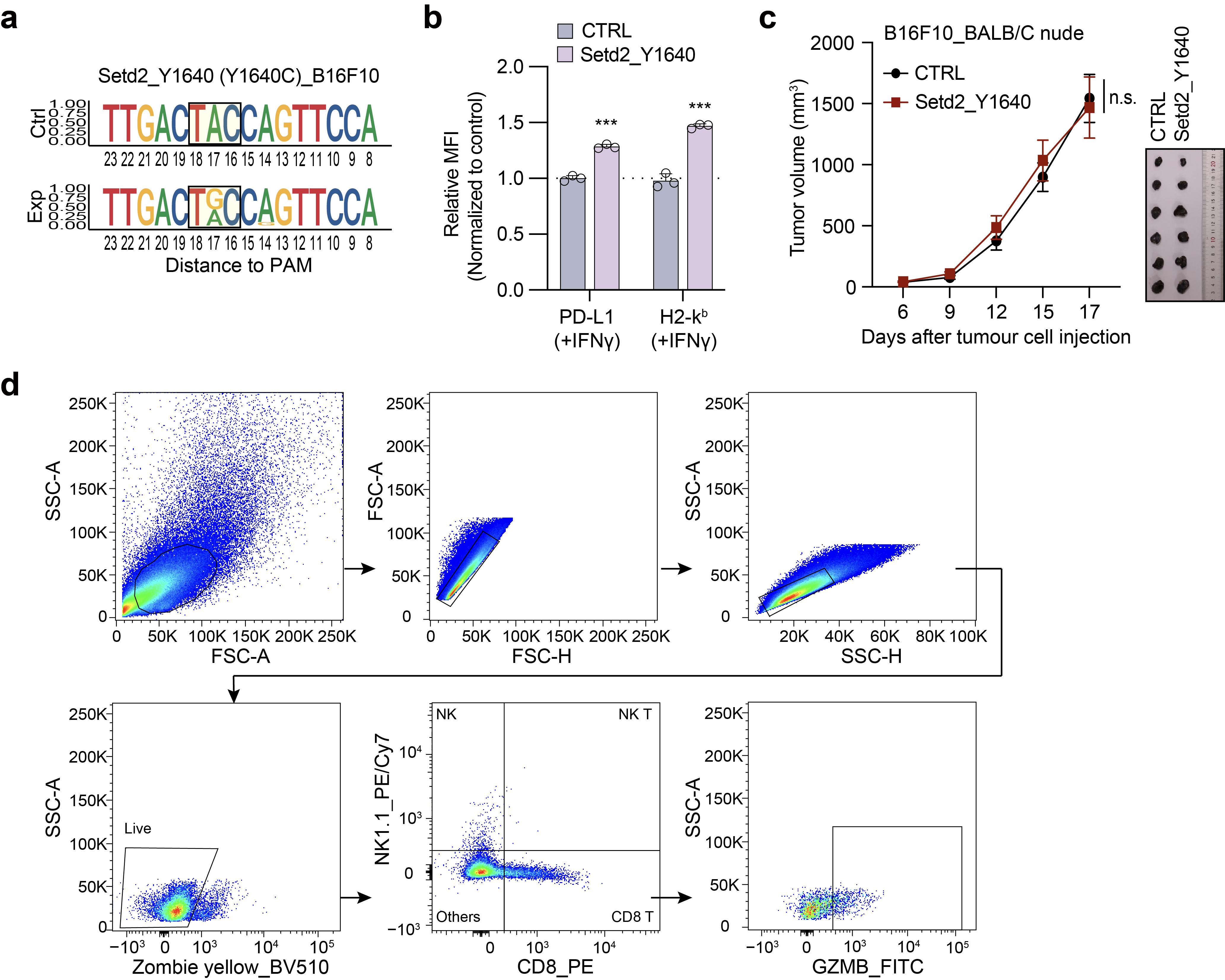


**Figure. S9. Assessment of the tumor progression and ICB response of SETD2_Y1666 (Setd2_Y1640) *in vivo*.**

**a**, Editing outcomes of sgRNA targeting Setd2_Y1640 by NGS analysis. Ctrl and Exp respectively indicates the WT and mutated sequence in B16F10 cells. **b**, Assessment of cell surface PD-L1 and H2-K^b^ expression of Setd2_Y1640 in B16F10 cells with IFNγ treatment by flow cytometry analysis. The method used to generate the relative MFI of PD-L1 or HLA-I and the statistical analysis are the same as those shown in Fig. 2c–d. **c**, Longitudinal tumor size of the control and Setd2_Y1640-targeted B16F10 tumor in BALB/C nude mice. Data are presented as the mean ± S.E.M. (n = 6 mice/group) for each group at each time point. *P* values were calculated using Two-way ANOVA with Benjamini-Hochberg adjustment for multiple testing, n.s., not significant. **d,** Flow cytometry analysis strategy of GzmB/CD8+ cells from the mouse model.


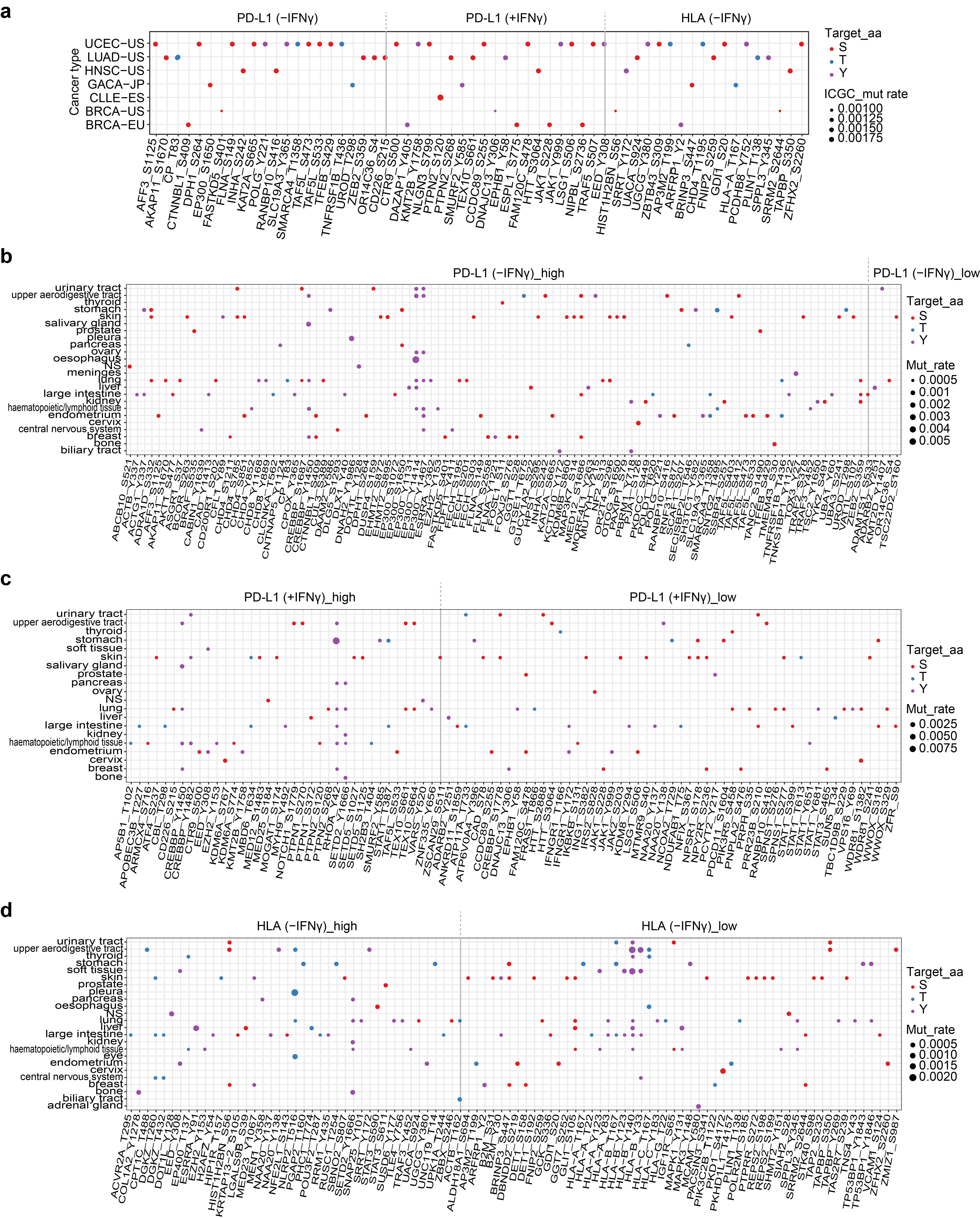


**Figure. S10. Clinical relevance of identified residues according to ICGC and COSMIC database.**

**a**, Schematic of partial residues identified from PD-L1 and HLA-I screens with clinical relevance according to ICGC database. The residues shown in this figure and those in Fig. 6h collectively constitute the overall clinically relevant mutations according to ICGC database. **b–d**, Schematic of all functional residues identified from PD-L1 (without IFNγ treatment) (b), PD-L1 (with IFNγ treatment) (c) and HLA-I (d) screens with clinical relevance according to COSMIC database.

**NGS analysis of editing outcomes of sgRNAs**

**
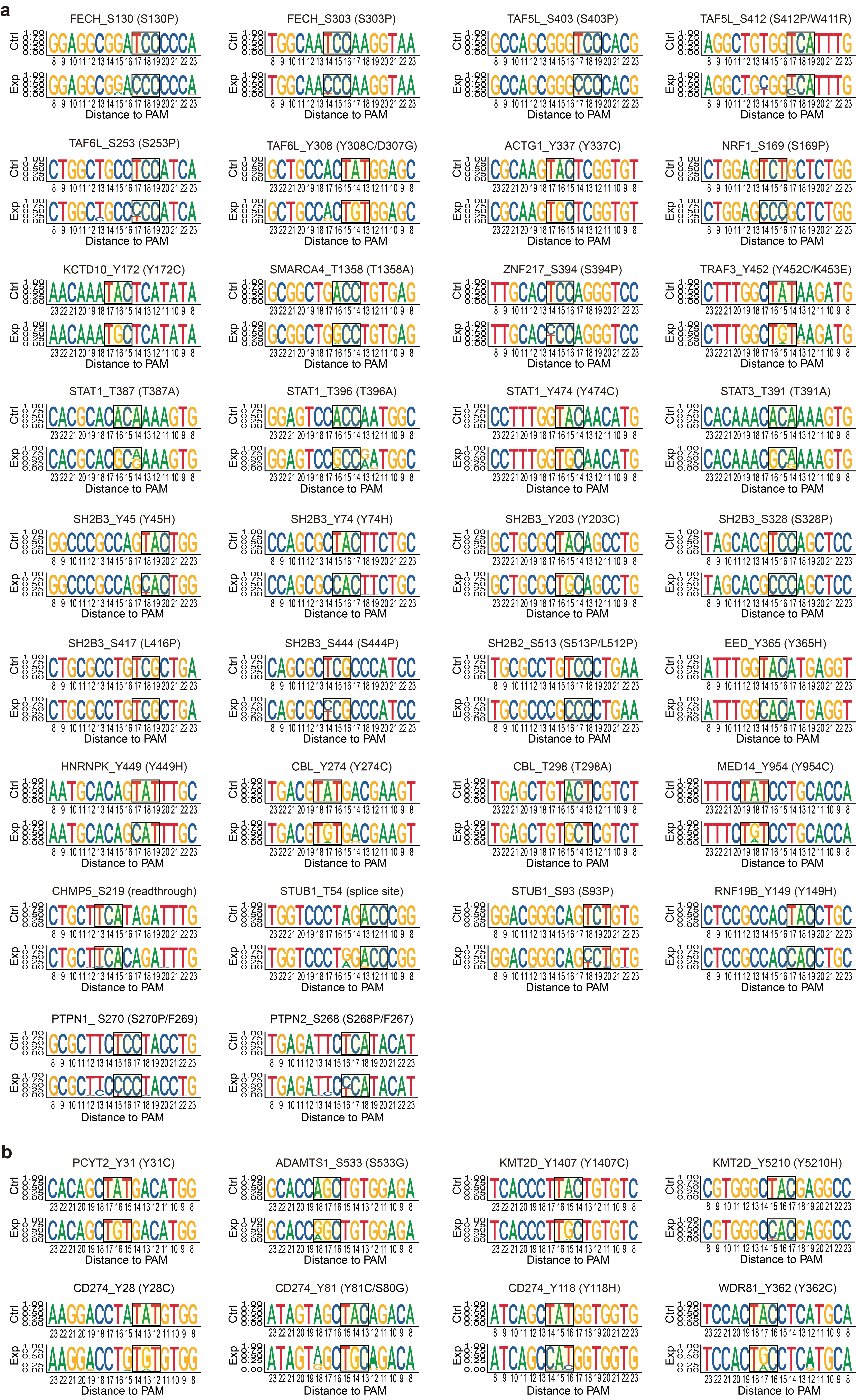
**

**
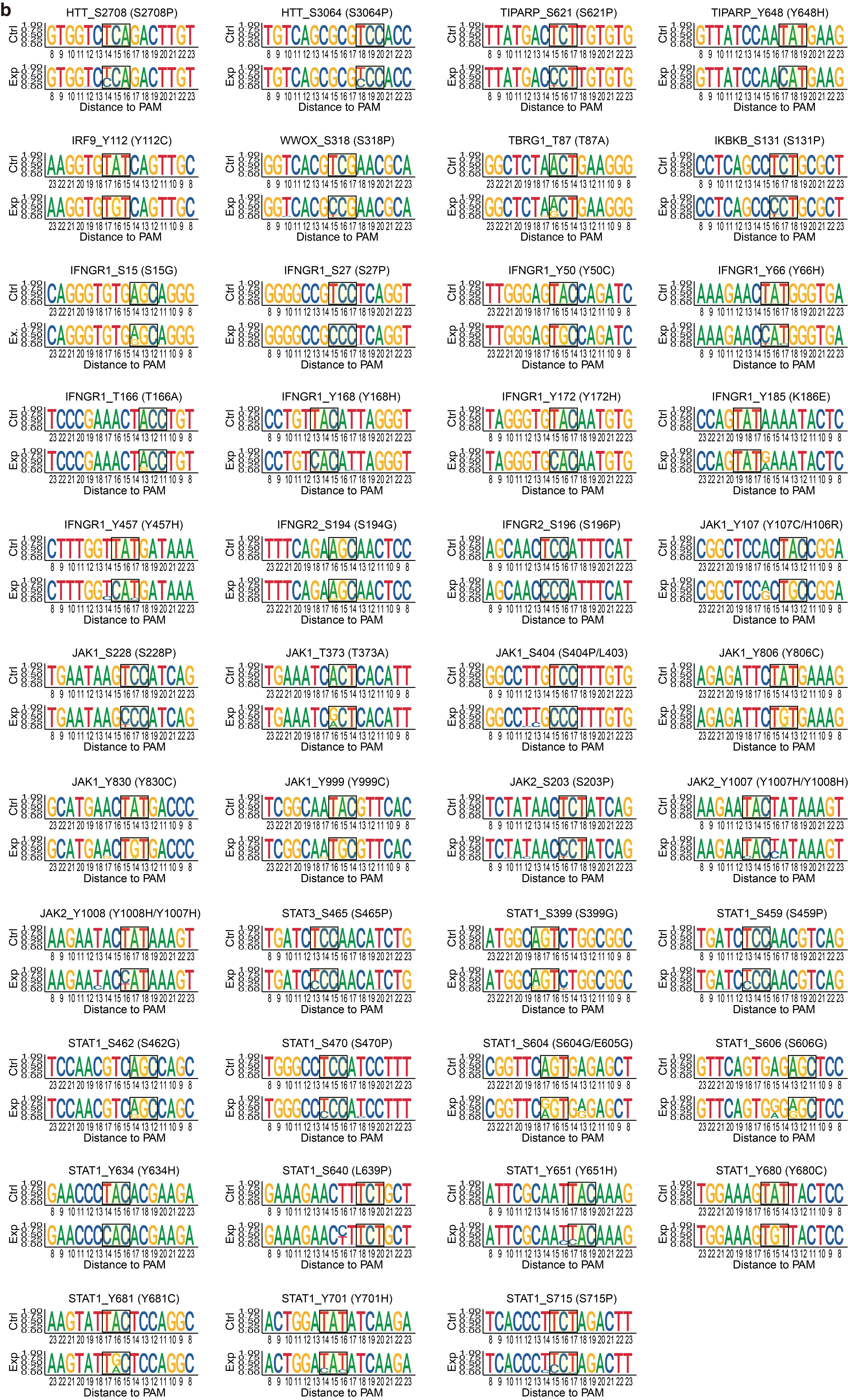
**

**
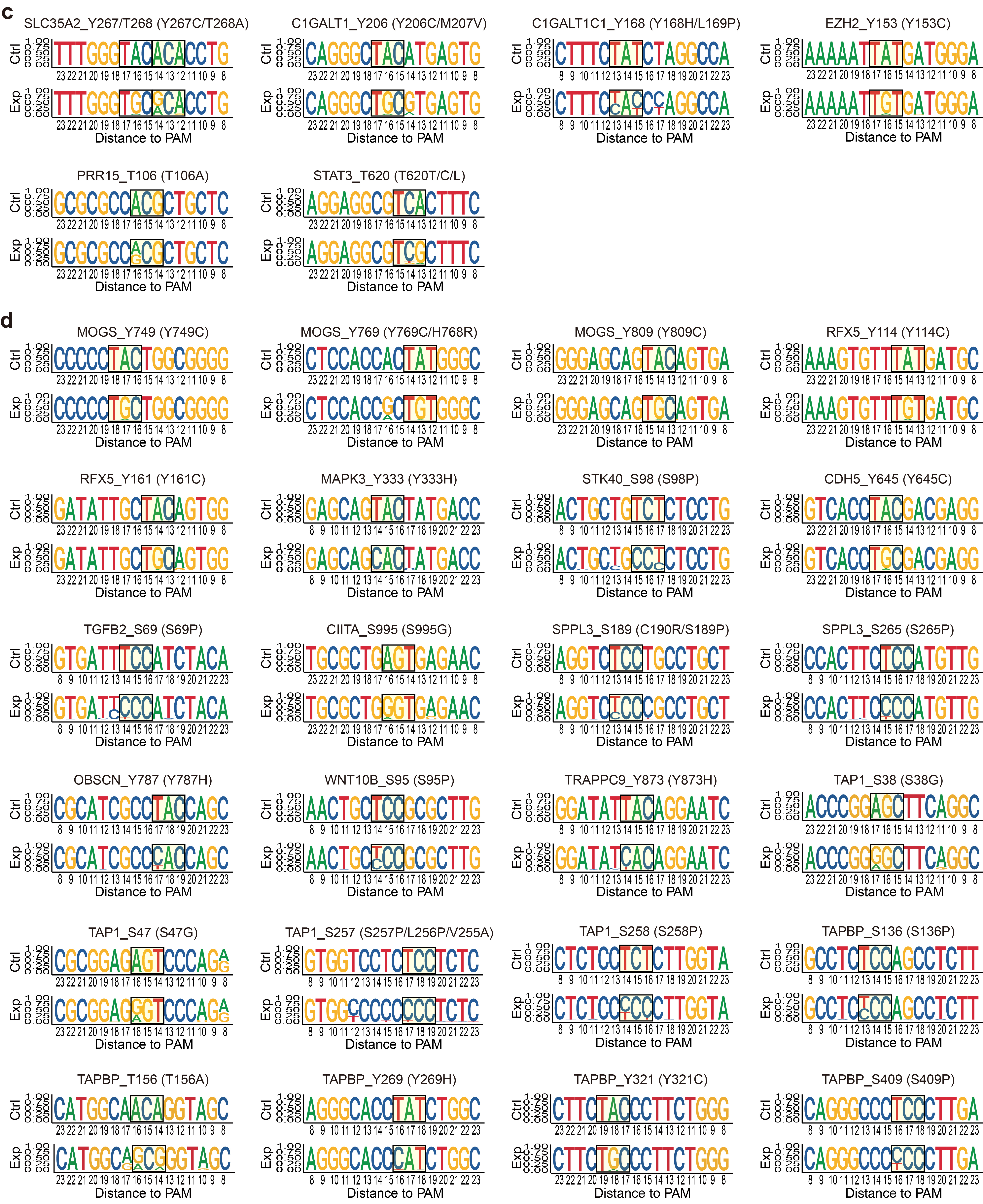
**

**Editing outcomes of sgRNAs targeting different candidate sites enriched from PD-L1^high^ (a), PD-L1^low^ (b), HLA-I^high^ (c) and HLA-I^low^ (d) screens by NGS analysis.**

Ctrl and Exp respectively indicates the WT and mutated sequence in A375 cells.

**Supplementary Tables**

Table S1 Regulatory residues enriched from PD-L1_high screens without IFNγ treatment

Table S2 Regulatory residues enriched from PD-L1_low screens without IFNγ treatment

Table S3 Regulatory residues enriched from PD-L1_high screens with IFNγ treatment

Table S4 Regulatory residues enriched from PD-L1_low screens with IFNγ treatment

Table S5 Regulatory residues enriched from HLA-I_high screens

Table S6 Regulatory residues enriched from HLA-I_low screens

Table S7 sgRNAs for individual validations

Table S8 Primers for genomic PCR and real-time qPCR
