# Supplementary material for: Massively parallel interrogation of human functional variants modulating cancer immunosurveillance
Source: Signal Transduct Target Ther. 2025 Mar 19;10:88. doi: 10.1038/s41392-025-02171-5 (PMC11920242; doi:10.1038/s41392-025-02171-5)
Supplement: Supplementary file 2 — uncropped blots [file 41392_2025_2171_MOESM2_ESM.pdf]

Fig. 3

b

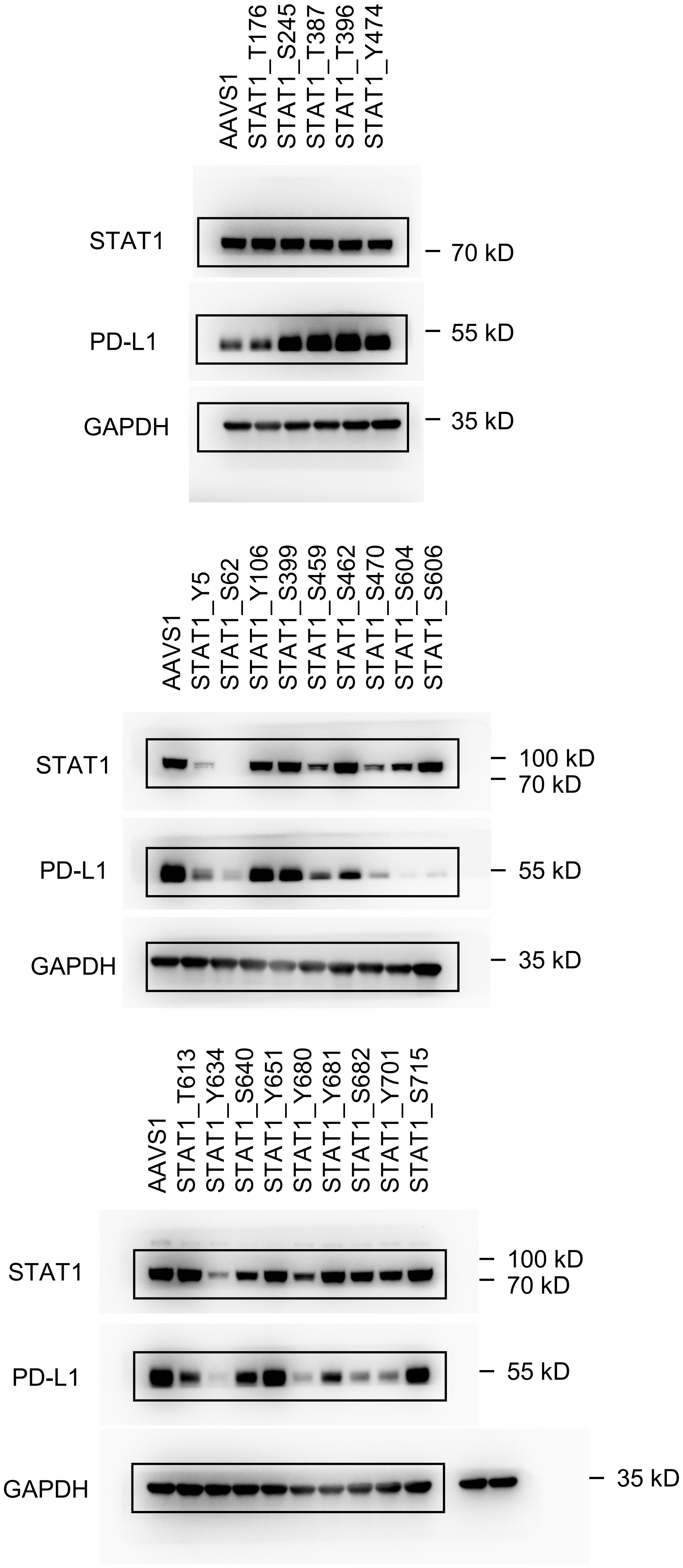
AAVS1
STAT1_T176
STAT1_S245
STAT1_T387
STAT1_T396
STAT1_Y474
STAT1
PD-L1
GAPDH
70 kD
55 kD
35 kD
AAVS1
STAT1_Y5
STAT1_S62
STAT1_Y106
STAT1_S399
STAT1_S459
STAT1_S462
STAT1_S470
STAT1_S604
STAT1_S606
STAT1
PD-L1
GAPDH
100 kD
70 kD
55 kD
35 kD
AAVS1
STAT1_T613
STAT1_Y634
STAT1_S640
STAT1_Y651
STAT1_Y680
STAT1_Y681
STAT1_S682
STAT1_Y701
STAT1_S715
STAT1
PD-L1
GAPDH
100 kD
70 kD
55 kD
35 kD

Fig. 3

f

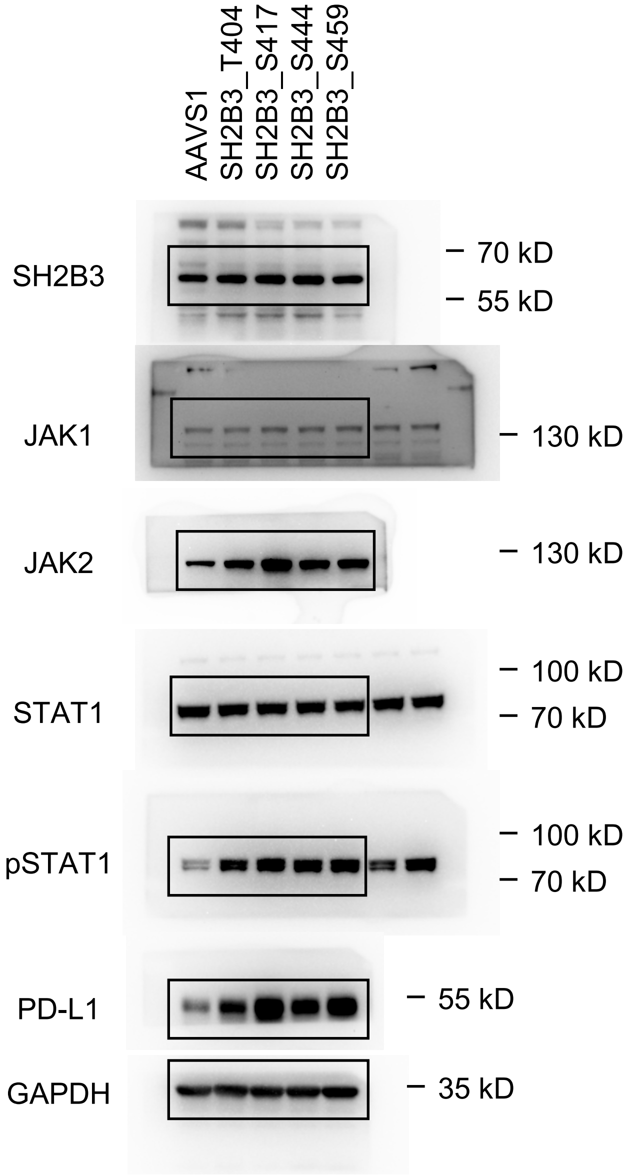
AAVS1
SH2B3_T404
SH2B3_S417
SH2B3_S444
SH2B3_S459
SH2B3
70 kD
55 kD
JAK1
130 kD
JAK2
130 kD
STAT1
100 kD
70 kD
pSTAT1
100 kD
70 kD
PD-L1
55 kD
GAPDH
35 kD

Fig. 3

g

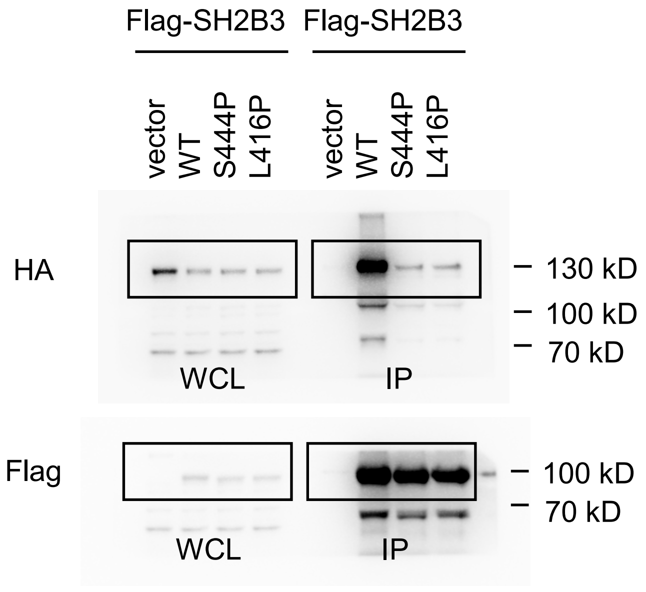
Flag-SH2B3
Flag-SH2B3
vector
WT
S444P
L416P
vector
WT
S444P
L416P
HA
130 kD
100 kD
70 kD
WCL
IP
Flag
100 kD
70 kD
WCL
IP

h

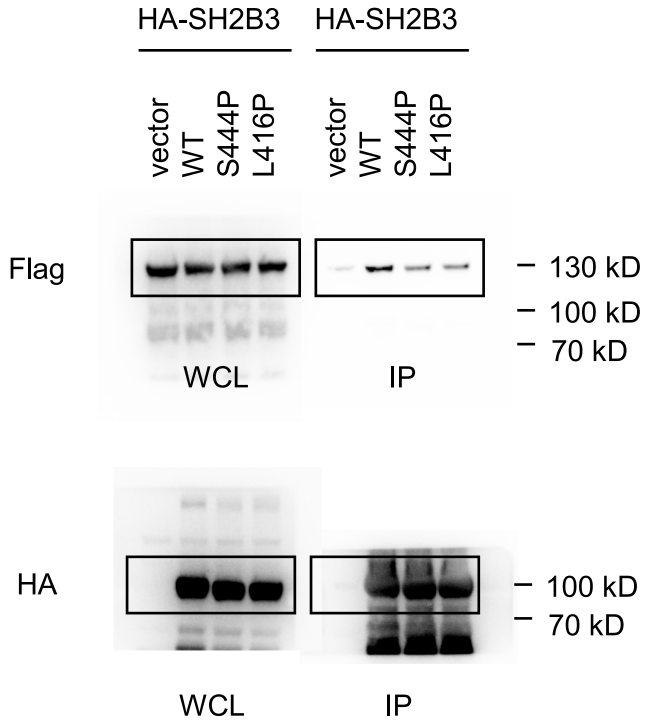
HA-SH2B3
HA-SH2B3
vector
WT
S444P
L416P
vector
WT
S444P
L416P
Flag
130 kD
100 kD
70 kD
WCL
IP
HA
100 kD
70 kD
WCL
IP

Fig. 3

j

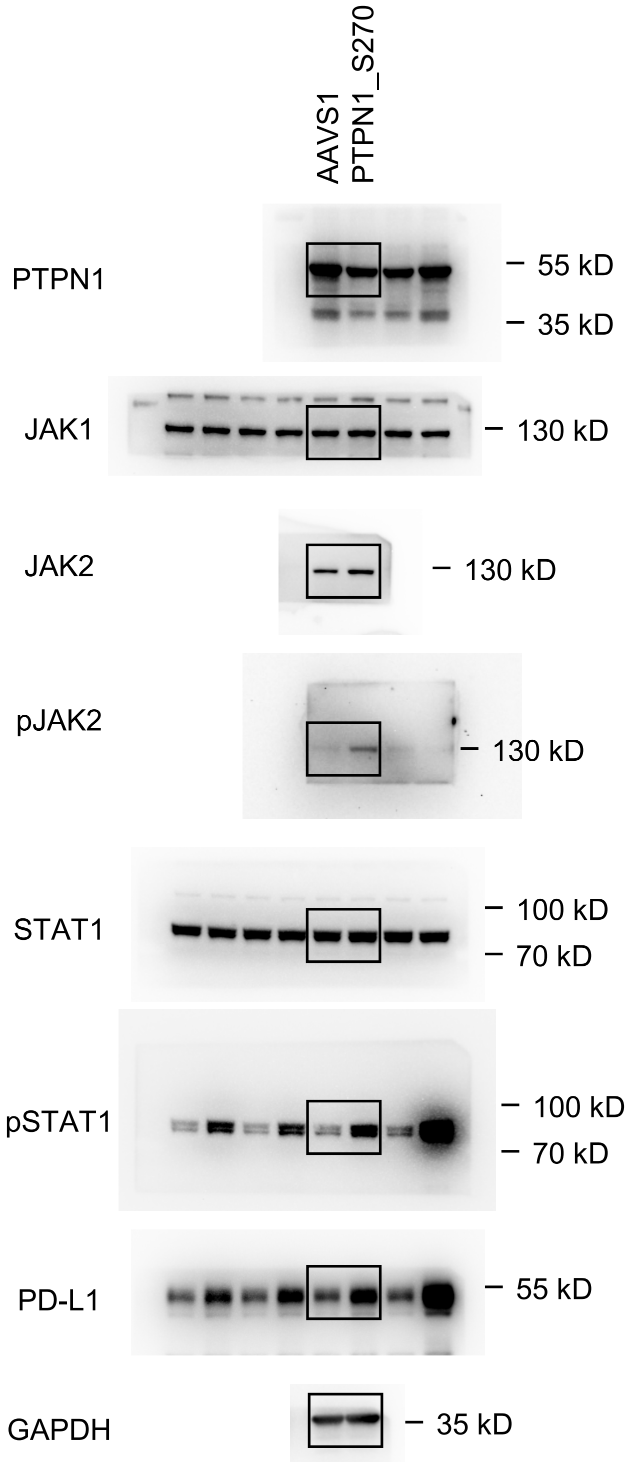

AAVS1
PTPN1_S270
PTPN1
55 kD
35 kD
JAK1
130 kD
JAK2
130 kD
pJAK2
130 kD
STAT1
100 kD
70 kD
pSTAT1
100 kD
70 kD
PD-L1
55 kD
GAPDH
35 kD

Fig. 4

f

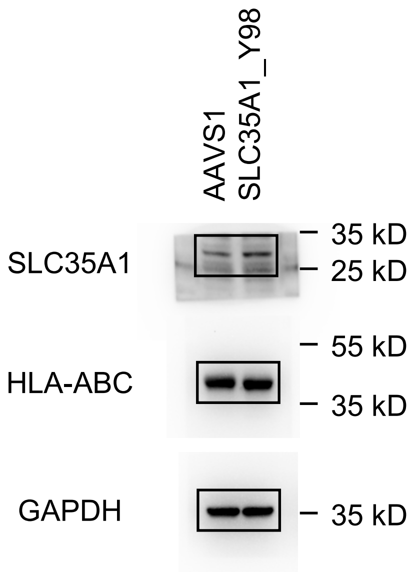
AAVS1
SLC35A1_Y98
SLC35A1
35 kD
25 kD
HLA-ABC
55 kD
35 kD
GAPDH
35 kD

Fig. 5

d

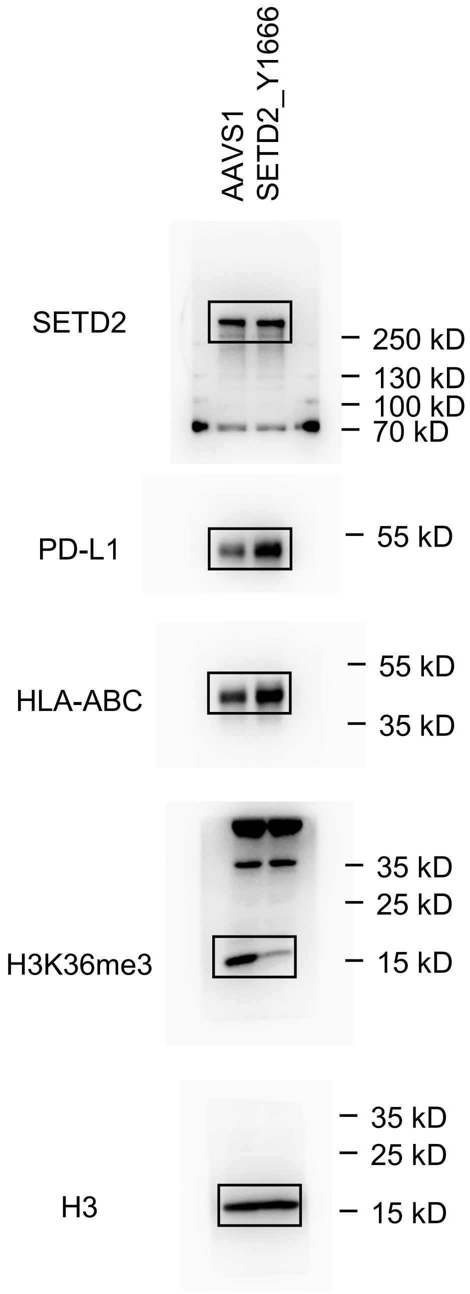
AAVS1
SETD2_Y1666
SETD2
250 kD
130 kD
100 kD
70 kD
PD-L1
55 kD
HLA-ABC
55 kD
35 kD
H3K36me3
35 kD
25 kD
15 kD
H3
35 kD
25 kD
15 kD

Fig. 5

h

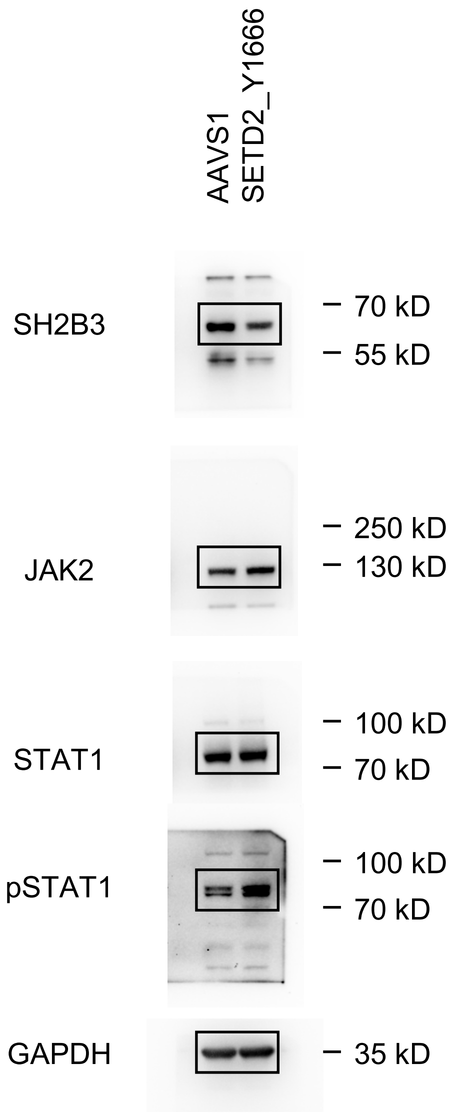

AAVS1
SETD2_Y1666
SH2B3
70 kD
55 kD
JAK2
250 kD
130 kD
STAT1
100 kD
70 kD
pSTAT1
100 kD
70 kD
GAPDH
35 kD

Figure. S2

a

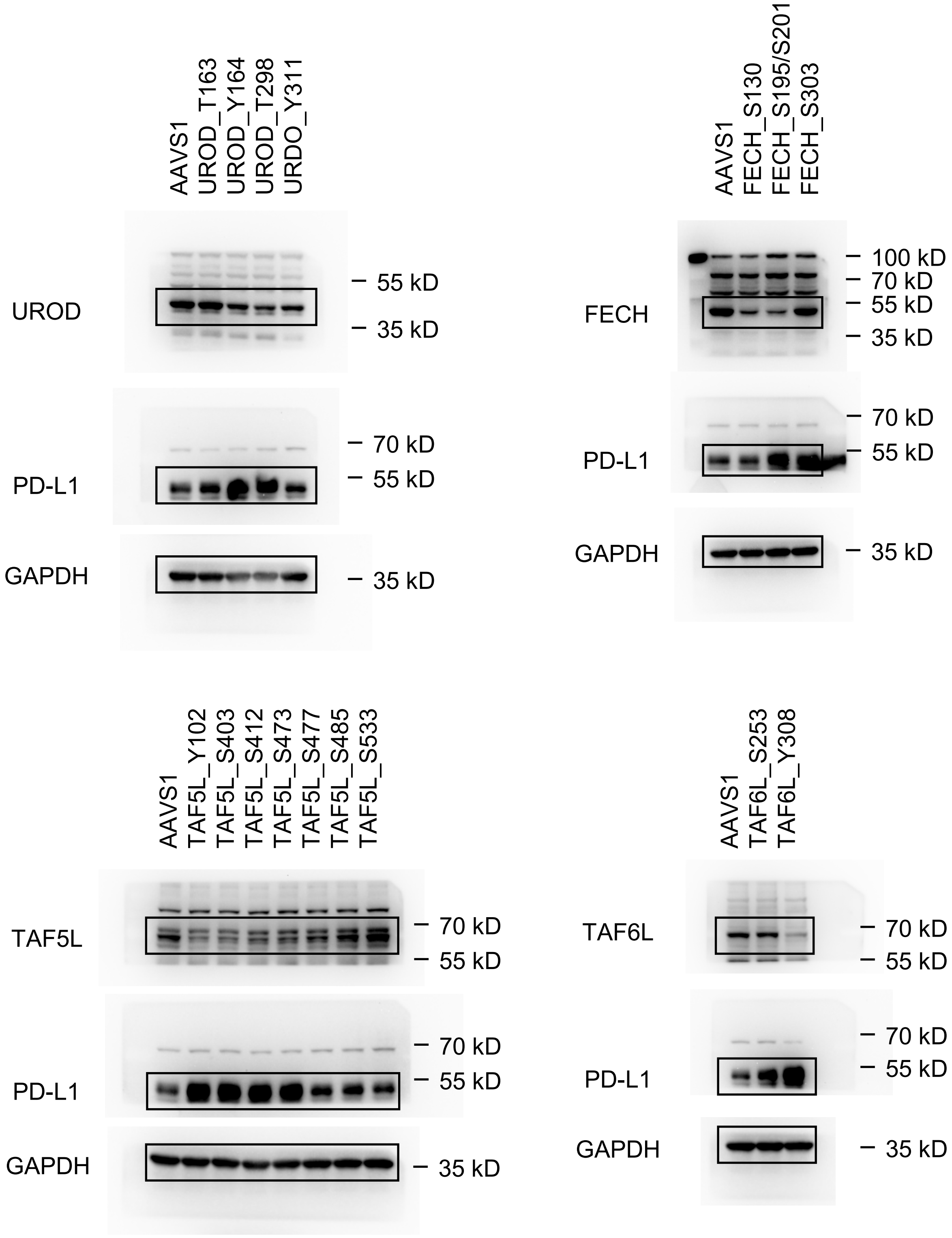

AAVS1
UROD_T163
UROD_Y164
UROD_T298
URDO_Y311
UROD
55 kD
35 kD
PD-L1
70 kD
55 kD
GAPDH
35 kD
AAVS1
FECH_S130
FECH_S195/S201
FECH_S303
100 kD
70 kD
55 kD
35 kD
FECH
PD-L1
70 kD
55 kD
GAPDH
35 kD
AAVS1
TAF5L_Y102
TAF5L_S403
TAF5L_S412
TAF5L_S473
TAF5L_S477
TAF5L_S485
TAF5L_S533
TAF5L
70 kD
55 kD
PD-L1
70 kD
55 kD
GAPDH
35 kD
AAVS1
TAF6L_S253
TAF6L_Y308
TAF6L
70 kD
55 kD
PD-L1
70 kD
55 kD
GAPDH
35 kD

Figure. S2

c

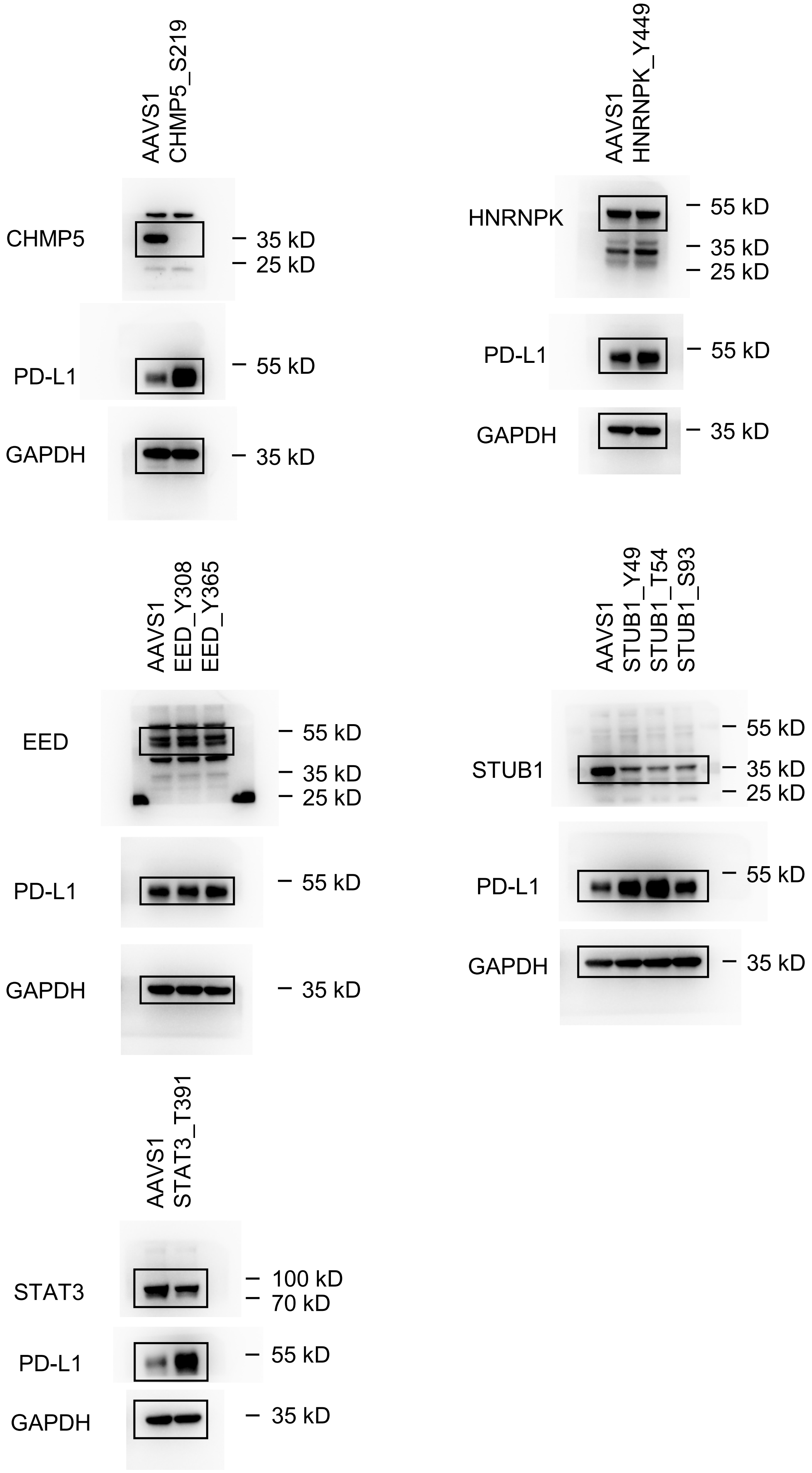
AAVS1
CHMP5_S219
CHMP5
35 kD
25 kD
PD-L1
55 kD
GAPDH
35 kD
AAVS1
HNRNPK_Y449
HNRNPK
55 kD
35 kD
25 kD
PD-L1
55 kD
GAPDH
35 kD
AAVS1
EED_Y308
EED_Y365
EED
55 kD
35 kD
25 kD
PD-L1
55 kD
GAPDH
35 kD
AAVS1
STUB1_Y49
STUB1_T54
STUB1_S93
55 kD
STUB1
35 kD
25 kD
PD-L1
55 kD
GAPDH
35 kD
AAVS1
STAT3_T391
STAT3
100 kD
70 kD
PD-L1
55 kD
GAPDH
35 kD

Figure. S2

d

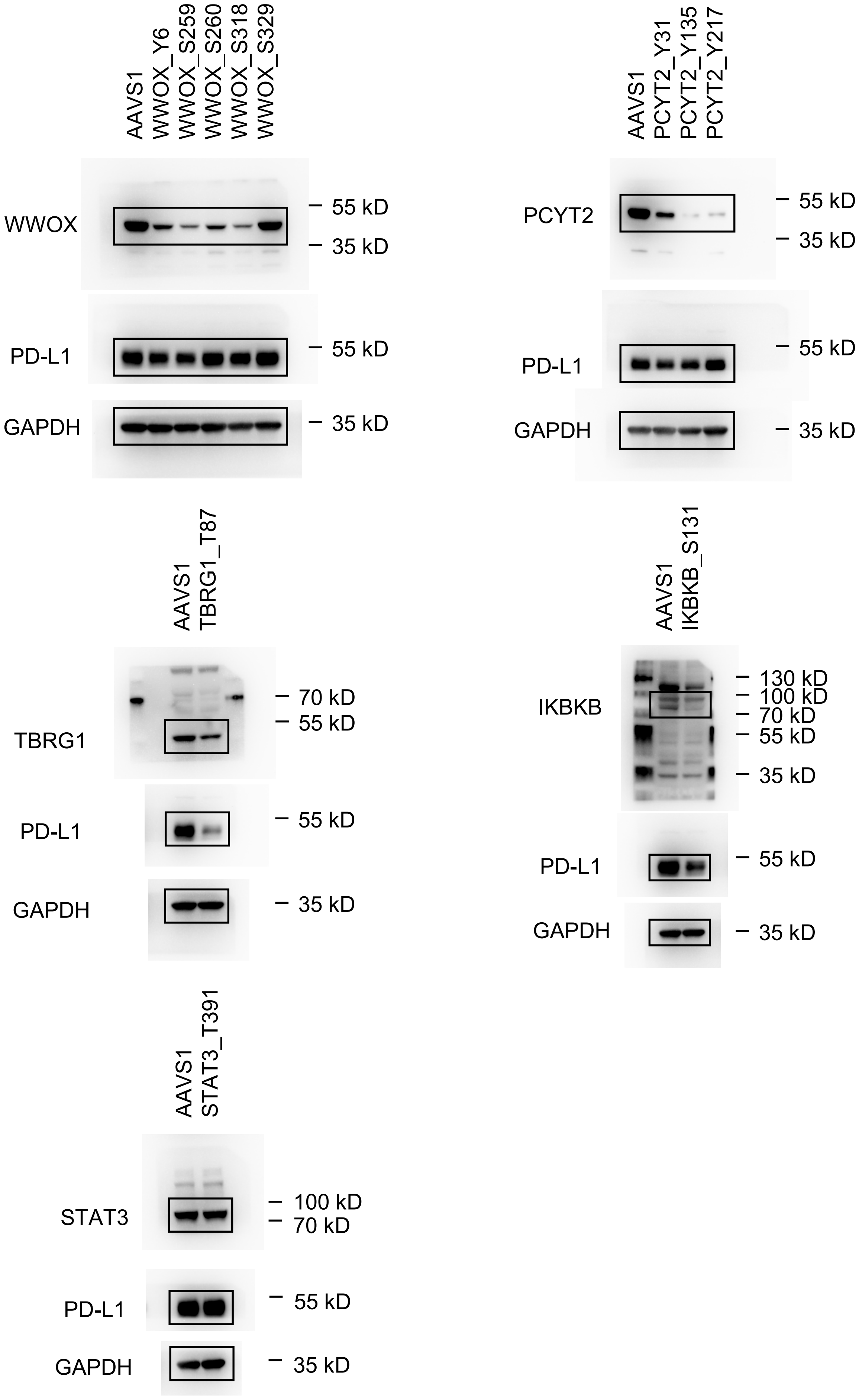
AAVS1
WWOX_Y6
WWOX_S259
WWOX_S260
WWOX_S318
WWOX_S329
WWOX
55 kD
35 kD
PD-L1
55 kD
GAPDH
35 kD
AAVS1
PCYT2_Y31
PCYT2_Y135
PCYT2_Y217
PCYT2
55 kD
35 kD
PD-L1
55 kD
GAPDH
35 kD
AAVS1
TBRG1_T87
TBRG1
70 kD
55 kD
PD-L1
55 kD
GAPDH
35 kD
AAVS1
IKBKB_S131
IKBKB
130 kD
100 kD
70 kD
55 kD
35 kD
PD-L1
55 kD
GAPDH
35 kD
AAVS1
STAT3_T391
STAT3
100 kD
70 kD
PD-L1
55 kD
GAPDH
35 kD

Figure. S3

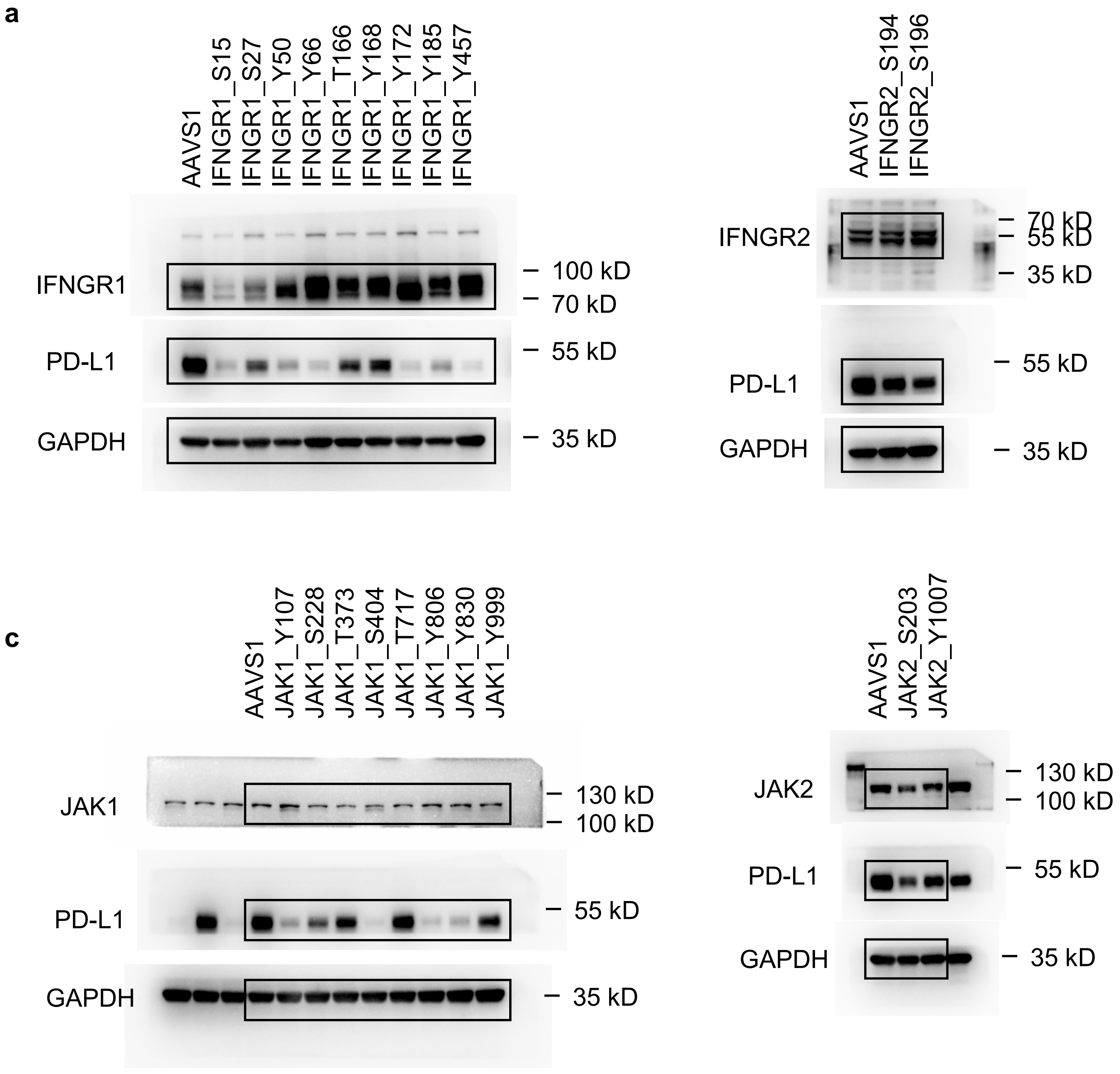
a
AAVS1
IFNGR1_S15
IFNGR1_S27
IFNGR1_Y50
IFNGR1_Y66
IFNGR1_T166
IFNGR1_Y168
IFNGR1_Y172
IFNGR1_Y185
IFNGR1_Y457
IFNGR1
PD-L1
GAPDH
100 kD
70 kD
55 kD
35 kD
AAVS1
IFNGR2_S194
IFNGR2_S196
IFNGR2
PD-L1
GAPDH
70 kD
55 kD
35 kD
55 kD
35 kD
c
AAVS1
JAK1_Y107
JAK1_S228
JAK1_T373
JAK1_S404
JAK1_T717
JAK1_Y806
JAK1_Y830
JAK1_Y999
JAK1
PD-L1
GAPDH
130 kD
100 kD
55 kD
35 kD
AAVS1
JAK2_S203
JAK2_Y1007
JAK2
PD-L1
GAPDH
130 kD
100 kD
55 kD
35 kD

Figure. S3

e

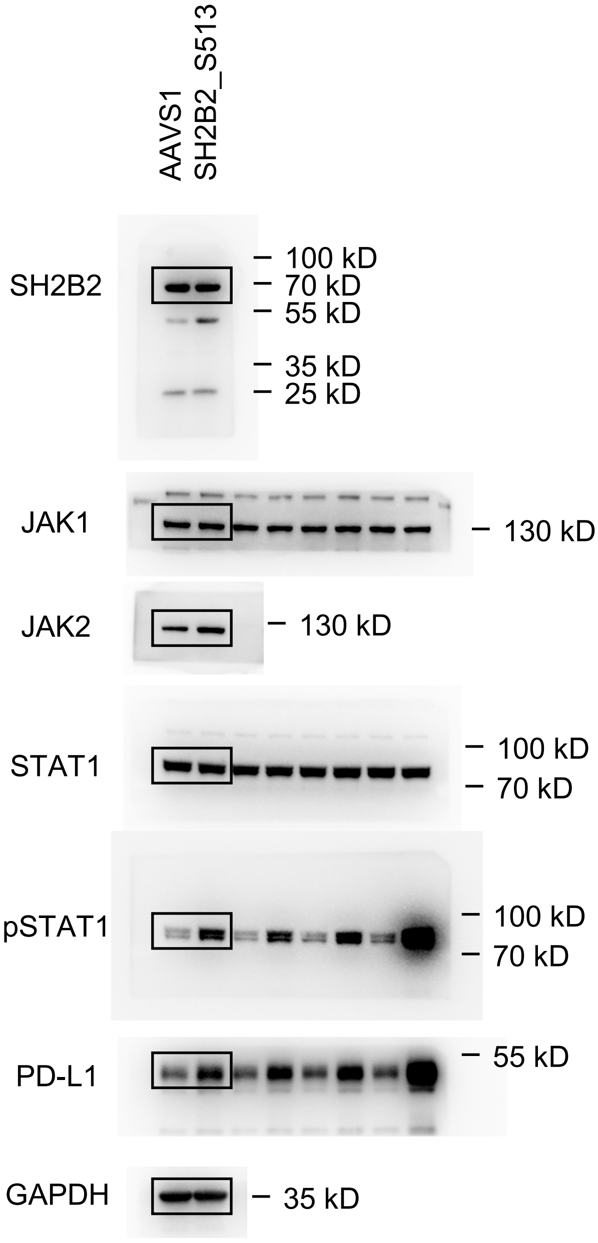
AAVS1
SH2B2_S513
SH2B2
100 kD
70 kD
55 kD
35 kD
25 kD
JAK1
130 kD
JAK2
130 kD
STAT1
100 kD
70 kD
pSTAT1
100 kD
70 kD
PD-L1
55 kD
GAPDH
35 kD

f

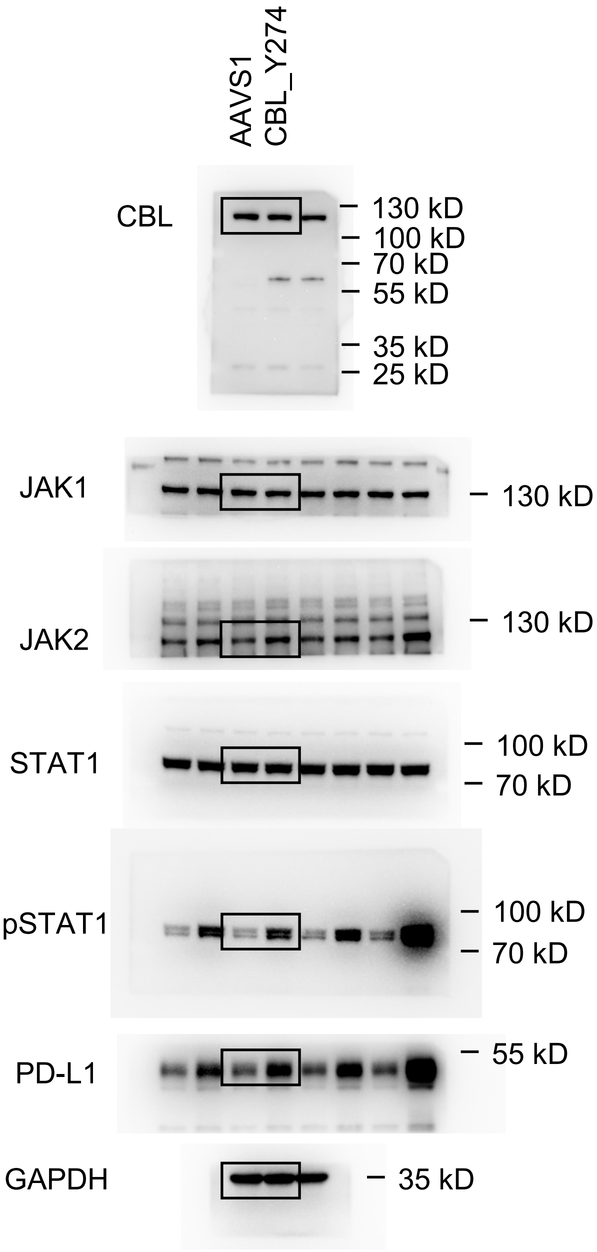
AAVS1
CBL_Y274
CBL
130 kD
100 kD
70 kD
55 kD
35 kD
25 kD
JAK1
130 kD
JAK2
130 kD
STAT1
100 kD
70 kD
pSTAT1
100 kD
70 kD
PD-L1
55 kD
GAPDH
35 kD

Figure. S3

g

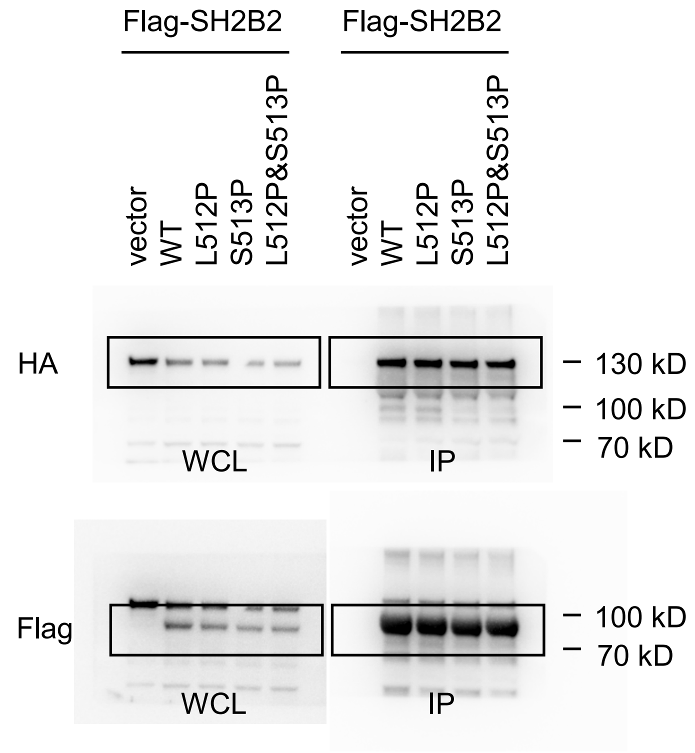

Flag-SH2B2
Flag-SH2B2
vector
WT
L512P
S513P
L512P&S513P
vector
WT
L512P
S513P
L512P&S513P
HA
130 kD
100 kD
70 kD
WCL
IP
Flag
100 kD
70 kD
WCL
IP

h

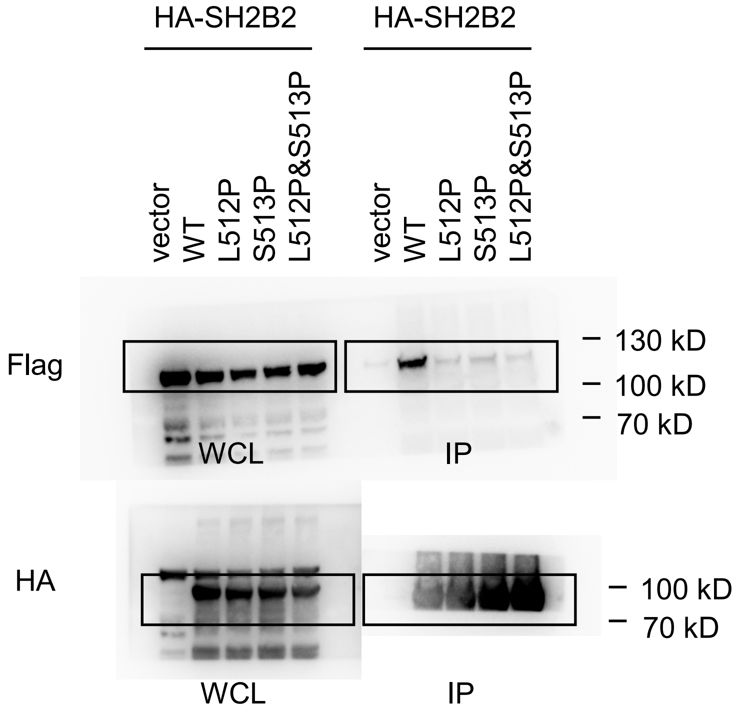

HA-SH2B2
HA-SH2B2
vector
WT
L512P
S513P
L512P&S513P
vector
WT
L512P
S513P
L512P&S513P
Flag
130 kD
100 kD
70 kD
WCL
IP
HA
100 kD
70 kD
WCL
IP

Figure. S3

i

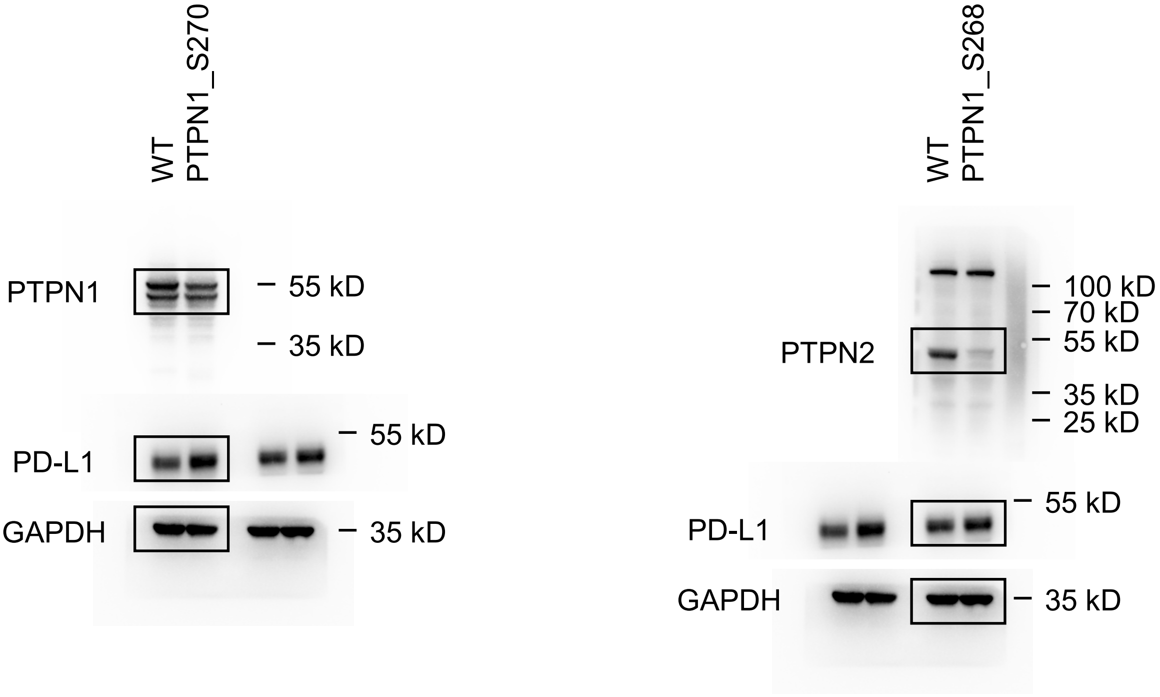
WT
PTPN1_S270
PTPN1
55 kD
35 kD
PD-L1
55 kD
GAPDH
35 kD
WT
PTPN1_S268
100 kD
70 kD
55 kD
PTPN2
35 kD
25 kD
PD-L1
55 kD
GAPDH
35 kD

Figure. S3

j

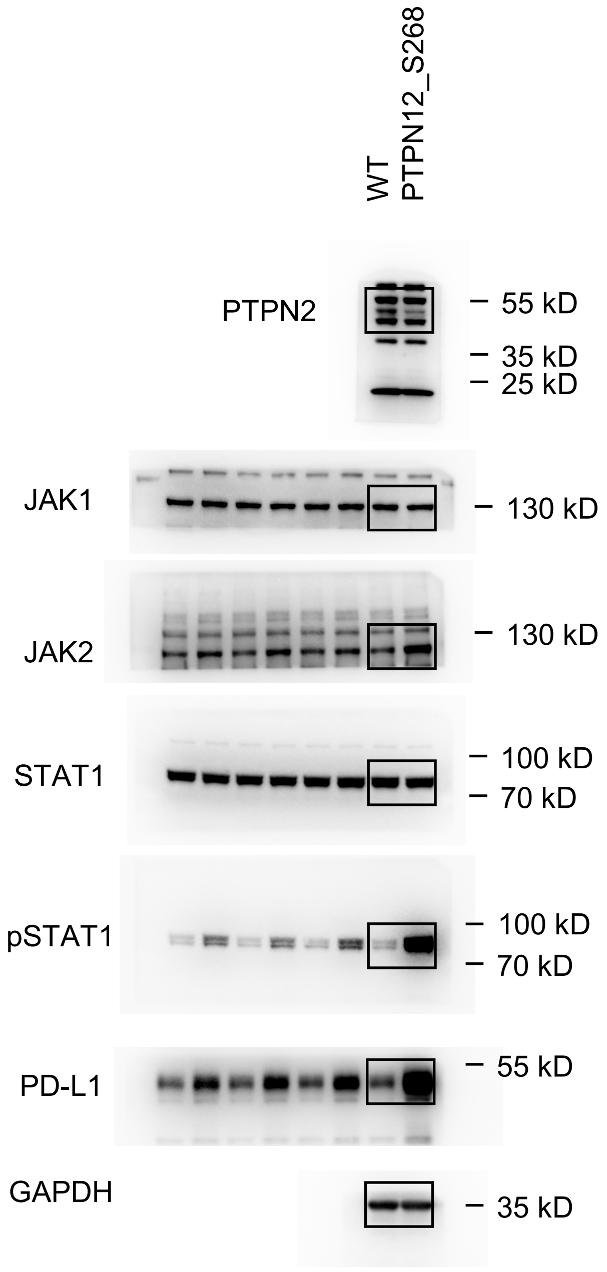
WT
PTPN12_S268
PTPN2
55 kD
35 kD
25 kD
JAK1
130 kD
JAK2
130 kD
STAT1
100 kD
70 kD
pSTAT1
100 kD
70 kD
PD-L1
55 kD
GAPDH
35 kD

Figure. S3

k

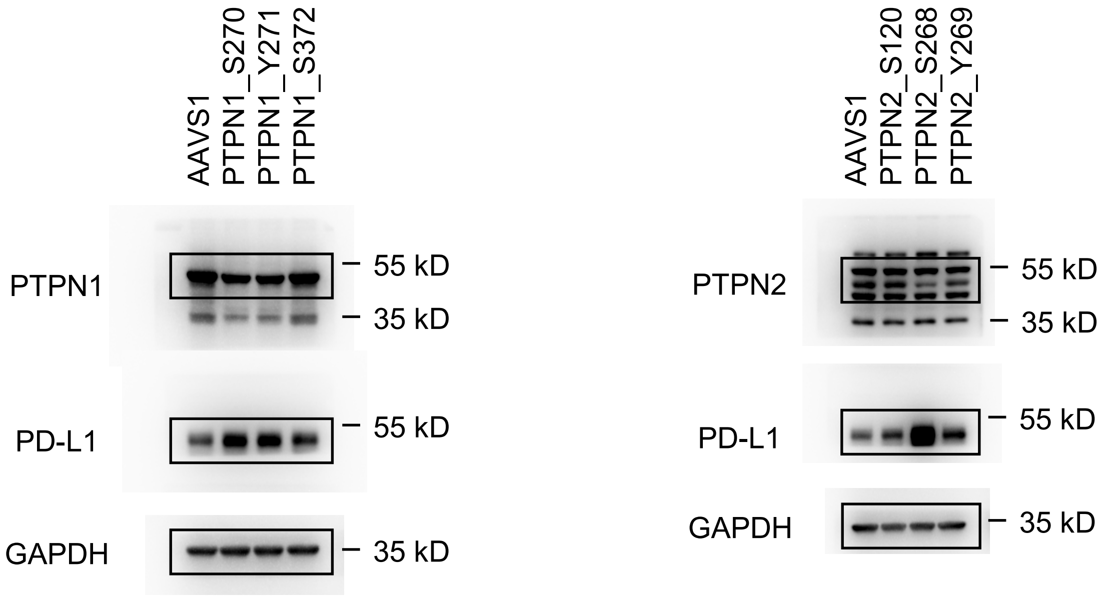
AAVS1
PTPN1_S270
PTPN1_Y271
PTPN1_S372
PTPN1
55 kD
35 kD
PD-L1
55 kD
GAPDH
35 kD
AAVS1
PTPN2_S120
PTPN2_S268
PTPN2_Y269
PTPN2
55 kD
35 kD
PD-L1
55 kD
GAPDH
35 kD

l

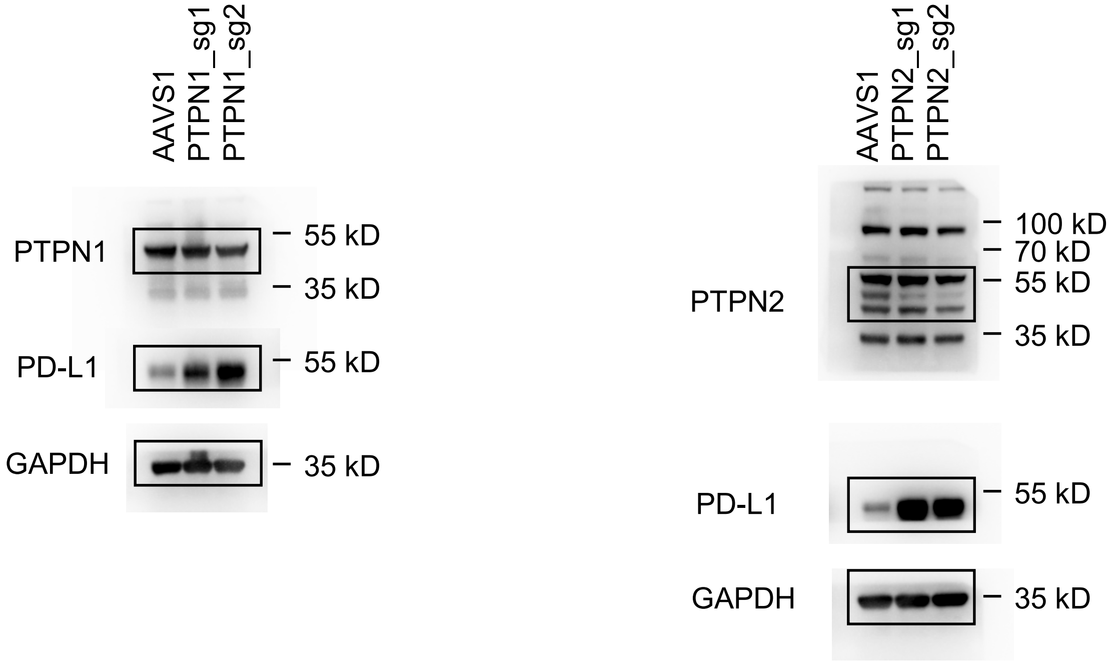
AAVS1
PTPN1_sg1
PTPN1_sg2
PTPN1
55 kD
35 kD
PD-L1
55 kD
GAPDH
35 kD
AAVS1
PTPN2_sg1
PTPN2_sg2
PTPN2
100 kD
70 kD
55 kD
35 kD
PD-L1
55 kD
GAPDH
35 kD

Figure. S7

c

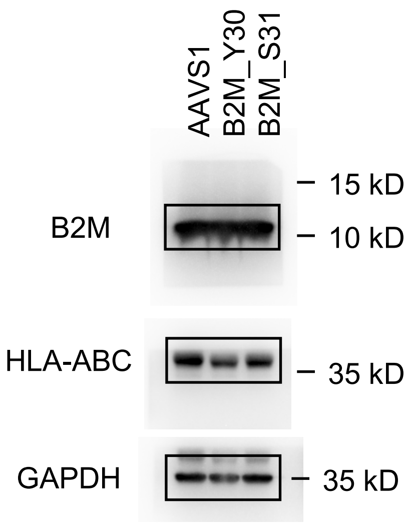

f

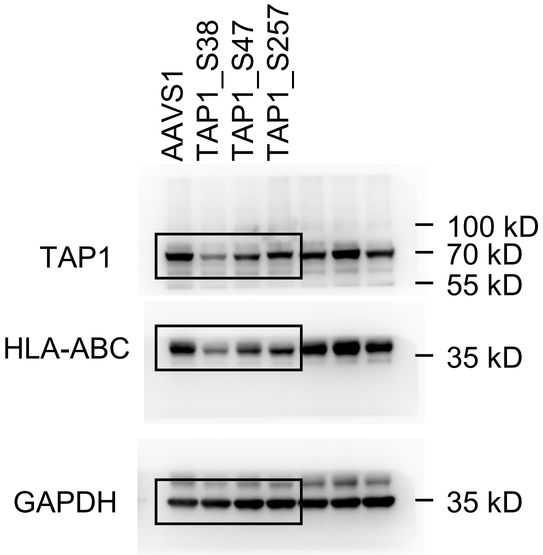

g

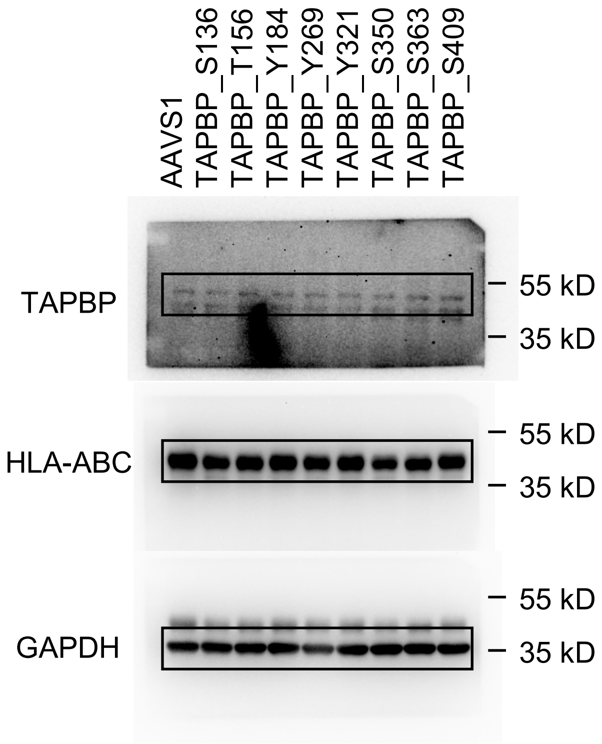

Figure. S7

h

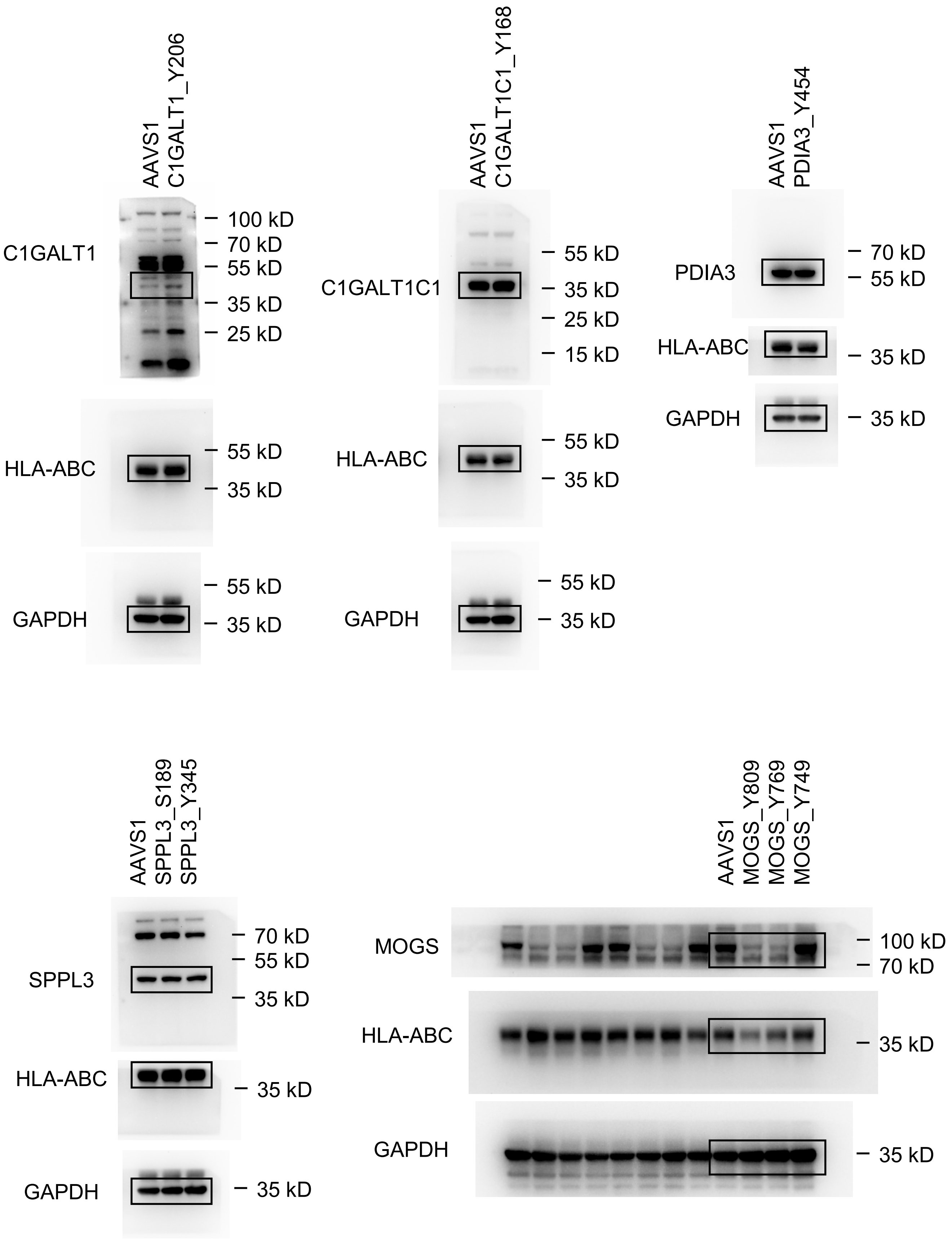
AAVS1
C1GALT1_Y206
C1GALT1
100 kD
70 kD
55 kD
35 kD
25 kD
HLA-ABC
55 kD
35 kD
GAPDH
55 kD
35 kD
AAVS1
C1GALT1C1_Y168
C1GALT1C1
55 kD
35 kD
25 kD
15 kD
HLA-ABC
55 kD
35 kD
GAPDH
55 kD
35 kD
AAVS1
PDIA3_Y454
PDIA3
70 kD
55 kD
HLA-ABC
35 kD
GAPDH
35 kD
AAVS1
SPPL3_S189
SPPL3_Y345
SPPL3
70 kD
55 kD
35 kD
HLA-ABC
35 kD
GAPDH
35 kD
AAVS1
MOGS_Y809
MOGS_Y769
MOGS_Y749
MOGS
100 kD
70 kD
HLA-ABC
35 kD
GAPDH
35 kD

Figure. S8

d

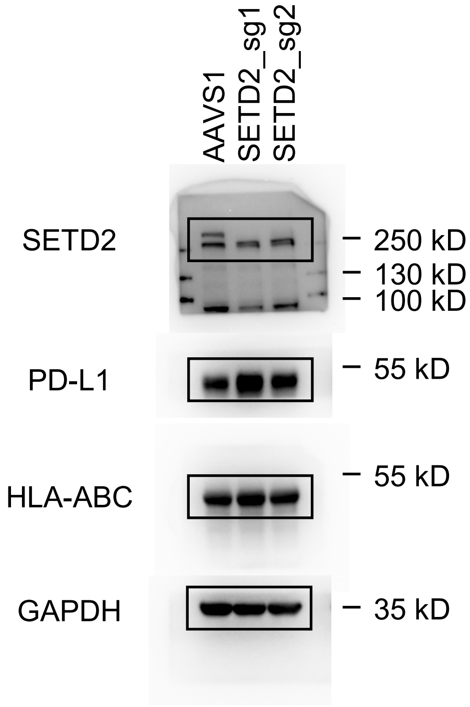
AAVS1
SETD2_sg1
SETD2_sg2
SETD2
250 kD
130 kD
100 kD
PD-L1
55 kD
HLA-ABC
55 kD
GAPDH
35 kD
